# Adaptive and maladaptive introgression in grapevine domestication

**DOI:** 10.1073/pnas.2222041120

**Published:** 2023-06-05

**Authors:** Hua Xiao, Zhongjie Liu, Nan Wang, Qiming Long, Shuo Cao, Guizhou Huang, Wenwen Liu, Yanling Peng, Summaira Riaz, Andrew M. Walker, Brandon S. Gaut, Yongfeng Zhou

**Affiliations:** ^a^State Key Laboratory of Tropical Crop Breeding, Shenzhen Branch, Guangdong Laboratory of Lingnan Modern Agriculture, Key Laboratory of Synthetic Biology, Ministry of Agriculture and Rural Affairs, Agricultural Genomics Institute at Shenzhen, Chinese Academy of Agricultural Sciences, Shenzhen 518000, China; ^b^The State Key Laboratory of Genetic Improvement and Germplasm Innovation of Crop Resistance in Arid Desert Regions, Key Laboratory of Genome Research and Genetic Improvement of Xinjiang Characteristic Fruits and Vegetables, Institute of Horticultural Crops, Xinjiang Academy of Agricultural Sciences, Urumqi 830091, China; ^c^Department of Viticulture and Enology, University of California, Davis, CA 95616; ^d^Department of Ecology and Evolutionary Biology, University of California, Irvine, CA 92697; ^e^State Key Laboratory of Tropical Crop Breeding, Tropical Crops Genetic Resources Institute, Chinese Academy of Tropical Agricultural Sciences, Haikou 571101, China

**Keywords:** population genetics, grape breeding, machine learning, viticulture, structural variation

## Abstract

Domesticated grapevines spread to Europe around 3,000 years ago. Previous studies have revealed genomic signals of introgression from wild to cultivated grapes in Europe, but the time, mode, genomic pattern, and biological effects of these introgression events have not been investigated. Here, we studied resequencing data from 345 samples spanning the distributional range of wild (*Vitis vinifera* ssp. *sylvestris*) and cultivated (*V. vinifera* ssp. *vinifera*) grapes. Based on machine learning–based population genetic analyses, we detected evidence for a single domestication of grapevine, followed by continuous gene flow between European wild grapes (EU) and cultivated grapes over the past ~2,000 y, especially from EU to wine grapes. We also inferred that soft-selective sweeps were the dominant signals of artificial selection. Gene pathways associated with the synthesis of aromatic compounds were enriched in regions that were both selected and introgressed, suggesting EU wild grapes were an important resource for improving the flavor of cultivated grapes. Despite the potential benefits of introgression in grape improvement, the introgressed fragments introduced a higher deleterious burden, with most deleterious SNPs and structural variants hidden in a heterozygous state. Cultivated wine grapes have benefited from adaptive introgression with wild grapes, but introgression has also increased the genetic load. In general, our study of beneficial and harmful effects of introgression is critical for genomic breeding of grapevine to take advantage of wild resources.

## Significance

Our study focused on the history of introgression between domesticated grapes and their European wild relative. We find evidence for a single domestication of grapevine with introgression from the wild relative to wine, but not to table, grapes. These regions are enriched for genes related to aromatic compound synthesis, suggesting that European wild grapes have been an important resource for improving the flavor of wine grapes. However, the beneficial effects of introgression carry a potential cost because introgressed regions have elevated numbers of putatively deleterious variants, which are usually maintained in a heterozygous state for this clonally propagated crop. The identification of introgressed beneficial and deleterious variants is important for genomic breeding of grapevines.

Introgression describes the movement of alleles, usually via hybridization and backcrossing, from the donor species or population into the recipient species or population (1, 2). It is a pervasive evolutionary process that impacts both population fitness (3, 4) and the genomic landscape. However, the outcome of individual introgression events is shaped by the relative strength of evolutionary processes, meaning that the outcome of individual introgression events can vary considerably. In some cases—i.e., when there is a balance between repeated introgression and weak selection against introgressed variants—the introgressed allele is expected to reach an equilibrium population frequency (5, 6). More often, it is reasonable to expect that an introgressed allele is maladaptive and hence purged by natural selection (7). Genetic drift can, however, contravene natural selection and lead to the maintenance or fixation of maladaptive introgressed fragments (8).

Of course, introgression can introduce adaptive variants, too. Adaptive introgression is expected to proceed when alleles from the donor species have a positive effect on the fitness of the recipient species (9, 10). One possible positive effect is that the introgressed allele reduces the genetic load in the recipient population. This may be especially true when deleterious alleles are recessive (so that a heterozygous hybrid has an immediate advantage), when the donor population has a larger population size (and hence likely houses fewer deleterious mutations), and for genomic regions that have high recombination rates (where interference among mutations is minimized) (11). There is also growing recognition that adaptive introgression can reassort genetic variants into beneficial combinations, thereby driving local adaptation into new ecological niches (12) and distinct habitats (9). Current evidence suggests, for example, that introgressed variants have contributed to toxin resistance in Gulf killifish, allowing it to occupy polluted habitats (12). When an introgressed fragment is adaptive, it can also include linked, deleterious variants that will hitchhike to fixation, especially in regions of low recombination. The linked selection of deleterious variants may explain some features of introgression from Neanderthal to humans because introgressed fragments convey increased risks to humans for some genetic diseases (13).

Although it is widely appreciated that introgression can contribute to local adaptation, our understanding of the consequences, genetic architecture, and traits affected by adaptive introgression is still quite limited. Here, we are interested in introgression between a crop wild relative (CWR) and a crop because there is a growing recognition that historic introgression events have been sources for key agronomic traits (14). Introgression between CWRs and crops may be especially likely because barriers to hybridization are incomplete due to a short divergence time between taxa, and this is especially true for perennial crops with long generation times, like grapevines (15–17). In addition, introgression remains a common breeding tool for introducing beneficial alleles from a CWR into a crop (18), especially for traits like disease and stress resistance, making CWRs important sources for crop improvement and for maintaining food security (19). These considerations argue that it is important to characterize genomic patterns of introgression between a domesticate and CWRs. Indeed, CWR-to-crop gene flow has received growing attention over the past decade (20–22).

Here, we investigate population genomic patterns of introgression between the domesticated grapevine (*Vitis vinifera* ssp *vinifera*; hereafter *vinifera*) and its wild progenitor (*V. vinifera* ssp *sylvestris*; hereafter *sylvestris*). Grapevine is among the first domesticated crops; it has been widely cultivated for both fruit (table grapes) and wine since antiquity. Historical and archaeological evidence dated the first domestication of grapevines back to 6,000 to 5,800 BC, when ancestors began to collect and propagate *sylvestris* in regions that may have included the South Caucasus, the northern Fertile Crescent, and the Levant (23–27). After their domestication, grapes were spread throughout the Mediterranean world, leading to the diversification of many locally adapted varieties that are typically clonally propagated (28, 29). Within the last 3,000 y (30, 31), grapevines were established in Europe, where there was secondary contact with genetically distinct *sylvestris* populations. Previous studies have found evidence to suggest a history of introgression between *vinifera* and European populations of *sylvestris* (16, 26–29, 32). However, the genomic extent and fitness effects of introgressed regions remain largely uncharacterized in grapevines and other crops.

We analyze a set of 345 sequenced accessions that represent both the diversity of cultivated grapevine, including wine and table grapes, and the broad geographic distribution of *sylvestris*. We used these data to focus on four sets of questions. First, we assess patterns of divergence among *sylvestris* populations, wine grapes, and table grapes to get insight into population and domestication history. Second, we use machine learning methods to identify introgressed regions of the *vinifera* genome. What are the genomic characteristics of these regions? And do they correspond to selected regions of the genome, which we have also inferred using machine learning approaches? Third, are introgressed regions replete with putatively deleterious variants, consistent with hitchhiking with adaptive variants, or is it likely that introgression occurred to reduce the deleterious load? Finally, what traits may have been affected by introgression, and is there putative evidence of supergenes—i.e., introgressed linked, adaptive complexes? Altogether, using machine learning–based population genetic methods, this study distinguishes the signals of soft/hard sweeps, the time, mode, direction, and content of introgression, while providing insights into their genomic signatures and their implications for grapevine breeding.

## Results

### Population Subdivision and Heterozygosity.

To investigate the genetic history of grapevines, including introgression events, we analyzed resequencing data from a total of 345 *Vitis* accessions. The accessions included 72 wild grapes (*V. vinifera* ssp. *sylvestris*) from Europe (EU), 36 wild grapes from the Middle East and Caucasus region (ME), and 231 domesticated grapes (*V. vinifera* ssp. *vinifera*), along with representatives of North American outgroup species *Vitis californica* (n = 3) and *Muscadinia rotundifolia* (n = 3). Wild *sylvestris* was sampled to cover the predicted distribution area in Europe and the Near East (*SI Appendix*, Fig. S1). Among the analyzed sample, 40 accessions were resequenced by this study; the remaining data were published previously (*SI Appendix*, Table S1). The 345 accessions had average mapping coverages of >20x.

**Fig. 1.**
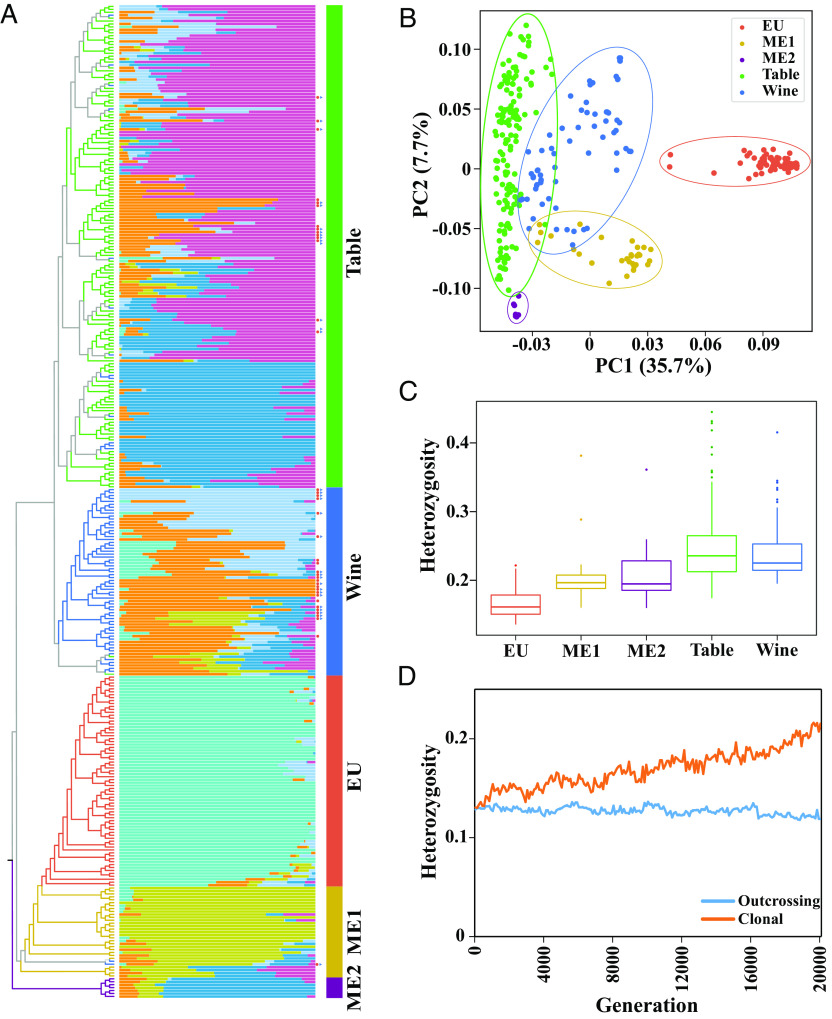
Relationship among groups and their heterozygosity. (*A*) A phylogenetic tree with admixture analysis. In the phylogeny, the color of branches reflects different groups: ME1, yellow; ME2, purple; EU, reddish brown; wine, blue; table, green. The admixture plot is with *K *= 6. The red dots and blue triangles to the right of the admixture plot show whether chloroplasts or mitochondria, respectively, in accessions from the table or wine groups had an apparent origin from EU *sylvestris* grapes. (*B*) A PCA supporting the designation of five groups. (*C*) Heterozygosity within the five groups. (*D*) The results of forward simulations under different types of propagation. The blue line indicates an outcrossing population, while the orange line represents clonal propagation.

After calling and filtering SNPs for all samples relative to the Chardonnay reference (33), we constructed an ML phylogenetic tree of the samples based on different models (Fig. 1*A* and *SI Appendix*, Fig. S2; see *Material and Methods*). In the resulting phylogeny, domesticated grapes clustered together, suggesting a single domestication event, consistent with some but not all previous studies (34). Wine and table grapes were reciprocally monophyletic, suggesting an early divergence of domesticated grapes based on usage. The *sylvestris* samples were also monophyletic, but *sylvestris* contained three distinct groups: those collected in Europe (EU group), those around the Caspian Sea (ME1 group), and samples from the Fertile Crescent near the Mediterranean Sea (ME2 group) (35). A principal component analysis (PCA) and the estimation of the ancestry component proportion also confirmed the divergence among these populations (Fig. 1*B* and *SI Appendix*, Figs. S3–S5). Notably, the EU *sylvestris* formed a distinct group but with minority admixture components shared with wine grapes (Fig. 1*A*).

To gain more potential insight into the possibility of hybridization and introgression among groups, we constructed network phylogenies based on chloroplast and mitochondrial genome data (*SI Appendix*, Figs. S6 and S7). These cytoplasmic phylogenies had three notable features: 1) Some domesticated grape varieties clustered with the EU group, but more obviously for wine grapes (32.5% and 28.4%, based on chloroplast and mitochondrial trees, respectively) than for table grapes (9.3% and 11.8%), thus supporting the admixture pattern based on nuclear genomes; 2) the cytoplasmic genomes of wild ME1 and ME2 groups were indistinguishable from the remainder of domesticated grapes; and 3) wine and table grapes were intercalated in clades, punctuated by short branches between them. These results, especially the grouping of wine grapes with EU *sylvestris*, suggest the possibility of extensive introgression.

### Demography and the Evidence for Introgression.

To investigate divergence between wild and domesticated grapes, we evaluated heterozygosity and genome-wide differentiation (*F_ST_*). Wine and table grapes (0.24 in both) had higher heterozygosity than wild populations (0.17 in EU, 0.20 for ME1, 0.22 for ME2, Fig. 1*C*), which could be due to historical introgressions and long-term clonal propagation that permits the accumulation of heterozygous mutations (30, 33, 36). We used forward simulations to evolve outcrossed and clonal populations. The results showed that outcrossing populations maintain an equilibrium (heterozygosity is 0.13 ± 0.004), but clonal populations increase heterozygosity continuously through 20,000 asexual generations (Fig. 1*D*), suggesting that clonality is one factor contributing to differences in heterozygosity between wild and cultivated populations.

**Fig. 2.**
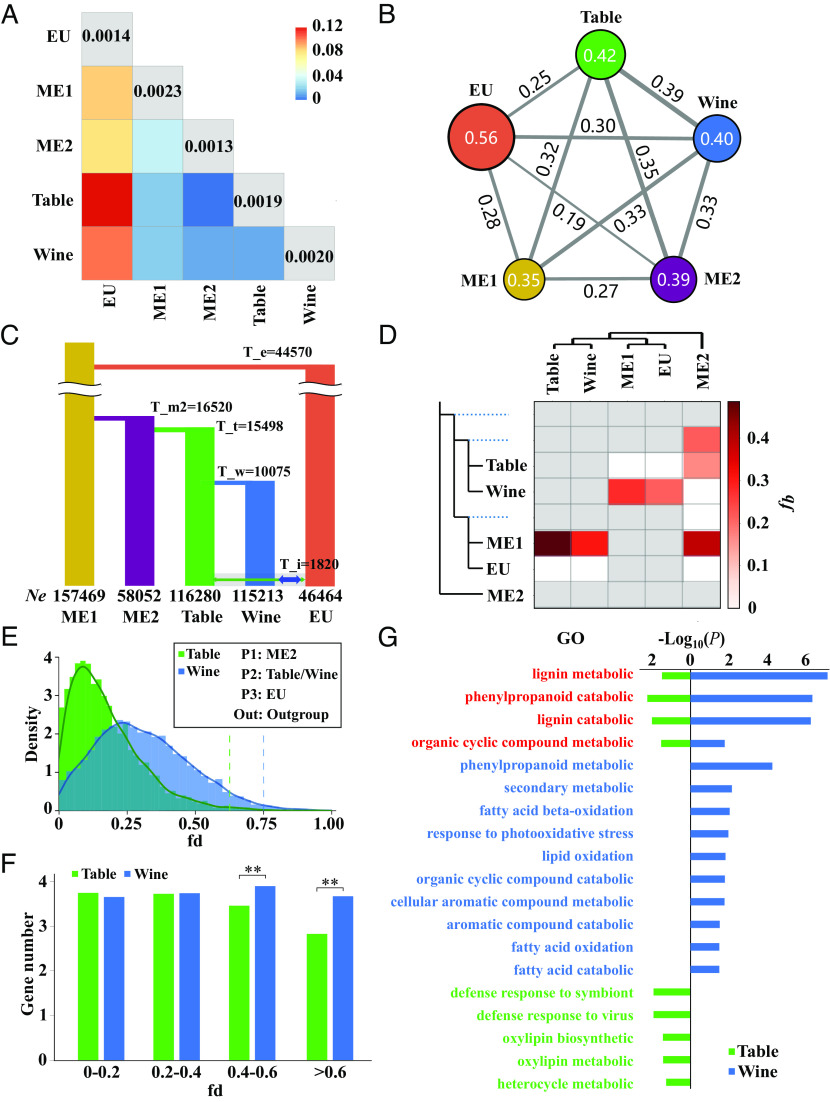
Demographic inference and detection of introgression. (*A*) Genome-wide *F_ST_* and π for each group. The heat map reflects overall *F_ST_* values between each group, with warmer colors representing higher divergence. The values in the diagonal cells report nucleotide diversity (π) within each group. (*B*) Identity-by-descent (IBD) within and between groups. The values within circles indicate the average IBD between individuals in each group, with larger circles reflecting higher values. The values on the gray lines report the average IBD between individuals in each group. Thicker lines reflect higher values. (*C*) The best-fitting model was inferred through fastsimcoal analysis. The time (T) in the horizontal branch is the divergence time (year) of each group, with T_i representing the inferred onset of introgression. The numbers at the bottom report the estimated effective population size (*Ne*) of each group. (*D*) Values of the branch introgression (fb) statistic represent the potential signal between groups. Darker and warmer colors represent higher fb values. (*E*) Kernel density estimation of fd values in wine and table, based on 50-kb windows. The green vertical line denotes the highest 5% fd regions of the table group, with the blue vertical line indicating the 5% fd values of the wine group. (*F*) The average gene number in regions within bins of fd values for the wine or table group. Asterisks denote a significant difference (**P < 0.01, Student’s *t* test) between wine and table groups in each fd bin. (*G*) GO annotation of genes in the highest 5% fd regions of wine and table groups. Red text shows GO annotation labels shared by wine and table groups; blue text is GO annotation specific for the wine group, and green text is specific to the table group. The *x *axis denotes the *P*-value on the log_10_ scale.

Regarding divergence between groups, the average genome-wide *F_ST_* between either ME1 or ME2 and cultivated grapes ranged from 0.006 to 0.036 (Fig. 2*A* and *SI Appendix*, Fig. S8). In contrast, the wild EU group had higher pairwise *F_ST_* values compared to the other groups (from 0.085 to 0.120). Comparable results were obtained in sequence similarity (*D_xy_*) analysis among five populations (*SI Appendix*, Fig. S9). Notably, ME2 also had the lowest within-group nucleotide diversity (π = 0.0013 on average) at ~2/3 the value of ME1 (0.0023 on average), which had the highest within-group nucleotide diversity (Fig. 2*A* and *SI Appendix*, Fig. S10).

To further investigate population histories, we performed two additional analyses: i) sequential Markov coalescent (SMC) analysis and ii) identity-by-descent (IBD) analysis. SMC++ analysis revealed that all populations experienced bottlenecks with continual decreases in population sizes until ~10^4^ B.P. (*SI Appendix*, Fig. S11), consistent with previous studies (26, 30). We calculated IBD within each population and in pairs between populations. Higher IBD values reflect higher probabilities of recent ancestry. Not surprisingly, given its lower diversity, the EU group had the highest within-group IBD value (0.56), while the remaining groups had similar values (ranging from 0.35 to 0.42). EU also tended to have lower pairwise IBD values compared to the remaining populations, reflecting higher divergence, although the EU-wine comparison had notably higher IBD values than the other EU-based pairwise contrasts (Fig. 2*B*).

These results might indicate an introgression history between wine grapes and EU *sylvestris*. To address this issue more directly, we modeled demography using site frequency spectra (2dSFS) and coalescent simulations (*Material and Methods*). We performed demographic estimation of each population independently (*SI Appendix*, Figs. S12–S17 and Table S2) and compared six initial models without introgression to find out the most likely relationships between cultivated grapes (table and wine) and ME wild grapes (ME1 or ME2) (*SI Appendix*, Fig. S18 and Table S3). Similar to the SMC++ results, fastsimcoal inferred a bottleneck with subsequent recovery for the EU, table, and wine groups. In contrast, it inferred a continuous *Ne* decrease for both ME1 and ME2.

Based on the initial model, we estimated 34 different potential models of gene flow among the table, wine, and EU populations (*SI Appendix*, Figs. S19 and S20 and Table S4). Under the best model, fastsimcoal inferred that the EU group diverged ~4 × 10^4^ years ago; suggested that the domestication of table grapes occurred as early as ~1.5 × 10^4^ y ago (Fig. 2*C*); and estimated that wine grapes split from table grapes ~1.0 × 10^4^ years ago. This fastsimcoal model suggested that gene flow between EU and domesticated populations began 1.8 × 10^3^ years ago. Importantly, the best-fit model also suggested a high probability of gene flow from EU *sylvestris* into domesticated populations, with ~fivefold higher migration rates into wine grapes (1.7 × 10^−4^) compared to table grapes (3.8 × 10^−5^) (Fig. 2*C* and *SI Appendix*, Table S5).

To complement analyses of introgression patterns between wild grapes (EU, ME1, and ME2) and domesticated grapes (table and wine) inferred from coalescent models, we also calculated the fb statistic (37), which was based on triplet topologies that were rooted with whole-genome data from outgroups (*Vitis californica* and *Muscadinia rotundifolia*) (Fig. 2*D*). The results reflected no evidence for introgression between the EU and table groups (fb = 0), but the fb value (0.22) between the EU and wine groups was consistent with the possibility of introgression. Overall, our analyses are consistent with previous analyses in suggesting that domestication from ME groups was followed by introgression between EU and wine grapes (26, 28, 29). Our results are, however, bolstered by additional detail about the timing and genomic locations of these events (see below).

### Introgression Signals from EU to Wine Grapes.

Given evidence for introgression between EU *sylvestris* and domesticated grapes, we also applied the fd statistic based on 50-kb nonoverlapping windows. As with fb, the triplet topologies suggested a higher genome-wide value for the EU-wine comparison (average fd of 0.26) than the EU-table comparison (average fd of 0.06), again suggesting higher levels of introgression between EU *sylvestris* and wine grapes compared to table grapes (Fig. 2*E* and *SI Appendix*, Fig. S21). However, the difference in genome-wide fd values between wine and table grapes could, in principle, reflect population parameters like *Ne*, recombination, and mating systems. We, therefore, used *D_xy_* to analyze potentially introgressed regions of wine-EU and table-EU pairs. We measured the difference (*D_xy_* (wine_EU)–*D_xy_* (table_EU)) of each window based on the triplet topologies between EU groups and domesticated populations (*SI Appendix*, Fig. S22). The average value of the difference between *D_xy_* (wine_EU) and *D_xy_* (table_EU) was −0.02, indicating that genomic sequences were more similar between wine and EU groups than between the table and EU groups. These results support our inference of EU introgression into wine grapes but provide no evidence for EU introgression with table grapes. The fdM statistic further confirmed this result because fdM identified 6,743 windows (50 kb per window) as admixed between EU and wine but only 651 windows between EU and table grapes (*SI Appendix*, Table S6).

We also calculated the number and function of genes in high fd windows. Comparing the wine-EU and table-EU pairs, we found no difference in gene numbers within low fd regions (fd < 0.4, *P* > 0.05). However, we found more genes in the high fd regions (>= 0.4) of wine grapes, suggesting a genetic basis for successful introgression events (Fig. 2*F*). We also performed gene ontology (GO) analysis of the high fd windows. For both wine and table grapes, putatively introgressed regions were related to metabolic pathways, which could contribute to flavor profiles (Fig. 2*G*). In table grapes, we identified two biological processes related to defense responses, similar to introgression among wild grapes (38). Finally, some biological processes were significantly enriched only in wine grapes, such as phenylpropanoid metabolic, secondary metabolic, and aromatic compounds. This last set of GO enrichment categories suggests that introgression may have affected aromatics for the winemaking process. We also evaluated the effects of recombination on introgression and found no significant difference in recombination between the 5% or 1% highest fd regions (0.0066 and 0.0064 cM/kb) and the genomic background (0.0066 cM/kb) (*SI Appendix*, Table S7).

### Machine Learning–Based Genome Scans of Selected and Introgressed Regions.

Although previous studies have detected introgression between EU *sylvestris* and wine grapes (26, 28, 29), these inferences have suffered from two shortcomings. First, they have generally not been put into the context of adaptive events to investigate the interplay between selection and introgression. Second, the boundaries of introgressed regions have not been located precisely.

To broadly survey the genetic features of domesticated grape genomes, we analyzed genome-wide selection by applying the population branch statistic (PBS) to wine and table grapes separately. Interestingly, the two groups presented very similar selection patterns (*SI Appendix*, Fig. S23). Strong, shared selection signals were detected on chromosomes 2, 11, 17, and 18. The regions on chromosomes 2 and 17 have been detected as sweep regions previously; they contain, respectively, the sex determination locus (30, 33, 39) and genes that may contribute to berry and seed size (28). The pattern of shared sweeps supported our demographic analyses indicating that wine grapes originated from table grapes, such that some important regions related to traits were under selection in all grapes during domestication. However, there were also multiple loci that were under selection separately in wine or table grapes. For example, one region on chromosome 3 had an extremely high PBS value in wine-ME2 comparisons, but not table-ME2 comparisons, suggesting selection specific to wine grapes.

**Fig. 3.**
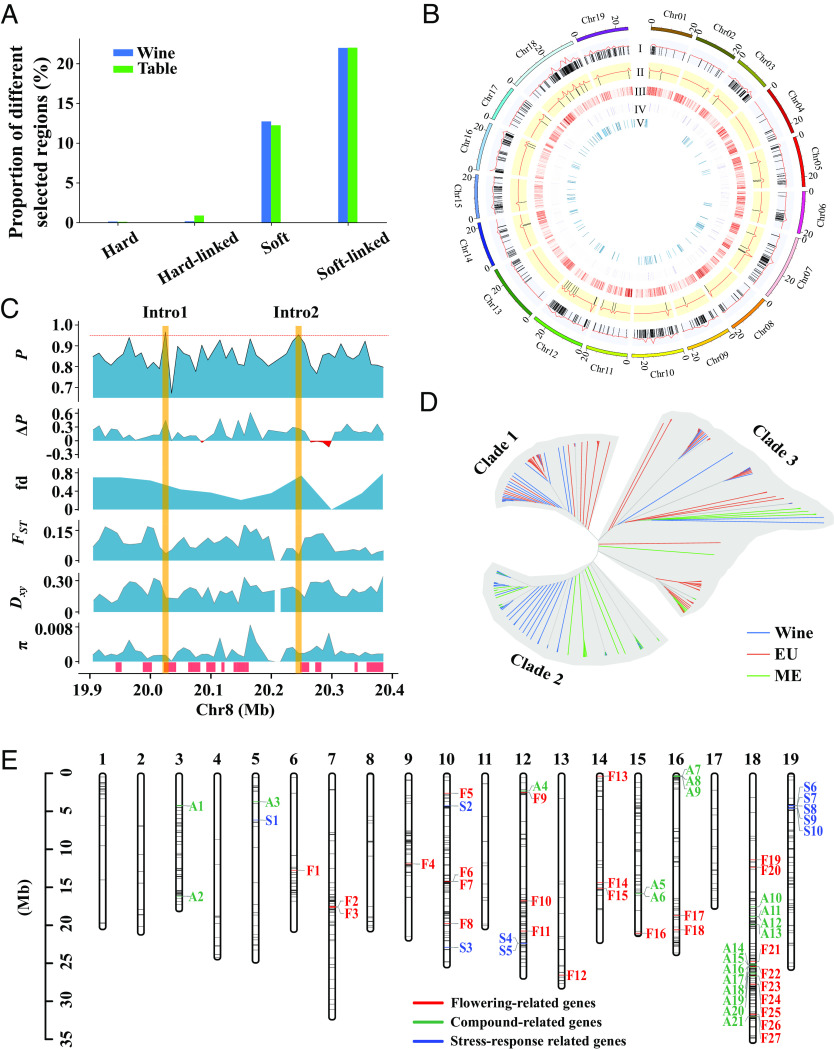
Machine-learning analyses of introgression and selective sweeps. (*A*) The outcome of selection analyses. (*B*) A circos plot of predicted introgressed and selected regions on each chromosome inferred from machine-learning applications. I, inferred introgressed regions from the EU to the wine group; II, introgressed regions from the wine to the EU group; III, regions predicted to have experienced soft sweeps in the wine group; IV, regions predicted to have experienced hard sweeps in the wine group; V, regions predicted to have experienced both introgression and selective sweeps in the wine group. (*C*) An example of regions that are inferred to have introgressed from the EU into wine grapes. *P* represents the probability of introgression; significant evidence for introgression was inferred when *P* > 95%. Two introgressed regions (Intro1 and Intro2) have been designated with a yellow background. *ΔP* is the probability of introgression from EU to wine groups minus the probability from wine to EU groups. *F_ST_D_xy_*, and π were counted every 10-kb window between wine and EU groups or within the wine group. fd is the same as that used in Fig. 2. The red boxes at the bottom of the figure show regions inferred to have been under selection. (*D*) The phylogenetic tree of the intro2 region. The blue lines represent wine grapes, the red lines represent EU grapes, and the green lines are ME grapes which included ME1 and ME2 grapes. (*E*) Three kinds of genes on introgressed regions. Putatively introgressed regions predicted by Filet are marked on the 19 chromosomes using black lines. The three colors represent genes inferred to be within introgressed regions with three different predicted functions: Red are flowering-related genes, green are aromatic compound-related genes, and blue are stress-response genes.

We also used machine learning methods to characterize selective sweep regions, using Shic (40, 41) (*Material and Methods*). We found that the most significant regions were classified as soft-linked selected regions—i.e., >21% of genome sequences in the wine and table group fell into this category (Fig. 3*A* and Datasets S1 and S2). The next common classification category was soft-selected regions, containing ~12% of genome sequences in the wine and table populations. Relatively, few regions were recognized as hard-selected or hard-linked selected regions (0.14% or 0.17% in wine grapes and 0.11% or 0.88% in table grapes, respectively). As expected, nucleotide diversity (π) of the wine population was much lower in these regions (Fig. 3*C*), consistent with selection removing genetic diversity. Selected regions also had high recombination rates: The average recombination rate (cM/kb) was 1.5 and 1.3 times higher than the genomic background in hard and soft selection regions, respectively (*SI Appendix*, Table S7).

To investigate the relationship between selection and introgression, we first sought to refine the boundaries of introgressed regions. Although we detected significant introgression signals with fd, it has substantial limitations: 1) It is difficult to define the threshold of fd to separate introgressed and nonintrogressed regions; 2) fd cannot distinguish the direction of introgression (e.g., from EU to wine or vice versa); 3) relatively large windows are necessary to apply the method as recommended (42). For example, with our data, only 67% of genomic regions were valid (i.e., containing 100 biallelic SNPs) when using 50-kb windows, and 80% were valid with 100-kb windows. To detect introgressed regions more precisely, we identified them using another machine learning–based classifier, Filet (43). We applied Filet with 22 separate parameters to fit the model and to compare EU to wine grapes (*SI Appendix*, Table S8). Filet identified a total of 836 regions (10 kb per region) as introgressed from EU *sylvestris* into wine grapes (Fig. 3*B*, *SI Appendix*, Fig. S24, and Dataset S3), representing 1.82% of the total genome. However, only 0.11% of the EU *sylvestris* genome was inferred to be introgressed from wine grapes, suggesting clear directionality to introgression events. The proportion of introgressed regions also varied among chromosomes; 4.38% of chromosome 18 was inferred to be introgressed from EU wild grapes, followed by chromosome 10 with 3.47%. In contrast, chromosome 2 had the lowest percentage of putatively introgressed regions (0.47%) (*SI Appendix*, Fig. S24).

Fig. 3*C* provides an example that illustrates the results. This diagram shows two regions on chromosome 8 that were inferred to be introgressed from EU to wine grapes, and it also includes information about selected regions in wine grapes. These two regions constitute 0.5 Mb in total, with 0.175 Mb (35%) overlapping putatively selected regions. As expected, *F_ST_* and *D_xy_* were low for these regions. To further validate the reliability of these predictions, we generated the local phylogenetic tree in one of the introgressed regions (Fig. 3*D*). This phylogeny included three clades: clade 1, which consisted of wine grapes and EU wild grapes; clade 2, which consisted of wine grapes and ME wild grapes; and clade 3, which consisted of wines grapes, EU wild grapes, and ME wild grapes. These results strongly implicate the introgression of this region from EU *sylvestris* into some wine grapes.

Altogether, our machine learning analyses identified putatively introgressed regions, putatively selected regions, and their overlaps. Out of 836 introgressed regions, 298 (or 36%) overlapped with predicted selected (hard, hard-linked, soft, and soft-linked) regions (Fig. 3*B*). Among the 298 overlapping regions, 176 introgressed regions were inferred to have been under hard or soft selection, as opposed to linked-selection. Across the genome, there were 292 genes in regions inferred to have been under selection and also putatively introgressed (*SI Appendix*, Fig. S25 and Dataset S4), including 57 such genes on chromosome 18. We investigated GO functions for the genome-wide set of introgressed-selected genes and focused on three kinds of genes (flowering-related genes, flavor-related genes, and stress response-related genes) (Fig. 3*E* and *SI Appendix*, Table S9). Interestingly, many flowering-related genes were selected after introgression, followed by aromatic compound-related genes. Most of the enrichment for GO categories was also related to flavor, including lignin catabolic processes, L-phenylalanine catabolic processes, and cinnamic acid biosynthetic processes (*SI Appendix*, Fig. S26). Thus, based on our analyses, adaptive introgression between wine and EU *sylvestris* appears to have primarily affected flavor-related traits.

### The Maladaptive Effects and Hitchhiking Genetic Load via Introgression.

**Fig. 4.**
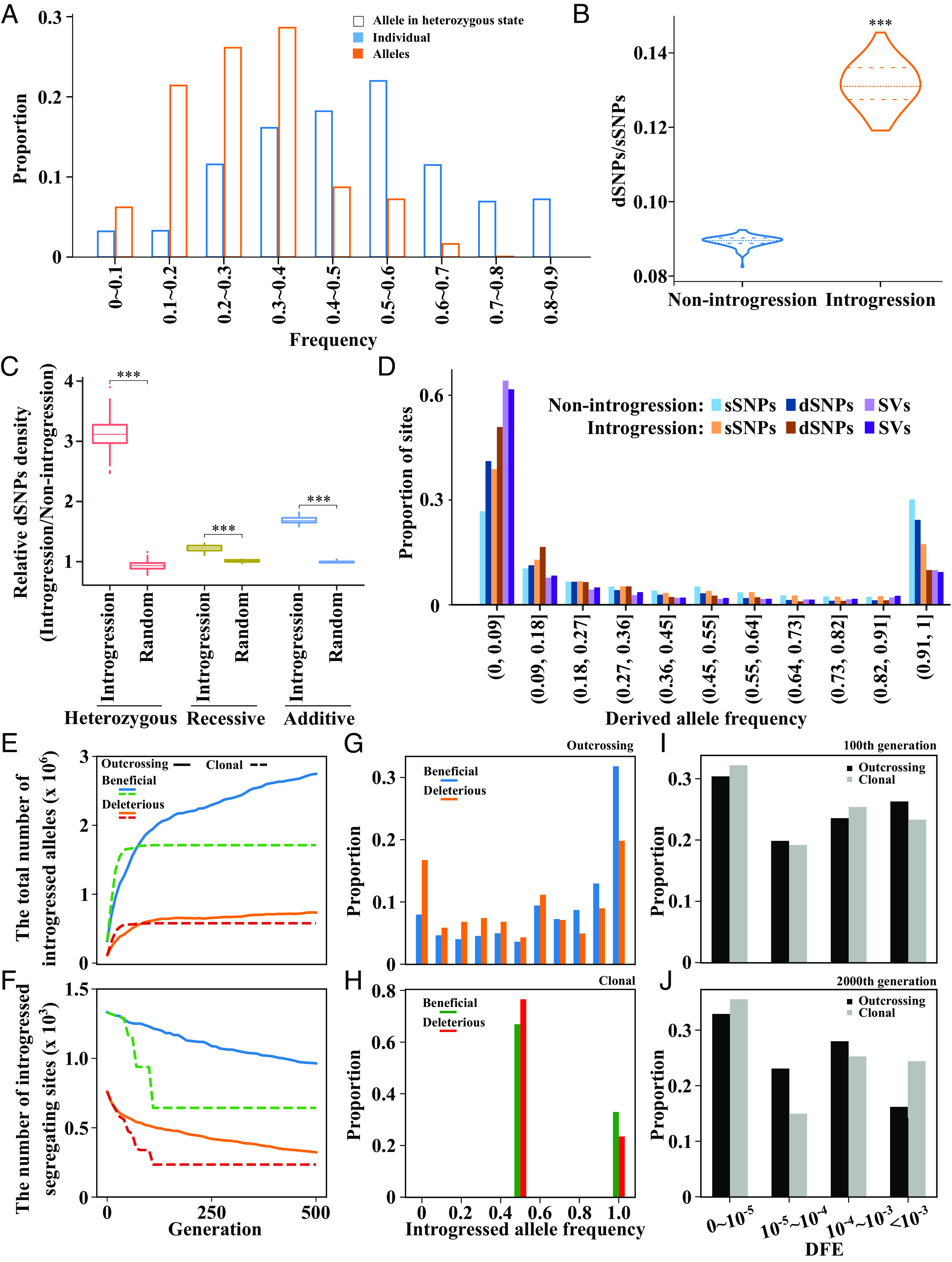
Features of variants in putatively introgressed regions of wine group and estimation of introgressed alleles by forward simulation. (*A*) Proportion of introgressed alleles in different frequencies in the wine group. The *x *axis shows different frequency bins. Blue bars indicate the frequency of individuals that contained the introgressed alleles; the orange bars indicate the frequency of introgressed alleles. The *y* axis is the proportion of the introgressed alleles that fall into each frequency bin. The shaded area in each bar indicates the average proportion of alleles in the heterozygous state. (*B*) Comparisons between the introgression region and nonintrogression region for the ratio of dSNPs to sSNPs per individual overall. Student’s *t* test (***P < 0.001) (*C*) The ratio of putatively deleterious SNP (dSNP) frequencies in introgressed regions relative to nonintrogressed regions. Heterozygous reflects dSNPs found in a heterozygous state. The recessive model considers only dSNPs in a homozygous state, and the additive model was calculated by using the number of heterozygous dSNPs plus twice the number of recessive dSNPs. The random values were used as negative control, which were calculated by selecting the regions randomly on the whole genome that had the same length as introgressed regions. Student’s *t* test (***P < 0.001) (*D*) The SFS of synonymous SNPs (sSNP), deleterious SNPs (dSNPs), and structural variants (SVs) in nonintrogression regions and introgression regions. (*E*–*J*) Analysis of the destiny of introgressed alleles by forward simulation. The total number of introgressed alleles in the whole introgressed population (*E*). The number of different kinds of introgressed alleles in whole introgressed population (*F*). The SFS of introgressed beneficial and deleterious alleles at the 500th generation after hybridization in the outcrossing group (*G*) and clonal group (*H*). The DFE distribution of introgressed alleles in outcrossing and clonal populations at the 100th generation (*I*) and the 2000th generation after hybridization (*J*).

To investigate the genetic consequence of introgression in wine grapes, we estimated allele frequencies in introgressed regions. Almost 48% of introgressed alleles were shared by more than half of the wine grape individuals, but typically in a heterozygous state, which led to only 9% of these alleles having frequencies >50% (Fig. 4*A* and *SI Appendix*, Fig. S27). Furthermore, we found that introgression affects deleterious load. The dSNPs/sSNPs ratio is significantly higher in the introgression region than the nonintrogression region (*P* < 0.05, Student’s *t* test, Fig. 4*B*). The density of dSNP was also significantly elevated in introgressed regions for all three types of loads (heterozygous, recessive, and additive) compared to nonintrogressed regions (*P* < 0.05, Student’s *t* test, Fig. 4*C*). In particular, the density of heterozygous load was ~threefold higher in introgressed regions than nonintrogressed regions (Fig. 4*C*). We attribute this last finding to clonal propagation because recessive or partially recessive dSNPs can “hide” indefinitely in a heterozygous state in clonal lineages (33), which means that wine grapes maintain two sets of deleterious mutations in introgressed regions: native dSNPs and dSNPs from EU. These two sets drive  higher dSNP densities in these regions. Overall, these calculations suggest that introgressed regions have increased the number of dSNPs in wine grapes, but the effects of these dSNPs may be hidden by their heterozygous state.

We also compared the site frequency spectra (SFS) of deleterious and structural variants (SVs) in introgressed and nonintrogressed regions, using synonymous SNPS (sSNPs) as a presumably neutral control. Compared to sSNPs, the dSNP and SV frequency spectra were significantly skewed toward the left, consistent with purifying selection in wine grapes (*P* < 0.05, Wilcoxon signed-rank test, Fig. 4*D*). Interestingly, dSNPs in introgressed regions also exhibited significantly leftward shifts of the SFS relative to dSNPs in the nonintrogressed regions, indicating a stronger purifying selection on introgressed regions (*P* < 0.05, Wilcoxon signed-rank test, Fig. 4*D*). The slightly elevated proportion of moderate frequency (i.e., <~0.3, Fig. 4*D*) dSNPs and SVs in introgressed regions, relative to nonintrogressed regions, again suggests that introgressed regions tend to be widespread but found in a heterozygous and potentially hidden state.

### Simulations and the Dynamics of Introgressed Alleles.

In order to understand the dynamics of introgressed alleles, we conducted forward simulations to investigate introgression from outcrossing populations into clonal and outcrossing populations. We simulated hybridization with both beneficial and deleterious alleles, followed by 10 generations of outbreeding before transitioning to clonality (*Material and Methods*). We found that the total number of introgressed alleles, both beneficial and deleterious, increased after hybridization until they reached an equilibrium (Fig. 4*E* and *SI Appendix*, Fig. S28). There was nonetheless an important difference between clonal and outcrossing populations because clonal populations reached equilibrium much more quickly (i.e., in ~100 instead of ~2,000 generations), generally suggesting that clonal populations may be able to capture introgressed alleles more effectively in the short term. We also examined the number of introgressed segregating sites over time, which declined after introgression before reaching an equilibrium. The number of introgressed segregating sites was lower for deleterious than for beneficial alleles at equilibrium, suggesting the effects of positive and negative selection (Fig. 4*F* and *SI Appendix*, Fig. S29). This decline suggested that some purging and loss of introgressed sites occurred following introgression, but the remaining sites were fixed throughout the population eventually (Fig. 4 *G* and *H*, *SI Appendix*, Figs. S30 and S31, and Movies S1 and S2). Interestingly, in the clonal population, the frequency of most alleles was maintained at 50% at equilibrium, reflecting maintenance of the heterozygous state under our simulation conditions (Fig. 4 *G* and *H* and Movies S1 and S2). We also estimated the distribution of fitness effects (DFE) of introgressed alleles. Purifying selection was stronger in clonal than outcrossing populations within 100 generations after admixture (Fig. 4*I*, *SI Appendix*, Fig. S32, and Movie S3), but the strength of selection continued to increase for the outcrossing population to be more prominent at generation 2000 (Fig. 4*J*, *SI Appendix*, Fig. S33, and Movie S3). To sum, these simulations illustrate that the fate of introgressed alleles can differ dramatically between clonal and outcrossing systems. The accelerated dynamics in clonal populations generally suggest that introgression may be a historically important process for improving clonal systems like domesticated grapes.

### Introgression Affects Supergenes.

**Fig. 5.**
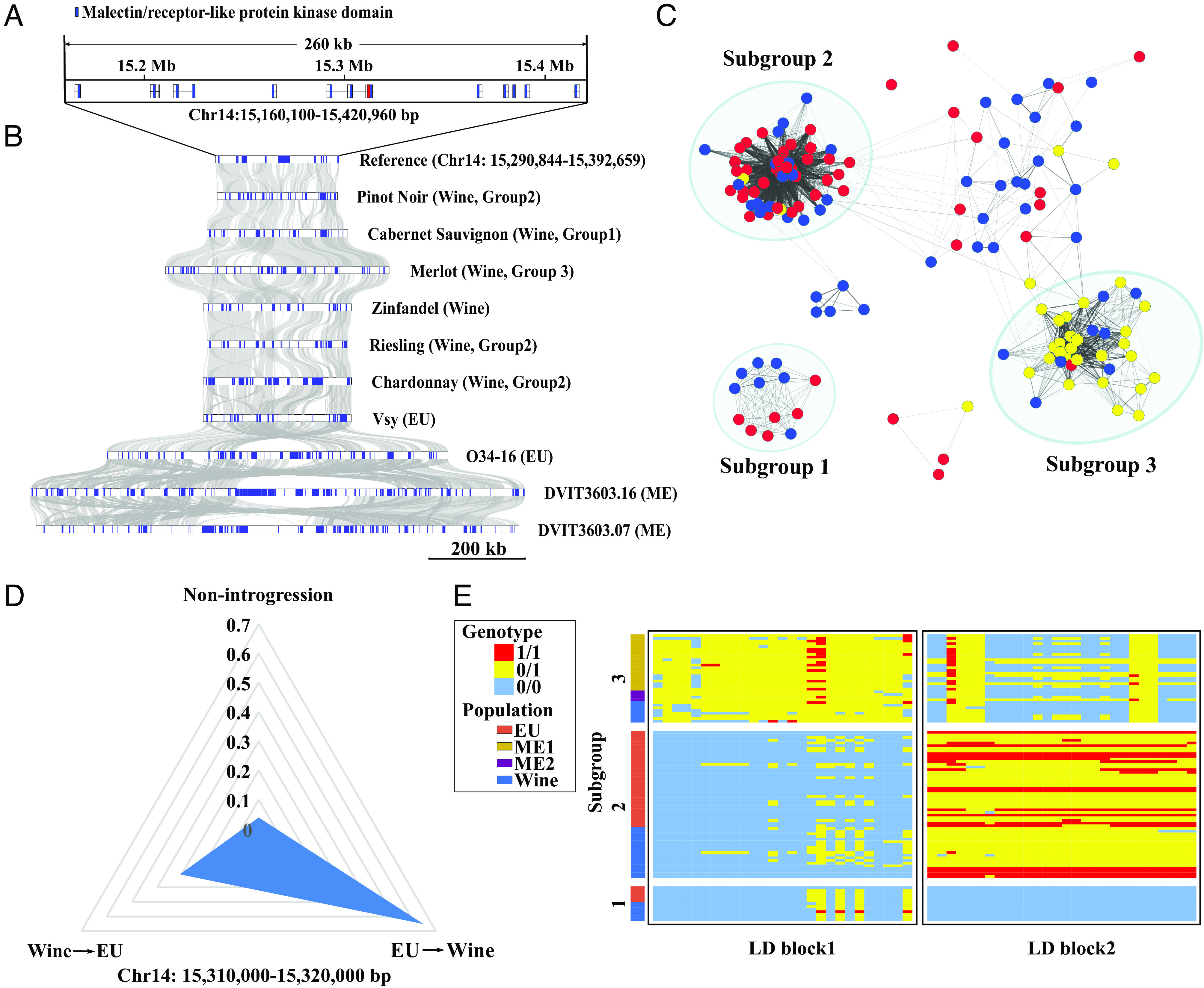
A region containing a *Fer-like* gene was predicted to have introgressed from the EU and under selection. (*A*) A cluster of the malectin/receptor-like protein kinase domain was observed around the introgressed region. The malectin domain is represented by blue boxes. The red boxes indicate the CDS of the candidate *Fer-like* gene. (*B*) Syntenic relationship of the cluster region in the *vinifera* species. The blue boxes indicate the malectin/receptor-like protein kinase domain. (*C*) Kinship analysis of grapes in this region. The kinship value between 0.5 and 1.5 is shown using gray solid lines. The wine grapes are shown by blue dots, the EU grapes are shown by red dots, and the ME grapes are shown by yellow dots. (*D*) Introgression probability of this region by Filet. (*E*) The heatmap of genotypes of two main LD blocks in this region. Note that 0/0 is indicated by blue, 0/1 is indicated by yellow, and 1/1 is indicated by red.

Finally, we investigated the inferred sizes of introgressed regions after merging Shic-inferred introgressed regions that were separated by <50 kb (as determined by LD decay) (*SI Appendix*, Figs. S34 and S35). After this merging, we obtained a total of 532 windows, ranging from 10 to 250 kb. However, 81.6% of putatively introgressed regions were <40 kb in length even after merging. Only 25 of 532 windows were >100 kb, with the largest being ~250 kb (*SI Appendix*, Table S10). We focused on these larger (>100 kb) regions to assess sets of potentially cointrogressed genes. For example, one 120-kb region on chromosome 14 contained a gene cluster. To better understand the history and genetic architecture of this region, we compared 11 assembled genomes consisting of six wine grapes, two EU wild grapes, and two ME wild grapes. Synteny across this region indicated that part of the region was duplicated in the ME grapes and in one EU grape, representing a region of 260 kb containing 11 genes with the malectin/receptor-like protein kinase domain. In contrast, most wine grapes (except Merlot) lacked all or part of the duplication (Fig. 5 *A* and *B*). This result suggests that EU wild grapes initially lost the duplication of this cluster, and this allele was then introgressed into wine grapes from EU grapes.

We further examined the genes in this region by blasting them to the *Arabidopsis* protein database. One gene in this region was identified as homologous to the *Feronia (Fer)* gene in *Arabidopsis*. *Fer* affects male–female gametophyte interactions during pollen tube reception, but it also influences cell growth as well as biotic and abiotic stress response in *Arabidopsis* and rice (44–48). We propose that this *Fer-like* gene was differentiated in EU and ME wild grapes and that some wine grapes obtained their *Fer* alleles from EU wild grapes. Consistent with this conjecture, the KINSHIP correlation of the 10-kb window that contained this gene separated the grapes into three subgroups (coefficients 0.5 to 1.5 were shown), with two subgroups clustering EU grapes with many wine grapes (Fig. 5*C*). Both the *D_xy_* and *F_ST_* showed low genetic divergence between the wine and EU in subgroup 1 and subgroup 2, supporting the evidence of introgression detected by Filet analysis (*SI Appendix*, Fig. S36).

Although there was a high probability (65%) of introgression from the EU to the wine population for this region, introgressed alleles were not fixed across wine accessions (Fig. 5*D*). The retention of introgression events and the dynamics of selection on introgressed regions are likely to be a function of recombination dynamics. To investigate the recombination events of this locus and the flanking regions, we estimated the two major haplotypes (representing 44% of variants) using LDna, a method that identified the clusters of loci in high LD from population genomic datasets using network analysis (49). We found an identical LD block1 (ancient homozygosity) prevalent in EU and wine grapes (Fig. 5*E*). There was a different LD block (LDblock2) in a homozygous state within group 1 and heterozygous state within group 2, as well as derived homozygous haplotypes were observed within group 2. These LD analyses suggest that recombination did not break up this region in wine grapes after introgression from EU wild grapes.

## Discussion

As a source of both table fruit and wine, domesticated *V. vinifera* is the most valuable horticultural crop in the world (25). It has also rapidly become a model system for the study of the dynamics of perennial domestication (16), the evolution of mating systems (39), and the evolutionary genetic consequences of clonality and genomic breeding (30, 33). Accordingly, previous population genomic analyses have tackled important issues in grapevine genomic diversity ranging from the timing and number of domestication events, their demographic history (27, 30), selective sweeps (26), the accumulation of SVs in clonal lineages (33), and introgression between wine grapes and European *sylvestris* (28).

This study has been designed to categorize introgressed regions and the evolutionary processes that have affected these regions. We have focused on introgression because it is not only important for current breeding efforts of important crops (50) but also because historical introgression has been a major force for shaping genetic diversity in crops like rice (51), olives (52), apples (53, 54), and maize (18, 55, 56). However, the genomic extent of introgression has not been thoroughly characterized in any crop, nor has there been a careful accounting for the adaptive forces that drive successful introgression events. There are at least two adaptive scenarios that likely apply to the retention of introgressed regions. The first is that introgression contributes to adaptive alleles affecting agronomic traits. For example, introgression has contributed to highland adaptation in maize (56), stress tolerance in potatoes (57), and perhaps fruit quality in apples (53). While these isolated examples prove the point, we must accentuate again that the potential phenotypic bases for adaptative introgression events are generally not well characterized for any crop. A second, nonexclusive adaptive explanation is that introgression drives increased fitness because it introduces genomic segments that reduce the deleterious burden—i.e., reversing the so-called cost of domestication (58). There is at least one known instance where a genomic region with fewer deleterious variants introgressed into a crop from wild populations (55), suggesting that this mechanism could be common.

The opportunity for adaptive introgression likely occurs when a crop disperses from its center of domestication and contacts new wild populations or species (36, 59). The migration of grapevines into western Europe provides an ideal system for study, for two reasons. First, the migration to Europe was not particularly recent because the domesticated grapevine was spread to Europe around 3,000 years ago (25, 31). In fact, some important current cultivars were already being grown by Romans > 1,000 years ago (25, 31, 34). The point is that there has been enough time for introgression with European *sylvestris* to have occurred. Second, European *sylvestris* populations (EU) are distinct, in terms of both their divergence from other *sylvestris* populations and their population histories (Figs. 1*B* and 2 *A* and *B* and *SI Appendix*, Figs. S2–S5). The EU group has lower levels of nucleotide diversity than either the ME1 or ME2 groups (Fig. 2*A* and *SI Appendix*, Fig. S10), suggesting lower historical population sizes. The genetic divergence of EU populations provides an opportunity to detect introgression events.

Although a genomic signal of introgression had been detected between EU and wine grapes previously (28, 29), there has not yet been an intensive study to detect regions of introgression and to infer the forces that contribute to their establishment. Many methods have been developed to detect introgression, including probability models (60), clustering (61), genomic statistical indexes (62, 63), and ancestral history reconstruction (64). It can be difficult to identify these regions accurately since ILS and introgression can generate similar patterns of genetic sharing on candidate regions (9, 65). Some methods have been designed to exclude the effect of ILS, such as the ABBA-BABA test and derived methods (66, 67), but these methods have some limitations that include sensitivity to effective population size and difficulty locating regions accurately (42). In this study, we have utilized ABBA-BABA statistics (e.g., fb, fd, and fdM) but also implemented complementary methods based on machine learning. Machine learning approaches combine summary statistics to achieve sensitivity and accuracy that often exceeds inference based on a single summary statistic (43, 68). Population genetic methods based on machine learning have been applied successfully in human genetics and for crop breeding and trait prediction (69, 70). However, to our knowledge, it has not been used to study processes associated with plant domestication.

Altogether, we identified 836 introgressed segments (based on 10-kb windows) from the EU to the wine group, with only 47 segments resulting from introgression in the opposite direction (i.e., from the wine group to the EU group) (Fig. 3*B* and *SI Appendix*, Fig. S24). There is thus ample evidence for introgression from EU *sylvestris* to wine grapes but surprisingly little corresponding evidence of introgression between EU and table grapes (Fig. 2 *C* and *D* and *SI Appendix*, Figs. S21 and S22). Given introgression between EU *sylvestris* and wine grapes, an important question is whether it has had an adaptive basis. Adaptive introgression has played an important role in crop domestication, such as in maize, barley, potato, and rice (71). Our analyses provide general evidence to suggest that introgression has contributed to key agronomic traits of wine grapes and that the introgression was adaptive, based on overlaps with selective sweeps. We found that the genes within introgressed regions were enriched for biological processes that could be interpreted to be related to aromatic qualities, a trait of great importance to wine grapes, including lignin catabolic processes, L-phenylalanine catabolic processes, and cinnamic acid biosynthetic processes, and some processes related to flowering. We were surprised, however, that we did not see enrichment for disease-defense genes because these have been commonly exchanged between wild *Vitis* species (38) and are often associated with local adaptation. The lack of exchange of disease-resistance genes may reflect that many of the major, modern diseases of grapes come from America and were likely introduced long after the hypothesized hybridization events. We note, however, that defense response genes were enriched in the 5% fd regions in table grapes but not wine grapes. The enrichment of phenylpropanoid metabolic, secondary metabolic, and aromatic compounds in wine grapes (Fig. 2*G*), indicates that EU wild grapes have served chiefly as sources for flavor-related traits related to wine-making.

We also sought to establish the adaptive nature of introgression events by characterizing signatures of selective sweeps within introgressed regions of wine grapes. We recognize that this exercise probably has low statistical power, both because our analyses suggest that most introgressed variants are in low frequency and because it is difficult to detect ongoing sweeps based on low-frequency variants. Despite this obstacle, we identified a total of 298 windows out of 836 introgressed windows (10 kb per window) under selective sweeps (hard, hard-linked, soft, or soft-linked selection), representing a substantial enrichment.

We also investigated the timing and intensity of gene flow by constructing and analyzing 34 models with different timing and direction of introgression events. Our best-fit model suggests that introgression has been ongoing for the past ~1.8 thousand years, corresponding roughly to the timing of the dispersal of grapes to Europe, especially from EU wild grapes to wine grapes. These models also infer that gene flow from EU *sylvestris* to table grapes has been relatively low—i.e., roughly 100 times less intense than gene flow to wine grapes. This result is further supported by fd analysis and chloroplast and mitochondria phylogenetic trees (Fig. 2*E* and *SI Appendix*, Figs. S6, S7, and S21), where ~30% of the organelle haplotypes of wine grapes clustered with EU *sylvestris*, but few table grapes (~10%) clustered with EU *sylvestris*.

The question remains as to whether introgressed regions provide an adaptive benefit by reducing deleterious load. This is a complex question. Since EU *sylvestris* has lower nucleotide diversity than other groups, one predicts that genetic drift may lead to a higher deleterious load in EU populations. In accordance with this expectation, we find that introgressed regions in wine grapes generally have higher numbers of putatively deleterious mutations than the genomic background. On average, the frequency of heterozygous harmful SNPs in introgressed regions is three times that of the randomly selected regions of the same length (Fig. 4*C*). Their frequency distribution is also skewed in introgressed regions because > 50% of the deleterious SNPs (dSNPs) in the introgressed regions were found at frequencies <9%. In the nonintrogressed regions, by contrast, ~40% of the dSNPs had frequencies <9% (Fig. 4*D*). Nonetheless, if deleterious mutations are recessive, they will not provide a fitness detriment in the heterozygous state (13). Consistent with this idea, deleterious regions had especially high heterozygosity.

Overall, these observations provide a multifaceted view of the forces that have driven introgression in this system. On the one hand, evidence suggests that introgression has contributed to agronomic traits but introduced deleterious mutations. However, this combination likely contributes to a short-term gain of fitness via heterozygosity, adding to previous observations that clonal lineages may hide deleterious mutations in the heterozygous state (72). In this study, we have inferred this similar dynamic process, finding that clonal propagation can quickly select favorable and detrimental introgressed alleles in a much shorter time (< 100 generations) than for outcrossing population (>2,000 generations) (*SI Appendix*, Figs. S28–S31 and Movies S1 and S2). Interestingly, most beneficial introgressed alleles are fixed in an outcrossing population eventually but not in clonal population (Movies S1 and S2). We conclude that introgression has contributed to the complement of the beneficial and deleterious variants in the heterozygous state within grapes. These beneficial and deleterious variants may prove to be major targets for genomic design of grapevine breeding (73), including the purging of putatively deleterious variants that may become uncovered during the process of sexual breeding.

## Material and Methods

For full materials and methods, see *SI Appendix*. A total of 40 samples were sampled from the USDA grape germplasm collections in Davis, California (*SI Appendix*, Table S1). Genomic DNA was isolated from leaf samples, paired-end sequencing libraries were constructed, and libraries were sequenced using the Illumina HiSeq 4000 platform with 150-bp paired reads to a target coverage of 30x. The raw sequencing data have been deposited in the Short Read Archive at NCBI under BioProject ID: PRJNA910315 (74) and the National Genomics Data Center (NGDC) Genome Sequence Archive with BioProject number: PRJCA016655 (75). We also used Illumina raw reads of 305 samples from previous publications that were downloaded from the Short Read Archive at NCBI. For all resequencing data, reads were trimmed, filtered, and mapped to the *V. vinif*era reference genome (33). The GATK4 was used for SNP and genotype calling. Vcftools was then used to perform filtering to reduce false positives (76) (*SI Appendix*, *Supplementary Methods*).

Phylogenetic trees were constructed using SNP data by iqtree with 1,000 bootstrap replicates (77). Vcftools was used to calculate the summary statistics of heterozygous sites and nucleotide diversity ( π ). The heterozygosity of each sample was calculated by dividing the number of heterozygous sites in each sample by the number of all SNPs. IBD was calculated using plink with parameter: --genome. Sequence similarity (*D_xy_*), and fixation indices (*F_ST_*) were calculated using the Python script: popgenWindows.py (https://github.com/simonhmartin/genomics_general) with 50-kb nonoverlapping windows.

The continuous-time sequential Markovian coalescent approximation implemented fastsimcoal (version: fsc27) was used to further estimate the population history under complex evolutionary scenarios (78, 79). We used Dsuite (https://github.com/millanek/Dsuite) to calculate Patterson's D (ABBA-BABA) and f4-ratio statistics across populations to evaluate introgression probabilities across the genome (37). We also used the machine learning–based introgression analysis program Filet to predict introgressed regions (43).

The forward simulation software SLiM3 (80) was used to evaluate the heterozygosity of crops and the dynamics of introgressed alleles under different propagation types: outcrossing or cloning. Additional details are available in *SI Appendix*.

## Supplementary Material

Appendix 01 (PDF)

Dataset S01 (XLSX)

Dataset S02 (XLSX)

Dataset S03 (XLSX)

Dataset S04 (XLSX)

Movie S1. The SFS change of introgressed alleles after hybridization in outcrossing population. The bar located directly above the movie indicates the generations after hybridization.

Movie S2. The SFS change of introgressed alleles after hybridization in clonal population. The bar located directly above the movie indicates the generations after hybridization

Movie S3. The DFE change of introgressed alleles after hybridization. The bar located directly above the movie indicates the generations after hybridization.

## Data Availability

Data (genome sequences; script) have been deposited in NCBI, NGDC and GitHub (PRJNA910315 (74); PRJCA016655 (75); https://github.com/zhouyflab/Grapevine_Adaptive_Maladaptive_Introgression (81)).

## References

[1] L. H. Rieseberg, F. J. Wendel, Hybrid Zones & The Evolutionary Process, Chapter 4, R. G. Harrison, Ed. (Botany Publication and Papers, 1993), pp. 70–109.

[2] S. M. Aguillon, T. O. Dodge, G. A. Preising, M. Schumer, Introgression. Curr. Biol. **32**, R865–R868 (2022). 35998591 10.1016/j.cub.2022.07.004 PMC10581619

[3] N. B. Edelman, J. Mallet, Prevalence and adaptive impact of introgression. Annu. Rev. Genet. **55**, 265–283 (2021). 34579539 10.1146/annurev-genet-021821-020805

[4] L. H. Rieseberg, S. J. Brunsfeld, Molecular Systematics of Plants, P. S. Soltis, D. E. Soltis, J. J. Doyle, Eds. (Springer US, Boston, MA, 1992), pp. 151–176.

[5] T. Nagylaki, Y. Lou, Tutorials in Mathematical Biosciences IV: Evolution and Ecology, A. Friedman Ed. (Springer Berlin Heidelberg, Berlin, Heidelberg, 2008), pp. 117–170.

[6] S. Yeaman, M. C. Whitlock, The genetic architecture of adaptation under migration-selection balance. Evol. Int. J. Organic Evol. **65**, 1897–1911 (2011). 10.1111/j.1558-5646.2011.01269.x 21729046

[7] C. Veller, N. B. Edelman, P. Muralidhar, M. A. Nowak, Recombination and selection against introgressed Dna. Evolution **77**, 1131–1144 (2023). 36775972 10.1093/evolut/qpad021

[8] J. Ågrena, C. G. Oakley, J. K. Mckay, J. T. Lovell, D. W. Schemske, Genetic mapping of adaptation reveals fitness tradeoffs in Arabidopsis Thaliana. Proc. Natl. Acad. Sci. U.S.A. **110**, 21077–21082 (2013). 24324156 10.1073/pnas.1316773110 PMC3876199

[9] Y. Zhou , Importance of incomplete lineage sorting and introgression in the origin of shared genetic variation between two closely related pines with overlapping distributions. Heredity **118**, 211–220 (2017). 27649619 10.1038/hdy.2016.72 PMC5315522

[10] O. Savolainen, M. Lascoux, J. Merilä, Ecological genomics of local adaptation. Nat. Rev. Genet. **14**, 807–820 (2013). 24136507 10.1038/nrg3522

[11] M. Schumer , Natural selection interacts with recombination to shape the evolution of hybrid genomes. Science. **360**, 656–660 (2018). 29674434 10.1126/science.aar3684 PMC6069607

[12] E. M. Oziolor , Adaptive introgression enables evolutionary rescue from extreme environmental pollution. Science **364**, 455–457 (2019). 31048485 10.1126/science.aav4155

[13] K. Harris, R. Nielsen, The genetic cost of neanderthal introgression. Genetics **203**, 881–891 (2016). 27038113 10.1534/genetics.116.186890 PMC4896200

[14] V. Le Corre, M. Siol, Y. Vigouroux, M. I. Tenaillon, C. Délye, Adaptive introgression from maize has facilitated the establishment of teosinte as a noxious weed in Europe. Proc. Natl. Acad. Sci. U.S.A. **117**, 25618–25627 (2020). 32989136 10.1073/pnas.2006633117 PMC7568241

[15] H. Hilton, B. S. Gaut, Speciation and domestication in maize and its wild relatives: Evidence from the globulin-1 gene. Genetics **150**, 863–872 (1998). 9755214 10.1093/genetics/150.2.863 PMC1460357

[16] Y. Zhou, A. Muyle, B. S. Gaut, The Grape Genome, D. Cantu, M. A. Walker, Eds. (Springer International Publishing, Cham, 2019), pp. 39–55.

[17] Y. Zhou, L. Zhang, J. Liu, G. Wu, O. Savolainen, Climatic adaptation and ecological divergence between two closely related pine species in Southeast China. Mol. Ecol. **23**, 3504–3522 (2014). 24935279 10.1111/mec.12830

[18] T. Lin , Genomic analyses provide insights into the history of tomato breeding. Nat. Genet. **46**, 1220–1226 (2014). 25305757 10.1038/ng.3117

[19] A. Jarvis, A. Lane, R. J. Hijmans, The effect of climate change on crop wild relatives. Agric. Ecosyst. Environ. **126**, 13–23 (2008).

[20] G. Zhu , Rewiring of the fruit metabolome in tomato breeding. Cell **172**, 249–261.e12 (2018). 29328914 10.1016/j.cell.2017.12.019

[21] Y. Liu , Genomic basis of geographical adaptation to soil nitrogen in rice. Nature **590**, 600–605 (2021). 33408412 10.1038/s41586-020-03091-w

[22] S. Walkowiak , Multiple wheat genomes reveal global variation in modern breeding. Nature **588**, 277–283 (2020). 33239791 10.1038/s41586-020-2961-x PMC7759465

[23] P. Mcgovern , Early neolithic wine of Georgia in the South Caucasus. Proc. Natl. Acad. Sci. U.S.A. **114**, E10309–E10318 (2017). 29133421 10.1073/pnas.1714728114 PMC5715782

[24] F. Mercati , Integrated bayesian approaches shed light on the dissemination routes of the Eurasian Grapevine Germplasm. Front. Plant Sci. **12**, 692661 (2021). 34434204 10.3389/fpls.2021.692661 PMC8381769

[25] S. Myles , Genetic structure and domestication history of the grape. Proc. Natl. Acad. Sci. U.S.A. **108**, 3530–3535 (2011). 21245334 10.1073/pnas.1009363108 PMC3048109

[26] Z. Liang , Whole-genome resequencing of 472 vitis accessions for grapevine diversity and demographic history analyses. Nat. Commun. **10**, 1190 (2019). 30867414 10.1038/s41467-019-09135-8 PMC6416300

[27] Y. Dong , Dual domestications and origin of traits in grapevine evolution. Science. **379**, 892–901 (2023). 36862793 10.1126/science.add8655

[28] G. Magris , The genomes of 204 vitis vinifera accessions reveal the origin of European Wine Grapes. Nat. Commun. **12**, 7240 (2021). 34934047 10.1038/s41467-021-27487-y PMC8692429

[29] S. Freitas , Pervasive hybridization with local wild relatives In Western European grapevine varieties. Sci. Adv. **7**, eabi8584 (2021). 34797710 10.1126/sciadv.abi8584 PMC8604406

[30] Y. Zhou, M. Massonnet, J. S. Sanjak, D. Cantu, B. S. Gaut, Evolutionary genomics of grape (*Vitis vinifera* ssp. *vinifera*) domestication. Proc. Natl. Acad. Sci. U.S.A. **114**, 11715–11720 (2017). 29042518 10.1073/pnas.1709257114 PMC5676911

[31] J. Ramos-Madrigal , Palaeogenomic insights into the origins of french grapevine diversity. Nat. Plants **5**, 595–603 (2019). 31182840 10.1038/s41477-019-0437-5

[32] D. Cantu, M. A. Walker, Eds., The Grape Genome (Springer International Publishing, Cham, 2019).

[33] Y. Zhou , The population genetics of structural variants in grapevine domestication. Nat. Plants **5**, 965–979 (2019). 31506640 10.1038/s41477-019-0507-8

[34] F. Grassi, L. G. de, Back to the origins: Background and perspectives of grapevine domestication. Int. J. Mol. Sci. **22**, 4518 (2021). 33926017 10.3390/ijms22094518 PMC8123694

[35] A. Sivan , Genomic evidence supports an independent history of Levantine and Eurasian grapevines. Plants, People, Planet **3**, 414–427 (2021).

[36] B. S. Gaut, D. K. Seymour, Q. Liu, Y. Zhou, Demography and its effects on genomic variation in crop domestication. Nat. Plants **4**, 512–520 (2018). 30061748 10.1038/s41477-018-0210-1

[37] M. Malinsky, M. Matschiner, H. Svardal, Dsuite–Fast D-Statistics and related admixture evidence from VCF files. Mol. Ecol. Res. **21**, 584–595 (2021). 10.1111/1755-0998.13265 PMC7116594 33012121

[38] A. Morales-Cruz , Introgression among north american wild grapes (Vitis) fuels biotic and abiotic adaptation. Genome Biol. **22**, 254 (2021). 34479604 10.1186/s13059-021-02467-z PMC8414701

[39] M. Massonnet , The genetic basis of sex determination in grapes. Nat. Commun. **11**, 2902 (2020). 32518223 10.1038/s41467-020-16700-z PMC7283251

[40] A. D. Kern, D. R. Schrider, diploS/HIC: An updated approach to classifying selective sweeps. G3 (Bethesda). **8**, 1959–1970 (2018). 29626082 10.1534/g3.118.200262 PMC5982824

[41] D. R. Schrider, A. D. Kern, S/HIC: Robust identification of soft and hard sweeps using machine learning. PLoS Genet. **12**, e1005928 (2016). 26977894 10.1371/journal.pgen.1005928 PMC4792382

[42] S. H. Martin, J. W. Davey, C. D. Jiggins, Evaluating the use of Abba-Baba statistics to locate introgressed loci. Mol. Biol. Evol. **32**, 244–257 (2015). 25246699 10.1093/molbev/msu269 PMC4271521

[43] D. R. Schrider, J. Ayroles, D. R. Matute, A. D. Kern, Supervised machine learning reveals introgressed loci in the genomes of Drosophila simulans and D. sechellia. PLoS Genet. **14**, e1007341 (2018). 29684059 10.1371/journal.pgen.1007341 PMC5933812

[44] Y. Song , Feronia restricts pseudomonas in the rhizosphere microbiome via regulation of reactive oxygen species. Nat. Plants **7**, 644–654 (2021). 33972713 10.1038/s41477-021-00914-0

[45] P. Wang , Integrated omics reveal novel functions and underlying mechanisms of the receptor kinase feronia in Arabidopsis Thaliana. Plant Cell **34**, 2594–2614 (2022). 35435236 10.1093/plcell/koac111 PMC9252503

[46] H. Yang , Malectin/malectin-like domain-containing proteins: A repertoire of cell surface molecules with broad functional potential. Cell Surf. **7**, 100056 (2021). 34308005 10.1016/j.tcsw.2021.100056 PMC8287233

[47] Z. Yang , Mutations of two FERONIA-like receptor genes enhance rice blast resistance without growth penalty. J. Exp. Botany **71**, 2112–2126 (2020). 31986202 10.1093/jxb/erz541 PMC7242082

[48] S. Zhong , RALF peptide signaling controls the polytubey block in Arabidopsis. Science. **375**, 290–296 (2022). 35050671 10.1126/science.abl4683 PMC9040003

[49] Z. Li, P. Kemppainen, P. Rastas, J. Merilä, Linkage disequilibrium clustering-based approach for association mapping with tightly linked genomewide data. Mol. Ecol. Res. **18**, 809–824 (2018). 10.1111/1755-0998.12893 29673105

[50] C. Burgarella , Adaptive introgression: An untapped evolutionary mechanism for crop adaptation. Front. Plant Sci. **10**, 4 (2019). 30774638 10.3389/fpls.2019.00004 PMC6367218

[51] H. Wang, F. G. Vieira, J. E. Crawford, C. Chu, R. Nielsen, Asian wild rice is a hybrid swarm with extensive gene flow and feralization from domesticated rice. Genome Res. **27**, 1029–1038 (2017). 28385712 10.1101/gr.204800.116 PMC5453317

[52] C. M. Diez , Olive domestication and diversification in the mediterranean basin. New Phytol. **206**, 436–447 (2015). 25420413 10.1111/nph.13181

[53] N. Duan , Genome re-sequencing reveals the history of apple and supports a two-stage model for fruit enlargement. Nat. Commun. **8**, 249 (2017). 28811498 10.1038/s41467-017-00336-7 PMC5557836

[54] A. Cornille , New insight into the history of domesticated apple: Secondary contribution of the European wild apple to the genome of cultivated varieties. PLos Genet. **8**, e1002703 (2012). 22589740 10.1371/journal.pgen.1002703 PMC3349737

[55] L. Wang , The interplay of demography and selection during maize domestication and expansion. Genome Biol. **18**, 215 (2017). 29132403 10.1186/s13059-017-1346-4 PMC5683586

[56] M. B. Hufford , The genomic signature of crop-wild introgression in maize. PLoS Genet. **9**, e1003477 (2013). 23671421 10.1371/journal.pgen.1003477 PMC3649989

[57] M. A. Hardigan , Genome diversity of tuber-bearing solanum uncovers complex evolutionary history and targets of domestication in the cultivated potato. Proc. Natl. Acad. Sci. U.S.A. **114**, E9999–E10008 (2017). 29087343 10.1073/pnas.1714380114 PMC5699086

[58] J. Lu , The accumulation of deleterious mutations in rice genomes: A hypothesis on the cost of domestication. Trends Genet. **22**, 126–131 (2006). 16443304 10.1016/j.tig.2006.01.004

[59] R. S. Meyer, M. D. Purugganan, Evolution of crop species: Genetics of domestication and diversification. Nat. Rev. Genet. **14**, 840–852 (2013). 24240513 10.1038/nrg3605

[60] D. J. Lawson, G. Hellenthal, S. Myers, D. Falush, Inference of population structure using dense haplotype data. PLoS Genet. **8**, e1002453 (2012). 22291602 10.1371/journal.pgen.1002453 PMC3266881

[61] A. l. Price, , Sensitive detection of chromosomal segments of distinct ancestry in admixed populations. PLoS Genet. **5**, e1000519 (2009). 19543370 10.1371/journal.pgen.1000519 PMC2689842

[62] M. Nei, W. H. Li, Mathematical model for studying genetic variation in terms of restriction endonucleases. Proc. Natl. Acad. Sci. U.S.A. **76**, 5269–5273 (1979). 291943 10.1073/pnas.76.10.5269 PMC413122

[63] S. Wright, The genetical structure of populations. Ann. Eugen. **15**, 323–354 (1951). 24540312 10.1111/j.1469-1809.1949.tb02451.x

[64] S. Joly, P. A. Mclenachan, P. J. Lockhart, A statistical approach for distinguishing hybridization and incomplete lineage sorting. Am. Nat. **174**, E54–E70 (2009). 19519219 10.1086/600082

[65] J. A. Coyne, H. A. Orr, Patterns of speciation in Drosophila. Evol. Int. J. Organic Evol. **43**, 362–381 (1989). 10.1111/j.1558-5646.1989.tb04233.x 28568554

[66] R. E. Green , A draft sequence of the neandertal genome. Science. **328**, 710–722 (2010). 20448178 10.1126/science.1188021 PMC5100745

[67] S. Liu , Demographic history and natural selection shape patterns of deleterious mutation load and barriers to introgression across populus genome. Mol. Biol. Evol. **39**, msac008 (2022). 35022759 10.1093/molbev/msac008 PMC8826634

[68] M. W. Libbrecht, W. S. Noble, Machine learning applications in genetics and genomics. Nat. Rev. Genet. **16**, 321–332 (2015). 25948244 10.1038/nrg3920 PMC5204302

[69] W. Yang , Target-oriented prioritization: Targeted selection strategy by integrating organismal and molecular traits through predictive analytics in breeding. Genome Biol. **23**, 80 (2022). 35292095 10.1186/s13059-022-02650-w PMC8922918

[70] C.-Y. Cheng , Evolutionarily informed machine learning enhances the power of predictive gene-to-phenotype relationships. Nat. Commun. **12**, 5627 (2021). 34561450 10.1038/s41467-021-25893-w PMC8463701

[71] G. M. Janzen, L. Wang, M. B. Hufford, The extent of adaptive wild introgression in crops. New Phytol. **221**, 1279–1288 (2019). 30368812 10.1111/nph.15457

[72] P. Ramu , Cassava haplotype map highlights fixation of deleterious mutations during clonal propagation. Nat. Genet. **49**, 959–963 (2017). 28416819 10.1038/ng.3845

[73] C. Zhang , Genome design of hybrid potato. Cell **184**, 3873–3883.e12 (2021). 34171306 10.1016/j.cell.2021.06.006

[74] H. Xiao , genome wide sequencing of *Vitis* sp. NCBI BioProject. https://dataview.ncbi.nlm.nih.gov/object/PRJNA910315. Deposited 9 December 2022.

[75] H. Xiao , genome wide sequencing of *Vitis* sp. NGDC BioProject. https://ngdc.cncb.ac.cn/gsub. Deposited 28 April 2023.

[76] P. Danecek , The variant call format and VCFtools. Bioinformatics. **27**, 2156–2158 (2011). 21653522 10.1093/bioinformatics/btr330 PMC3137218

[77] B. Q. Minh , IQ-TREE 2: New models and efficient methods for phylogenetic inference in the genomic Era. Mol. Biol. Evol. **37**, 1530–1534 (2020). 32011700 10.1093/molbev/msaa015 PMC7182206

[78] L. Excoffier, I. Dupanloup, E. Huerta-Sánchez, V. C. Sous, M. Foll, Robust demographic inference from genomic and SNP data. PLoS Genet. **9**, e1003905 (2013). 24204310 10.1371/journal.pgen.1003905 PMC3812088

[79] L. Excofffier , fastsimcoal2: Demographic inference under complex evolutionary scenarios. Bioinformatics. **37**, 4882–4885 (2021). 34164653 10.1093/bioinformatics/btab468 PMC8665742

[80] B. C. Haller, P. W. Messer, SLiM 3: Forward genetic simulations beyond the wright-fisher model. Mol. Biol. Evol. **36**, 632–637 (2019). 30517680 10.1093/molbev/msy228 PMC6389312

[81] H. Xiao , analysis-code-for-introgression-in-grapes. GitHub. https://github.com/zhouyflab/Grapevine_Adaptive_Maladaptive_Introgression. Deposited 11 December 2022.

