# Supplementary material for: Adaptive and maladaptive introgression in grapevine domestication
Source: Proc Natl Acad Sci U S A. 2023 Jun 5;120(24):e2222041120. doi: 10.1073/pnas.2222041120 (PMC10268302; doi:10.1073/pnas.2222041120)
Supplement: Supplementary file 1 — Appendix 01 (PDF) [file pnas.2222041120.sapp.pdf]

Supporting Information for

Adaptive and maladaptive introgression in grapevine domestication

Hua Xiao, Zhongjie Liu, Nan Wang, Qiming Long, Shuo Cao, Guizhou Huang, Wenwen Liu, Yanling Peng, Summaira Riaz, Andrew M. Walker, Brandon S. Gaut and Yongfeng Zhou

Yongfeng Zhou; Brandon S. Gaut
Email: *zhouyongfeng@caas.cn; bgaut@uci.edu*

This PDF file includes:

Supporting text
Figures S1 to S36
Tables S1 to S10
Legends for Movies S1 to S3
Legends for Datasets S1 to S4
SI References

Other supporting materials for this manuscript include the following:

Movies S1 to S3
Datasets S1 to S4

Supporting Information Text

Supplementary Methods

Plant samples and sequencing

A total of 40 grape samples were sampled from the USDA grape germplasm collections in Davis, California (Table S1). Genomic DNA was isolated from leaf samples with the Qiagen DNeasy plant kit. Paired-end sequencing libraries were constructed with an insert size of 300-400 bp according to the Illumina library preparation protocols. Libraries were then sequenced using the Illumina HiSeq 4000 platform with 150-bp paired reads to a target coverage of 30x. The raw sequencing data have been deposited in the Short Read Archive at NCBI under BioProject ID: PRJNA910315 (1) and the National Genomics Data Center (NGDC) Genome Sequence Archive with BioProject number: PRJCA016655 (2). We also used Illumina raw reads for previous publications that were downloaded from the Short Read Archive at NCBI. Altogether we analyzed 108 wild accessions, 231 cultivars, and 6 outgroup species (Table S1).

To verify that the *sylvestris* samples represent the breadth of its distribution, we predicted the distribution of wild grapes in Europe and the Middle East using the program MaxEnt (3). MaxEnt counts the maximum entropy modeling of geographic distributions of species based on environmental data and the known coordinates of wild grapes. The environmental variables data were downloaded from WorldClim (4). The coordinate data of *sylvestris* were downloaded from Global Biodiversity Information Facility (GBIF, https://www.gbif.org). The prediction utilized 10 jackknife replicates and a random test percentage of 25%.

SNP Calling, filtering and annotation

For all resequencing data, Trimmomatic-0.35 was used to remove adapter sequences and bases with average qualities < 20 in 4-bp windows of the paired raw sequencing reads (5). Reads with length < 60 bases were discarded, as were unpaired reads. Trimmed reads were mapped to the *V.vinif*era reference genome (6) with the BWA-MEM2 algorithm of the bwa- 0.7.17 (7), using default parameters. MarkDuplicates of GATK-4.2.3.0 was used to identify and remove duplicate reads (8).

The VC function implemented in GTX (http://www.genetalks.com/gtxlab.html), which is based on the Haplotype Caller of GATK, was used for SNP and genotype calling for each sample. After obtaining the gvcf for each sample, the joint function in GTX was used to call SNPs jointly across all samples. Vcftools was then used to perform filtering to reduce false positives (9) in three steps. First, we removed the SNPs with more than two alleles with parameters --min-alleles 2 --max-alleles 2. Second, we removed SNPs with quality scores (GQ) < 20 (--minGQ 20). Third, SNPs with more than 20% missing genotypes across all samples were removed, with parameters --max-missing 0.8. For some analyses, plink (www.cog-genomics.org/plink/2.0/) was used to remove strongly linked regions with parameter: --indep-pairwise 50 10 0.5. The SNPs were also phased by the software Beagle (version 5.4) with default parameters and used for some analysis (10).

Phylogeny and population structure

Phylogenetic trees were constructed using SNP data by iqtree with 1000 bootstrap replicates (11). Two kinds of models were used: VT+F+R5 and GTR+I+G. The split trees of chloroplast and mitochondria were generated by SplitsTree using SNP data filtered by vcftools with parameters: –minGQ20 –max-missing 0.8 (12). The population structure based on nuclear SNPs was determined using the program Admixture with *K* ranging from 2 to 10 (13). The SNP data used for these analyses were filtered based on LD by plink. The principal component analysis (PCA) was performed using plink with parameter: --pca 20 (14).

Population genetic analyses

Vcftools was used to count heterozygous sites with the parameter: --het. The heterozygosity of each sample was calculated by dividing the number of heterozygous sites in each sample by the number of all SNPs. The forward simulation software SLiM3 was used to evaluate the heterozygosity of crops under different propagation types: outcrossing or cloning (15). Outcrossing was evaluated under the Standard Neutral Model. 10,000 diploids were used as the ancestral population sizes for both models and 20,000 generations were used as the burn-in. After burn-in, we calculated the heterozygosity every 100 generations. An analysis of introgressed alleles was also conducted with SLiM3. In these introgression simulations model, two independent populations were initialized with the same population size (*Ne* = 1000) and length of chromosomes (10 Mb). The beneficial mutations had fixed selection coefficients of s = 0.01 and a dominance coefficient of 0.5; the deleterious mutations had a selection coefficient following a gamma distribution with an average value of -0.03 and dominance coefficient of 0 (i.e., fully recessive). We simulated with ten-fold more expected deleterious, as opposed to beneficial, mutations. To simulate introgression, the two populations first experienced a burn-in of 10**Ne* generations with outcrossing, and then a directional migration event of 17 individuals (estimated from our demography analyses) occurred from the donor to receptor population. The clonal receptor population remained outcrossing for 10 generations before becoming clonal. The codes for the simulation were on GitHub (https://github.com/zhouyflab/Grapevine_Adaptive_Maladaptive_Introgression (16)).

Sequence similarity (D_{xy}), and fixation indices (F_{ST}) were calculated using the Python script: popgenWindows.py (https://github.com/simonhmartin/genomics_general) with 50 kb nonoverlapping windows. Nucleotide diversity (π) was calculated using vcftools with parameter: --window-pi 50000. These statistics were evaluated within or between all five groups (EU, n = 72; ME1, n = 29; ME2, n = 7; wine, n = 77; and table, n = 148). We also used SMC++ to infer the demography of each of the five groups using unphased SNPs data according to the manuscript with default parameters (17). The mutation rate was set to 7 x 10^{-9} and a generation time of 2.5 years was used to convert the generations into years (18). Identity By Descent (IBD) was calculated using plink with parameter: --genome. The average values within or between groups were calculated for presentation.

The continuous-time sequential Markovian coalescent approximation implemented fastsimcoal (version: fsc27) was used to further estimate the population history under complex evolutionary scenarios (19, 20). Before performing the analysis, the derived site frequency spectrum (unfolded SFS) was generated using a

Python script: superSFS (https://github.com/zhouyflab/Grapevine_Adaptive_Maladaptive_Introgression (16)). This script can infer ancestral alleles according to outgroup species and count the frequency of the derived allele in each group. We then used fastsimcoal to estimate the unfolded SFS under different evolutionary models. Monomorphic sites and SFS entries with less than 20 SNPs were ignored. Each model was based on 100,000 coalescent simulations with 50 optimizations to estimate parameters for each scenario. The histories of a single population, population differentiation without gene flow, and population differentiation with gene flow have been estimated. The command line for running fastsimcoal is: fastsimcoal -t model.tpl -e model.est -d -0 -C 10 -n 100000 -L 50 -s 0 -M -c20 -B40.

The genomic databases with SIFT predictions were generated by a tool, make-SIFT-db-all.pl, using genomic sequence data in Fasta format and the gene annotation file in gtf format (21). The protein database required by the tool was downloaded from UniProt (https://www.uniprot.org/) (22). Then the SNP data in vcf format and the genomic databases were input into SIFT 4G for deleterious mutation assessment. For each individual, the homozygous sites predicted as deleterious were counted as recessive deleterious mutation, and the additive deleterious mutation was counted by the number of heterozygous deleterious mutation sites plus two times the number of homozygous deleterious mutation sites (6). The LD decay was evaluated and plotted using PopLDdecay (https://github.com/BGI-shenzhen/PopLDdecay) (23). We evaluated LD every 1Mb sliding window with 1Mb step sites.

Selective sweep detection and analysis

Population branch statistic (PBS) analysis was performed by the program PBScan (https://github.com/thamala/PBScan) (24). Six individuals in other species (3 *Vitis californica* samples and 3 *Muscadinia rotundifolia* samples) in the genus *Vitis* were used as the outgroup. The PBS was calculated using the wine or table group as POP1, ME2 as POP2, and EU as POP3. Each window contained 50 SNP with 50 SNP step sizes.

We also used the machine learning-based selective sweep analysis program diploSHIC/SHIC (25, 26). This program predicted the selection types using the classifier that was trained on simulated data. Before the prediction, we simulated training and testing data in different categories – i.e., neutral, soft-linked, hard-linked, soft and hard - using the program discoal (https://github.com/kr-colab/discoal). The demographic parameters for simulation were based on the results of SMC++. The scripts for the simulation are available on GitHub (https://github.com/zhouyflab/Grapevine_Adaptive_Maladaptive_Introgression (16)). Using this simulated data, we then used diploSHIC to make predictions that were performed as follows: First, the feature vectors of simulated data were generated using fvecSim mode in diploSHIC. Second, training and testing sets were generated using makeTrainingSets mode in diploSHIC. Third, both training data and testing data were input into diploSHIC using the training mode to train the classifier. Fourth, the feature vectors of each grape population were calculated using fvecVcf mode by inputting the SNP information stored within the VCF format. Finally, the classifiers trained in step three were applied to predict the sweep selection types of different regions in each group by inputting the feature vector data generated in step four using the prediction

mode of diploSHIC. The default parameters were used for running diploSHIC. When calculating the feature vectors, 5kb sliding windows with 5 kb step size were used.

Introgression analyses

We used the program Dsuite (https://github.com/millanek/Dsuite) to calculate Patterson's D (ABBA-BABA) and f4-ratio statistics across populations to evaluate introgression probabilities across the genome (27). When performing the analysis, the SNP data and a tree in the newick format showing the relationship between groups were used as input files. The tree was written based on the phylogenetic tree generated by iqtree, as mentioned previously. The fb-statistic was calculated based on f4-ratio values. For inferring introgression by windows, we used the program popgenWindows.py (https://github.com/simonhmartin/genomics_general), which calculates fd and fdM values. These values were calculated among ME2, wine, and EU groups, among ME2, table, and EU groups, or among table, wine, and EU groups. The fd and fdM statistic values were calculated for every 50kb window.

We also used the machine learning-based introgression analysis program Filet to predict introgressed regions (28). Prior to prediction, we first generated training data via simulation. Msmove was used to simulate the history between EU and wine groups under scenarios of no gene flow, gene flow from EU to wine, and gene flow from wine to EU, using the parameters estimated by fastsimcoal previously (29, 30). The scripts for running Msmove were uploaded to GitHub (https://github.com/zhouyflab/Grapevine_Adaptive_Maladaptive_Introgression (16)). Then, the classifier was trained and used for prediction as follows: First, the summary statistics of simulated data were calculated by Msmove using the normalizeTwoPopnStats.py script in Filet. Next, the summary statistics were aggregated into one labeled data matrix to be used for training using the buildThreeClassTrainingSet.py script in Filet. Third, the classifier was trained using the trainFiletClassifier.py script. Fourth, phased sequence data for EU and wine groups and inferred ancestral sequences were generated in Fasta format. Masking was also performed using the TE annotation information. Then the Fasta files were used for calculating the summary statistics in non-overlapping 10kb sliding windows by pgStatsBedSubpop_forML script and normalized by normalizePgStats.py script. Finally, the data from the previous step were input into the trained classifier for classifications, using the classifyChromosome.py script. The default parameters were used for running the Filet pipeline. As for generating the inferred ancestral sequences, we modified the reference genome at the sites that the alleles in 6 outgroup individuals more than 2. As for the classifications, there are three kinds of possibilities for each 10kb region: no introgression, introgression from EU to wine, and introgression from wine to EU. We considered a window to be introgressed only when the inferred probability of introgression was > 0.95.

To calculate introgressed allele frequencies and heterozygosity in introgressed regions, we analyzed sites that have a specific genotype in EU grapes relative to ME grapes. Alleles not existed in the ME2 groups and more than 50% in EU groups were defined as specific genotypes. The frequency of these specific genotypes in wine grapes and the ratio of individuals containing the specific genotypes were calculated for presentation.

The heterozygosity of introgressed alleles was calculated by dividing the number of heterozygous introgressed sites by the total number of introgressed sites in each individual.

Recombination analysis

We estimate the recombination rates in introgression and selection regions according to the methods in the paper of Abraham et. al (2021) (31). Firstly, the mapping data which defined the recombination rate between two markers were obtained from a previous study (32). Secondly, we transformed the location of 1662 markers in that paper onto our reference genome by sequence blast. Finally, the recombination rates of specific regions were counted in cM/kb.

GO-term enrichment

All protein sequences in the reference genome were blasted to UniProtKB/Swiss-Prot (major release 51.0) by diamond (v0.9.14.115) (33). Then based on the Gene Ontology (GO, http://geneontology.org/) databases, the results were converted into GO-Terms as the background (34, 35). The genes in putatively both introgressed and selected regions predicted by Filet and diploSHIC/SHIC, and in the 5% highest fd regions were used for GO enrichment analysis. The analysis was performed by BiNGO (3.0.3) in Cytoscape(4.1.0) (36), and Benjamini & Hochberg's FDR P-value < 0.05 of hypergeometric test threshold was used.

KINSHIP analysis

Using genotype data, the kinship matrix by the VanRaden method for relationships among individuals in the specific region was generated in GAPIT (https://github.com/jiabowang/GAPIT3). Then these data were imported into the Cytoscape for visualization, the nodes represent samples and the edge represents the kinship value.

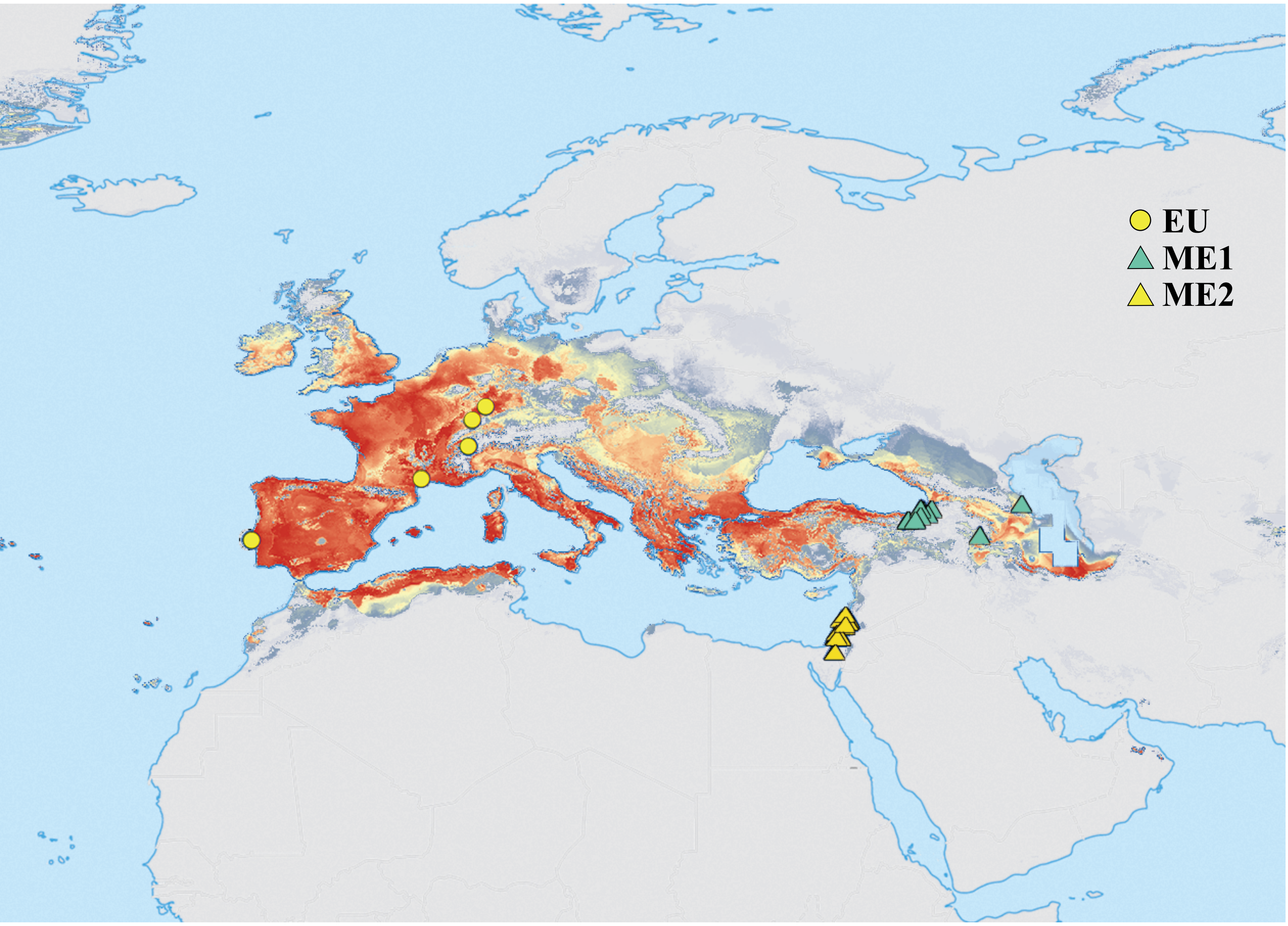

Figure S1. The distribution possibility of wild grapes in Europe and the Near East and wild samples used. The redder the color, the higher the possibility. The yellow circles indicate the EU wild grapes used in this study. Green triangles indicate ME1 wild grapes used in this study. The yellow triangles indicate the ME2 wild grapes used in this study. Most of the EU wild grapes are only labeled the approximate location due to lack of coordination.

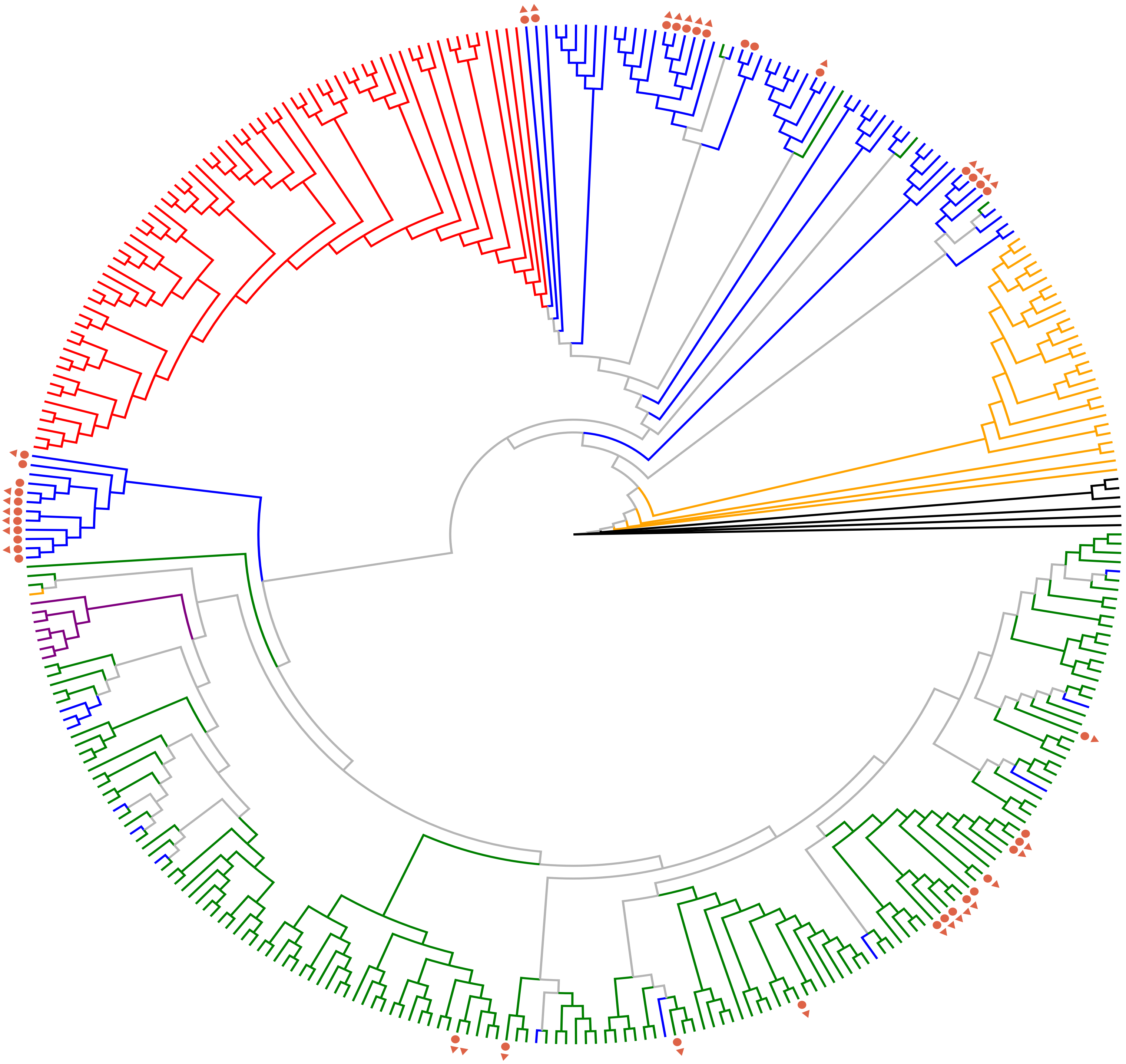

Figure S2. Phylogenetic tree generated using GTR+I+G model. The lines in different colors indicate different groups: the ME1 group is yellow, the ME2 group is purple; the EU group is reddish brown; the wine group is blue; the table group is green, and the outgroup is black. The red circles on the outside mark the domesticated grapes that their chloroplast originated from the EU grapes, and the red triangles mark the domesticated grapes that their mitochondria originated from the EU grapes.

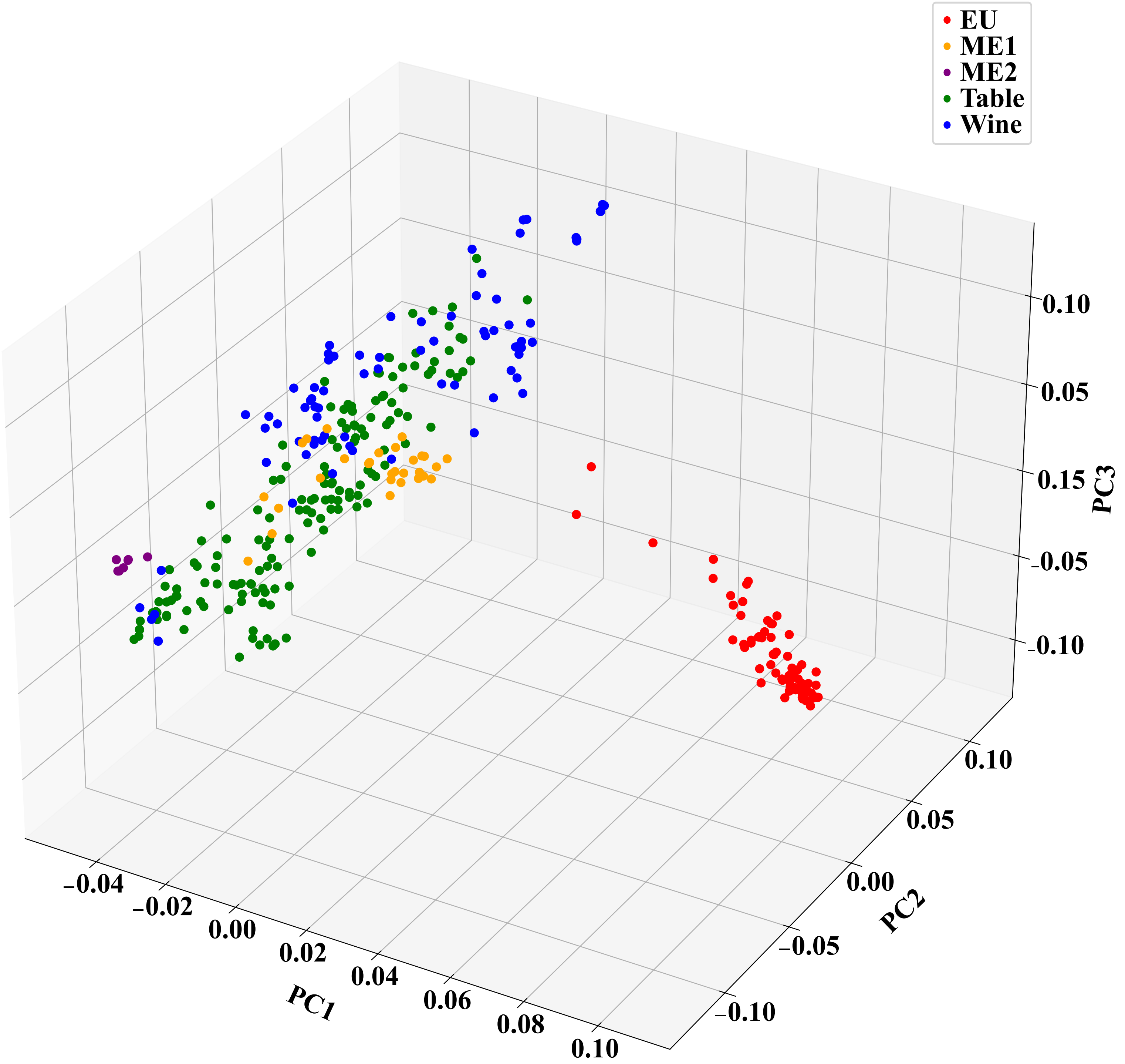

Figure S3. PCA of grape populations in three dimensions.

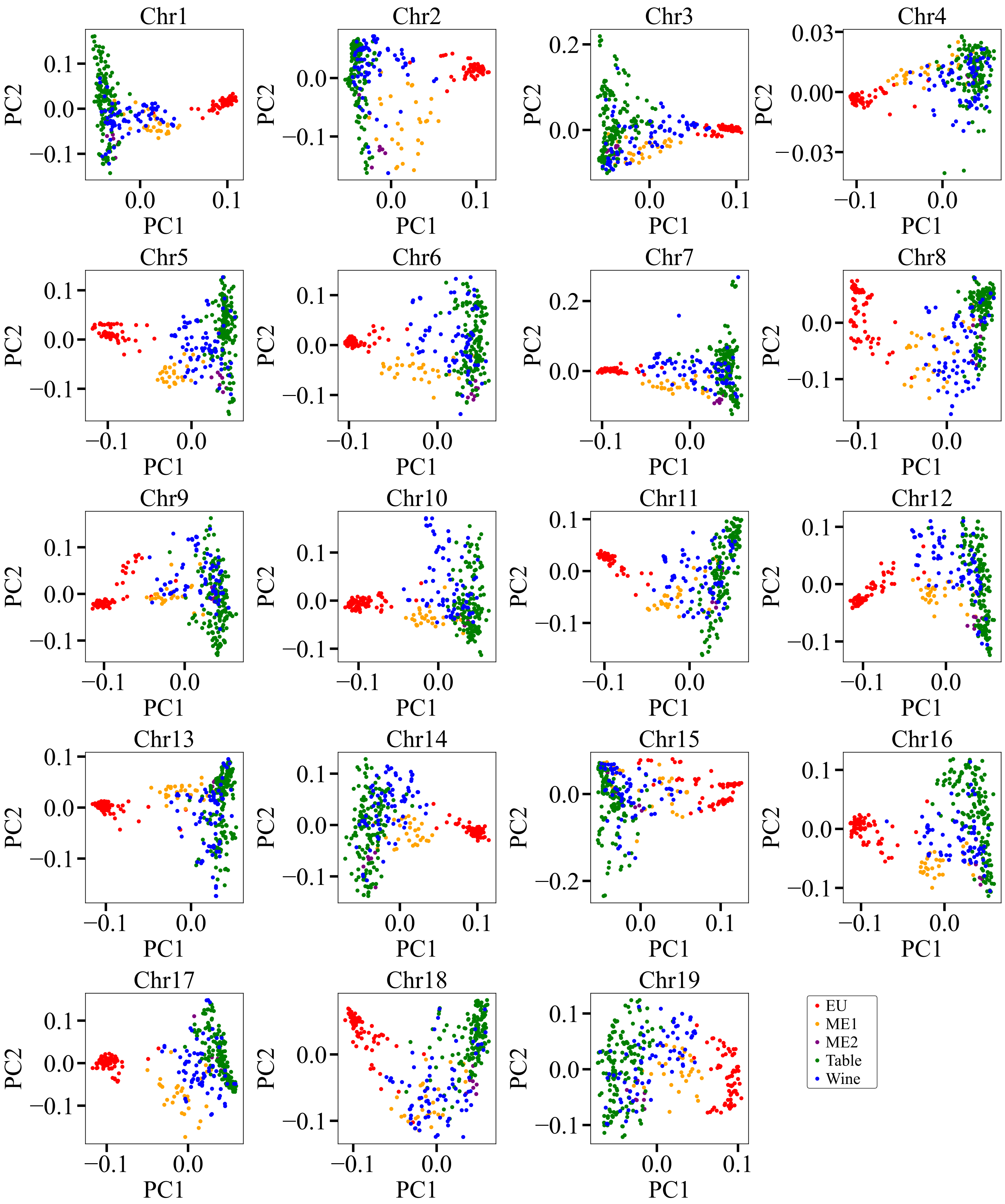

Figure S4. The principal component analysis on each chromosome.

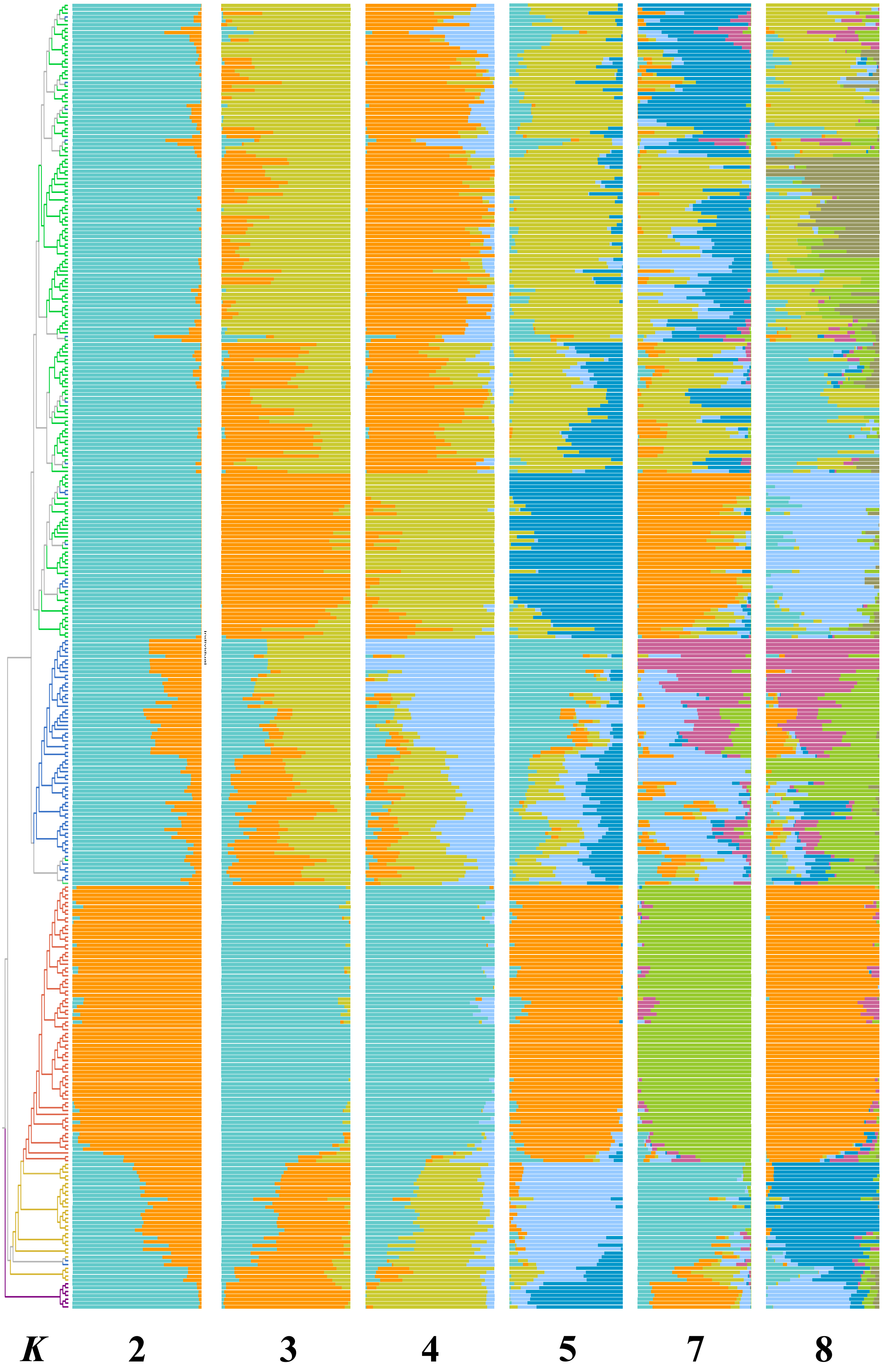

Figure S5. Structure of different *K* values. The phylogenetic tree is shown on left. The lines on the left with different colors indicate different groups: yellow, ME1 group; purple, ME2 group; reddish brown, EU group; blue, wine group; green, table group.

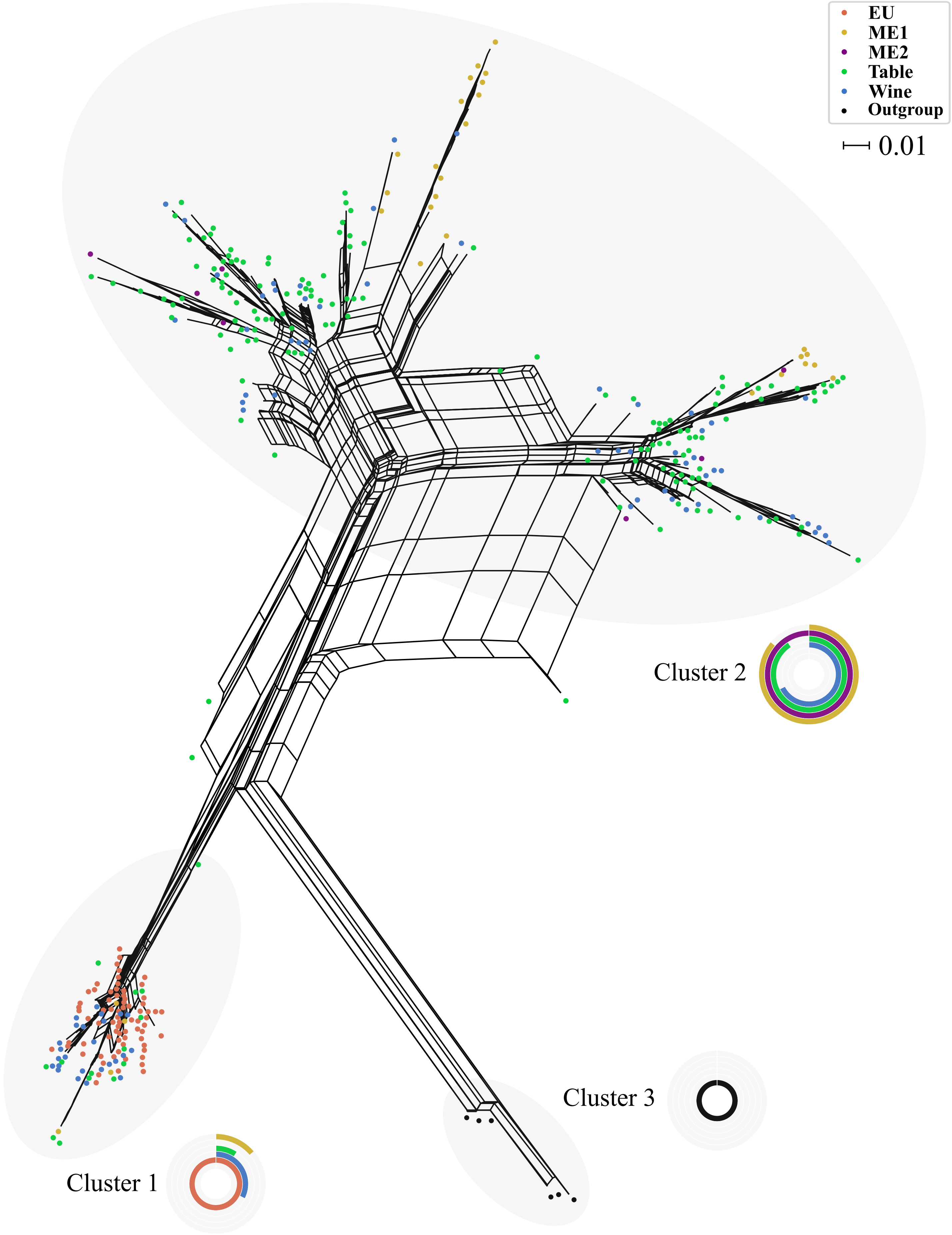

Figure S6. Splittree using chloroplast SNPs data. Different colors represent different groups: black, outgroup; yellow, ME1 group; purple, ME2 group; reddish brown, EU group; blue, wine group; green, table group. The circles near to each cluster indicate the propotion of each grape group in each cluster, from outsidie to inside: ME1, ME2, table, wine, EU, and outgroup.

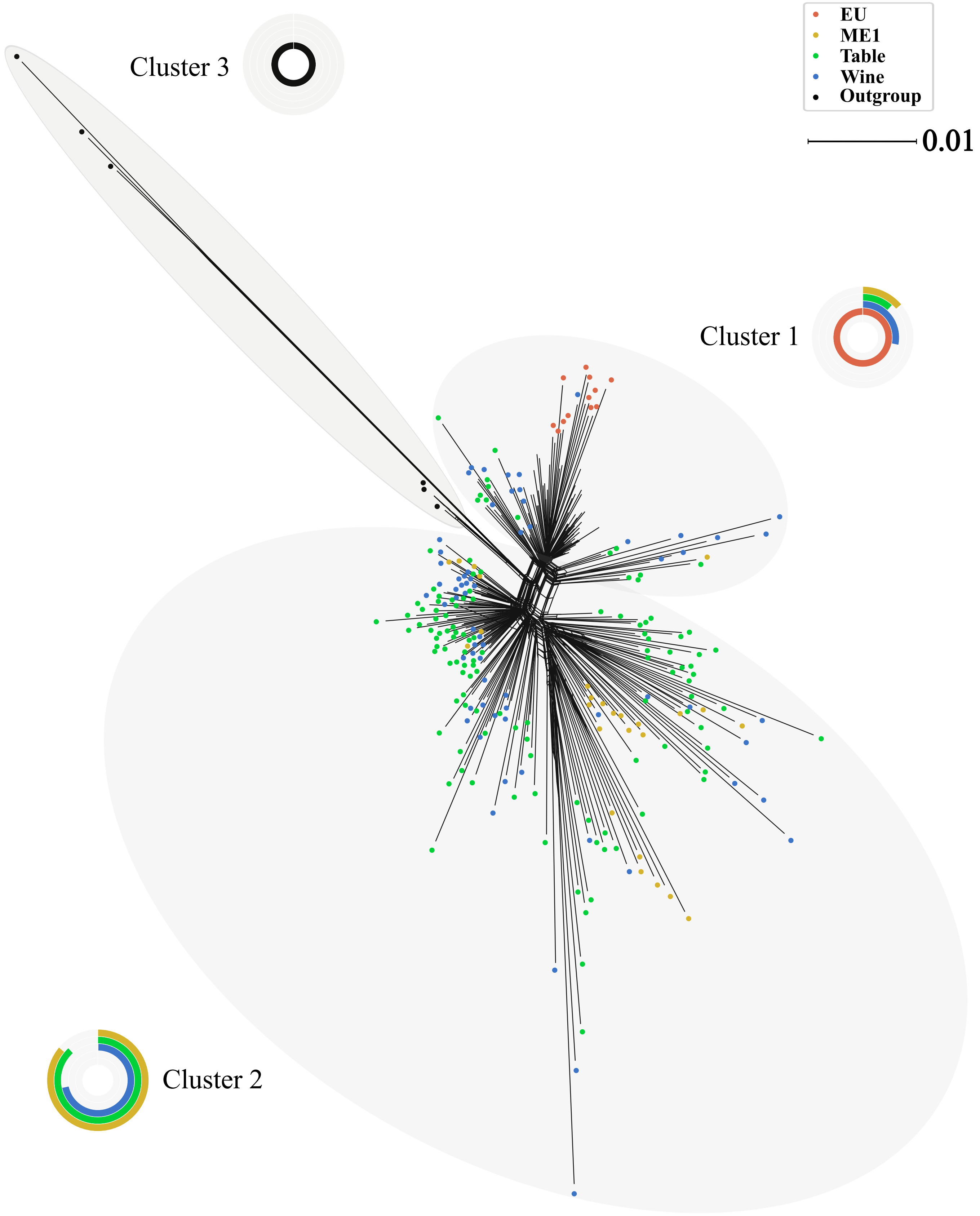

Figure S7. Splittree using mitochondria SNPs data. Different colors represent different groups: black, outgroup; yellow, ME1 group; reddish brown, EU group; blue, wine group; green, table group. The circles near to each cluster indicate the propotion of each grape group in each cluster, from outside to inside: ME1, table, wine, EU, and outgroup. No ME2 data are available here. These analysis were performed on five groups: ME1: n=29, EU: n=67 , table: n=119, wine: n=67, outgroup: n=6.

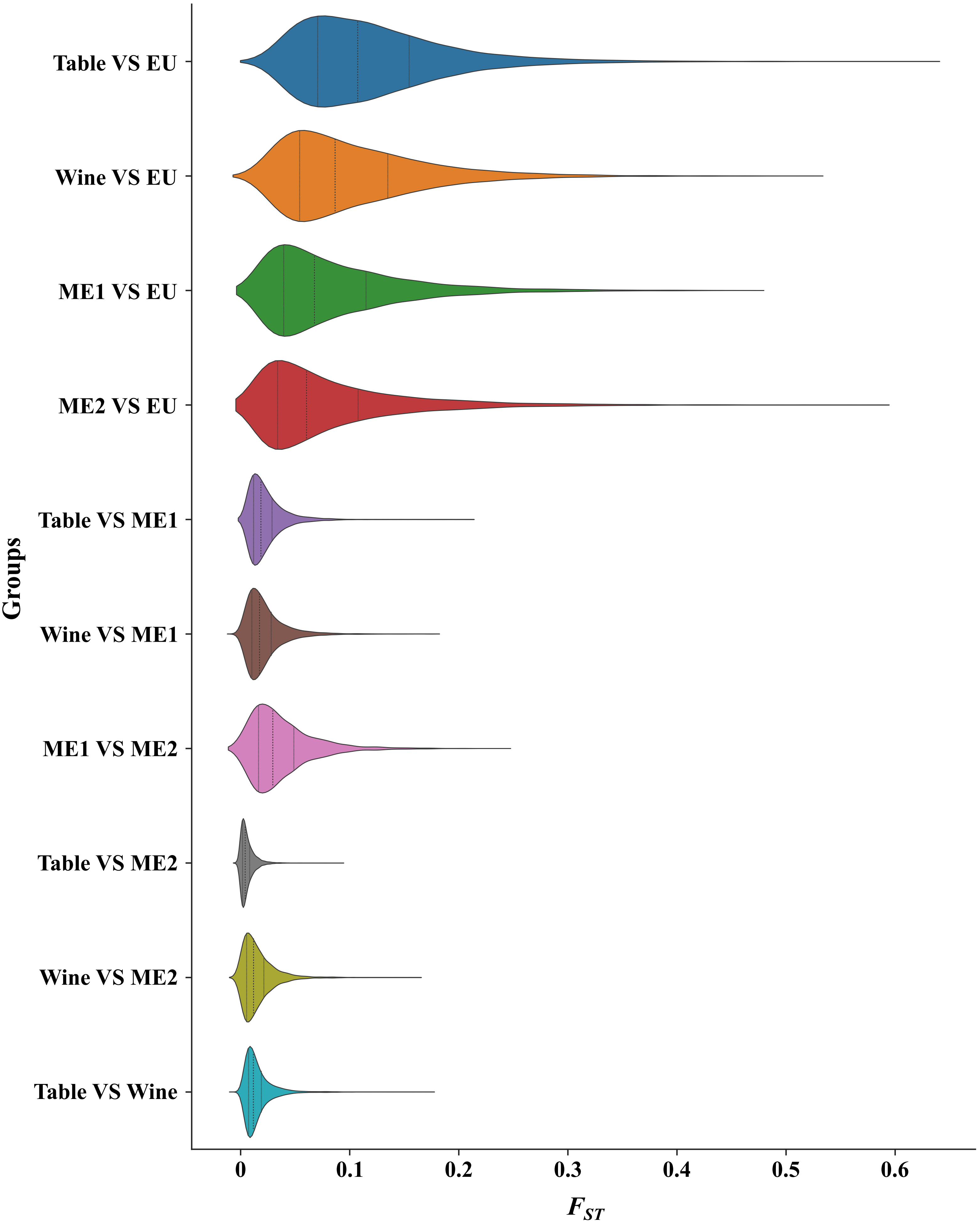

Figure S8. F_{ST} between each group. Violin plots indicate the density distribution of F_{ST} in 50 kb window between each group. The dashed lines in each violin plot represent the value of the third quartile, median, and first quartile from right to left, separately.

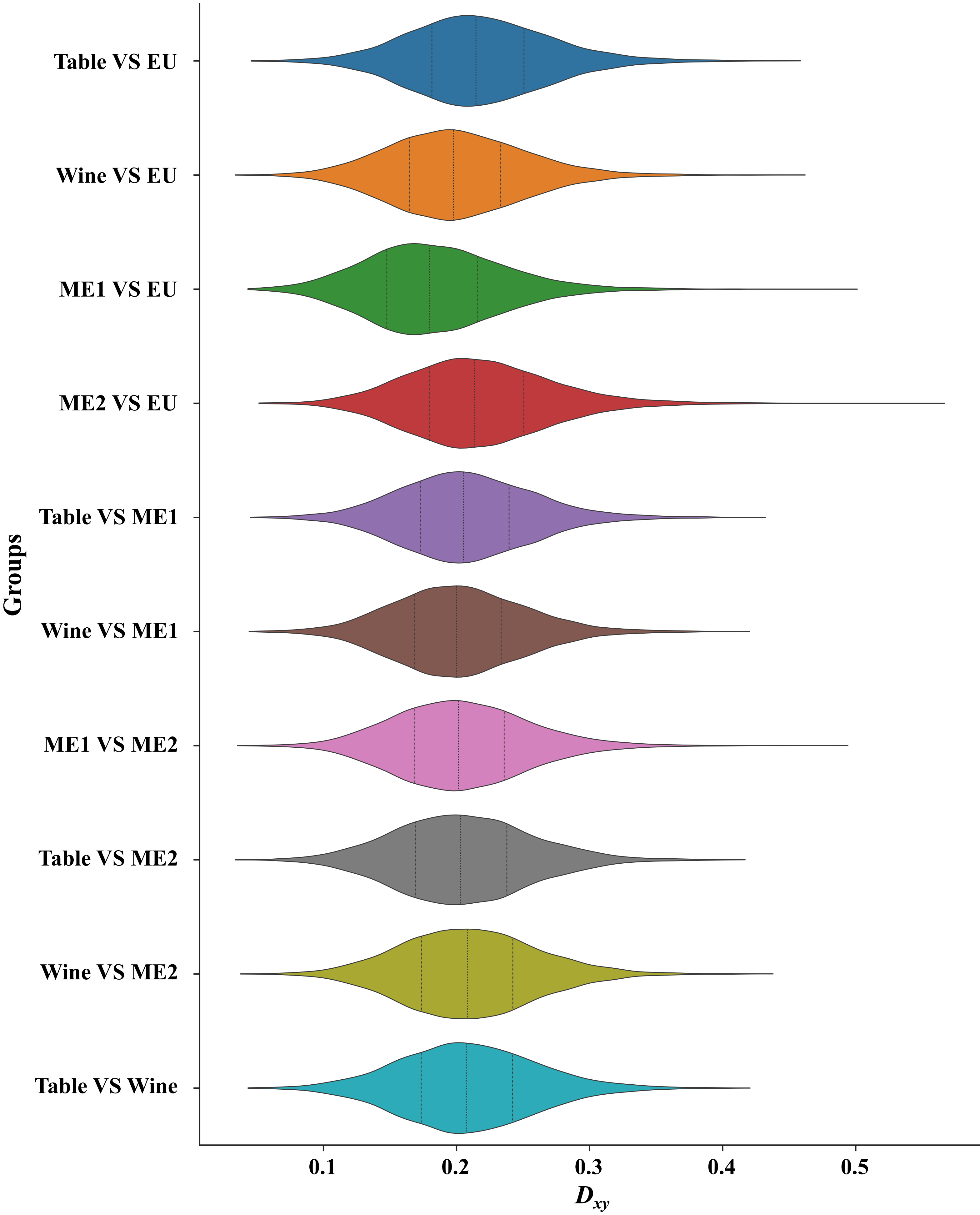

Figure S9. D_{xy} between each group. Violin plots indicate the density distribution of D_{xy} in 50 kb window between each group. The dashed lines in each violin plot represent the value of the third quartile, median, and first quartile from right to left, separately.

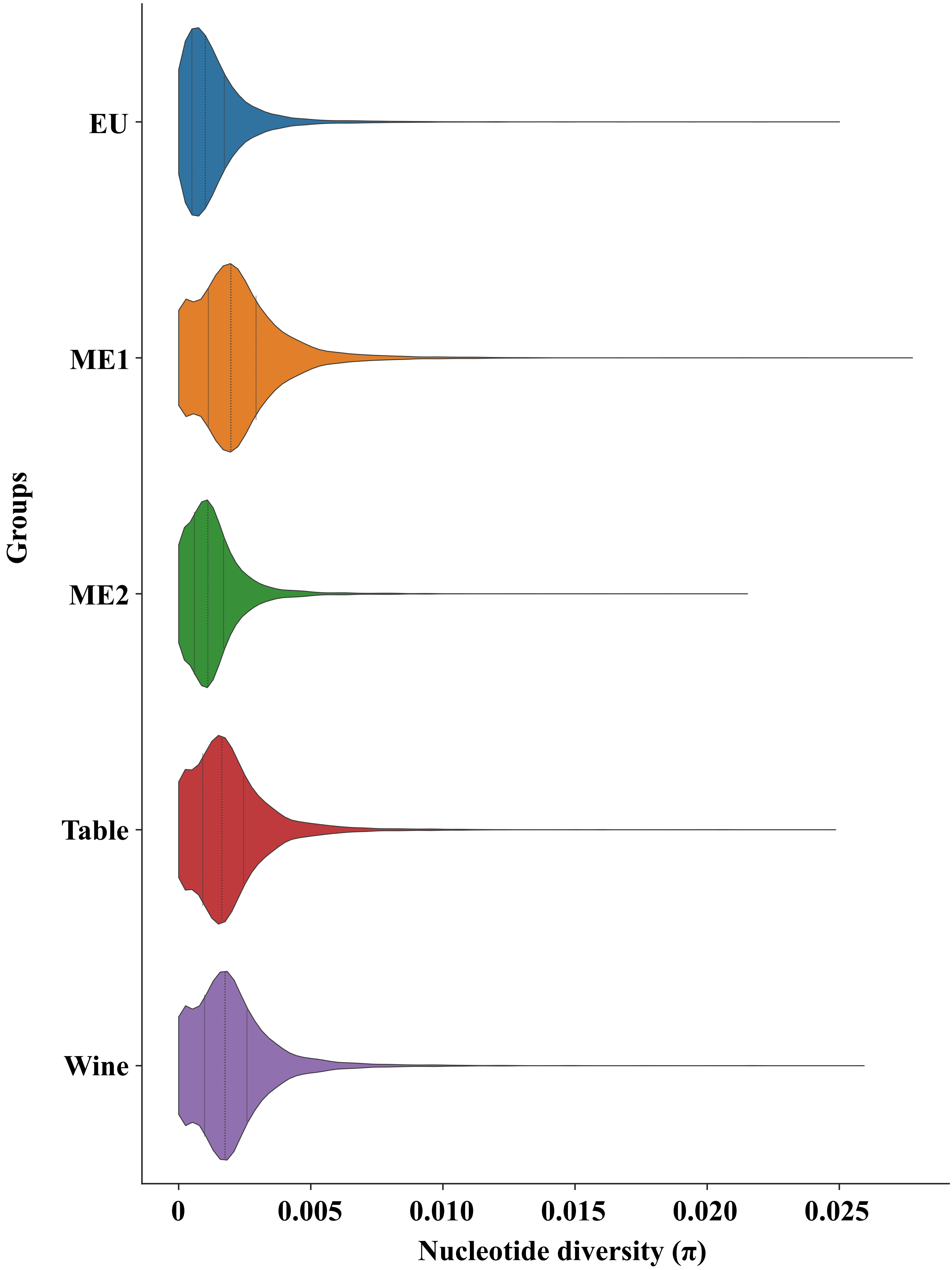

Figure S10. Nucleotide diversity (π) of different groups. Violin plots indicate the density distribution of π in 50 kb window in each group. The dashed lines in each violin plot represent the value of the third quartile, median, and first quartile from right to left, separately.

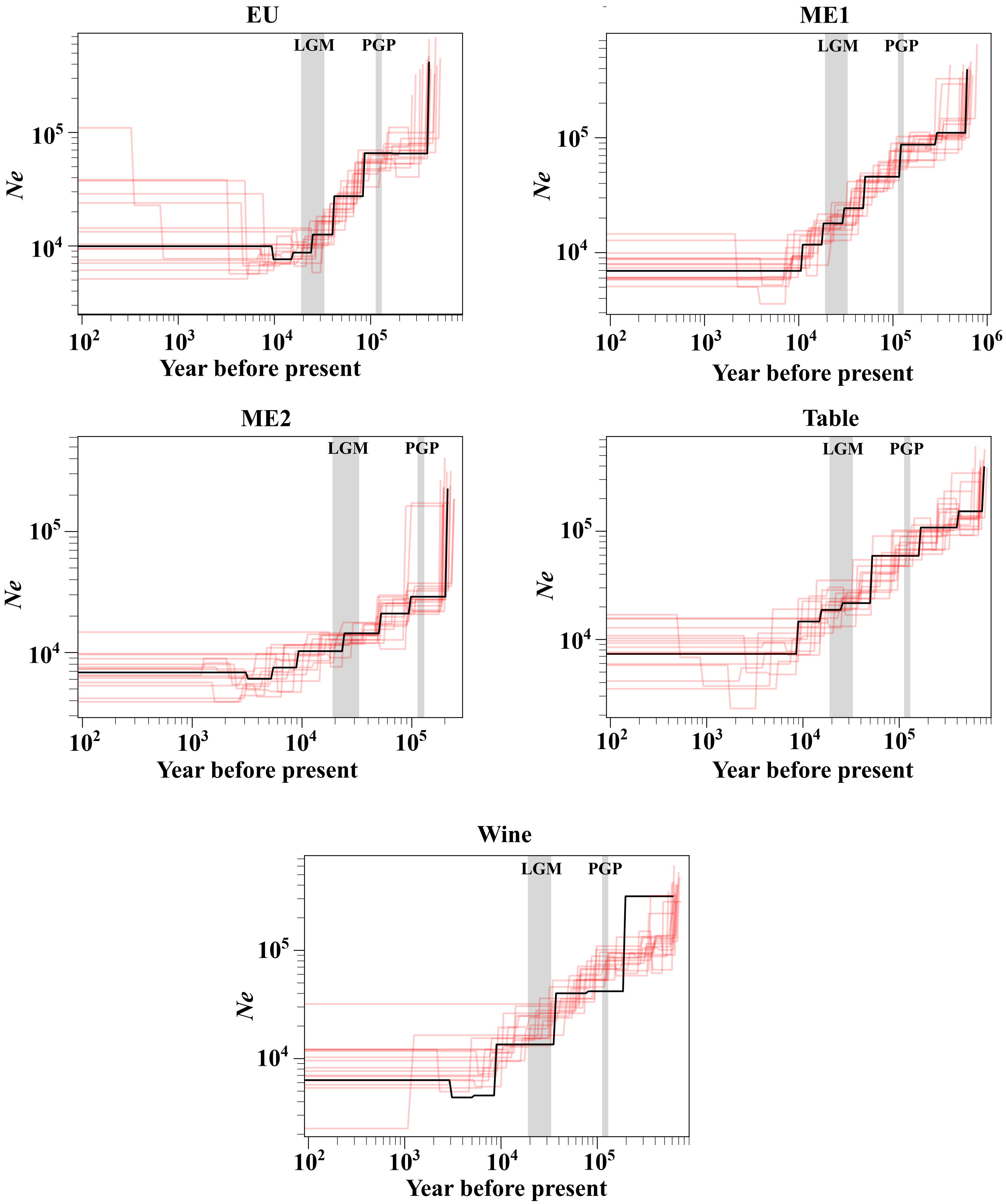

Figure S11. Effective population size (*Ne*) of each group. Red lines indicate the estimated *Ne* of different chromosomes. LGM, last glacial maximum. PGP, the penultimate glacial period.

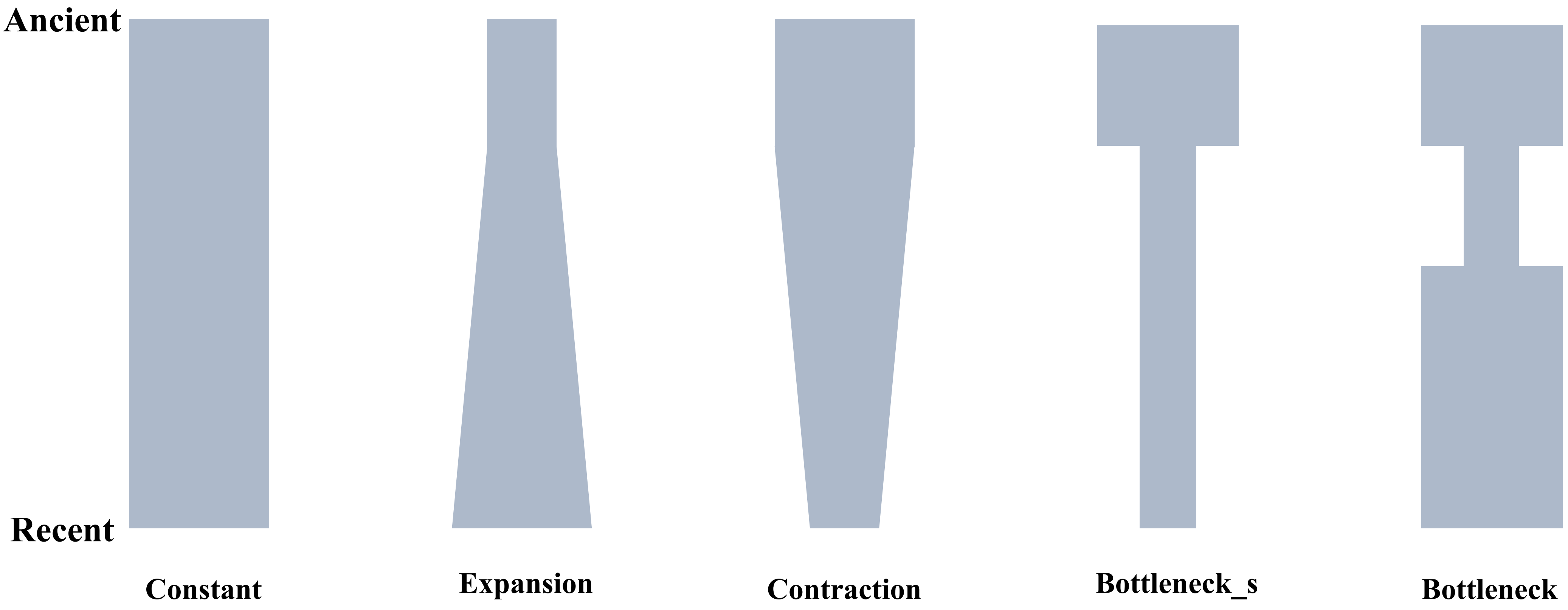

Figure S12. Five kinds of demography models.

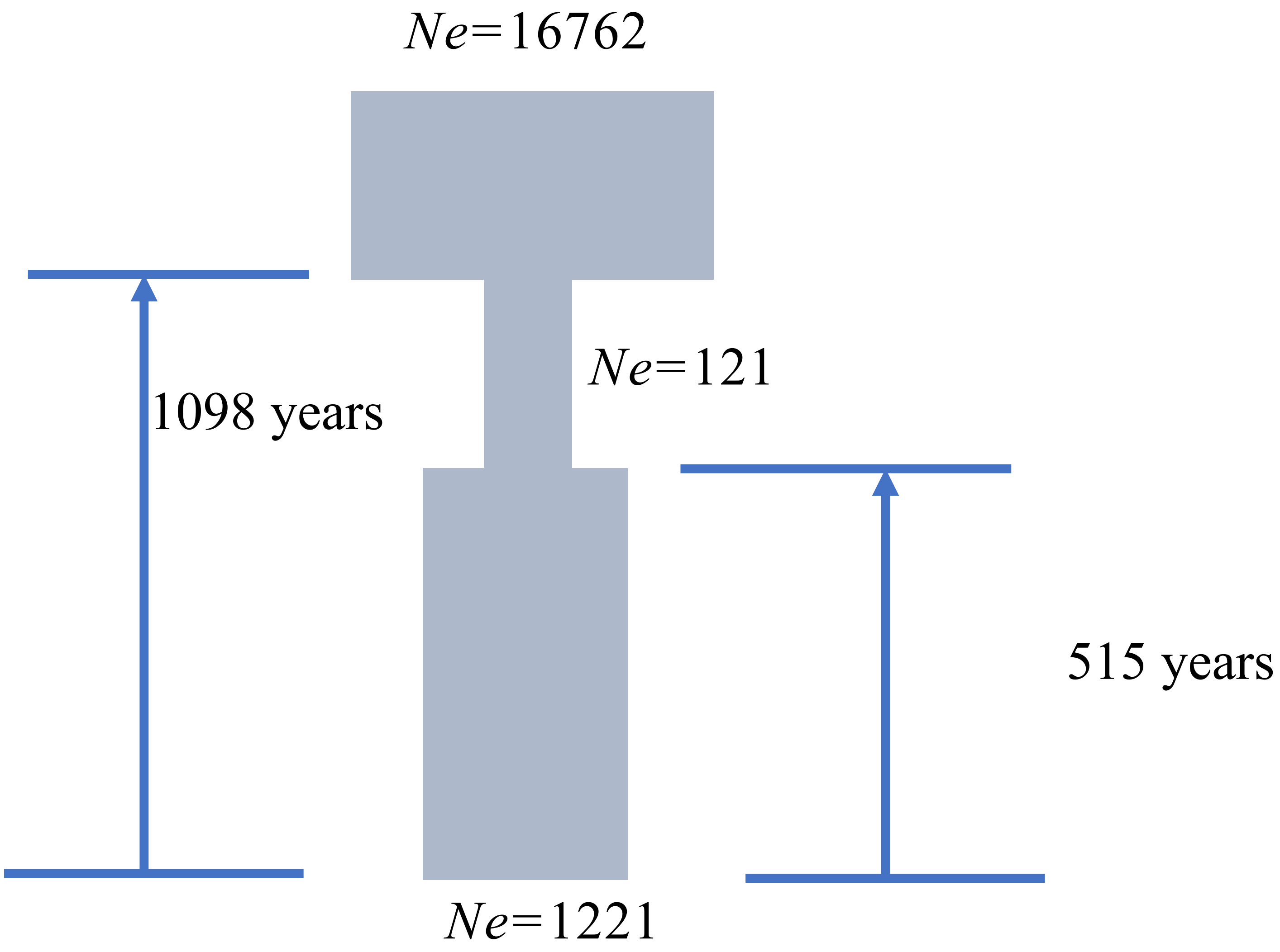

Figure S13. Demography of EU grapes**.**

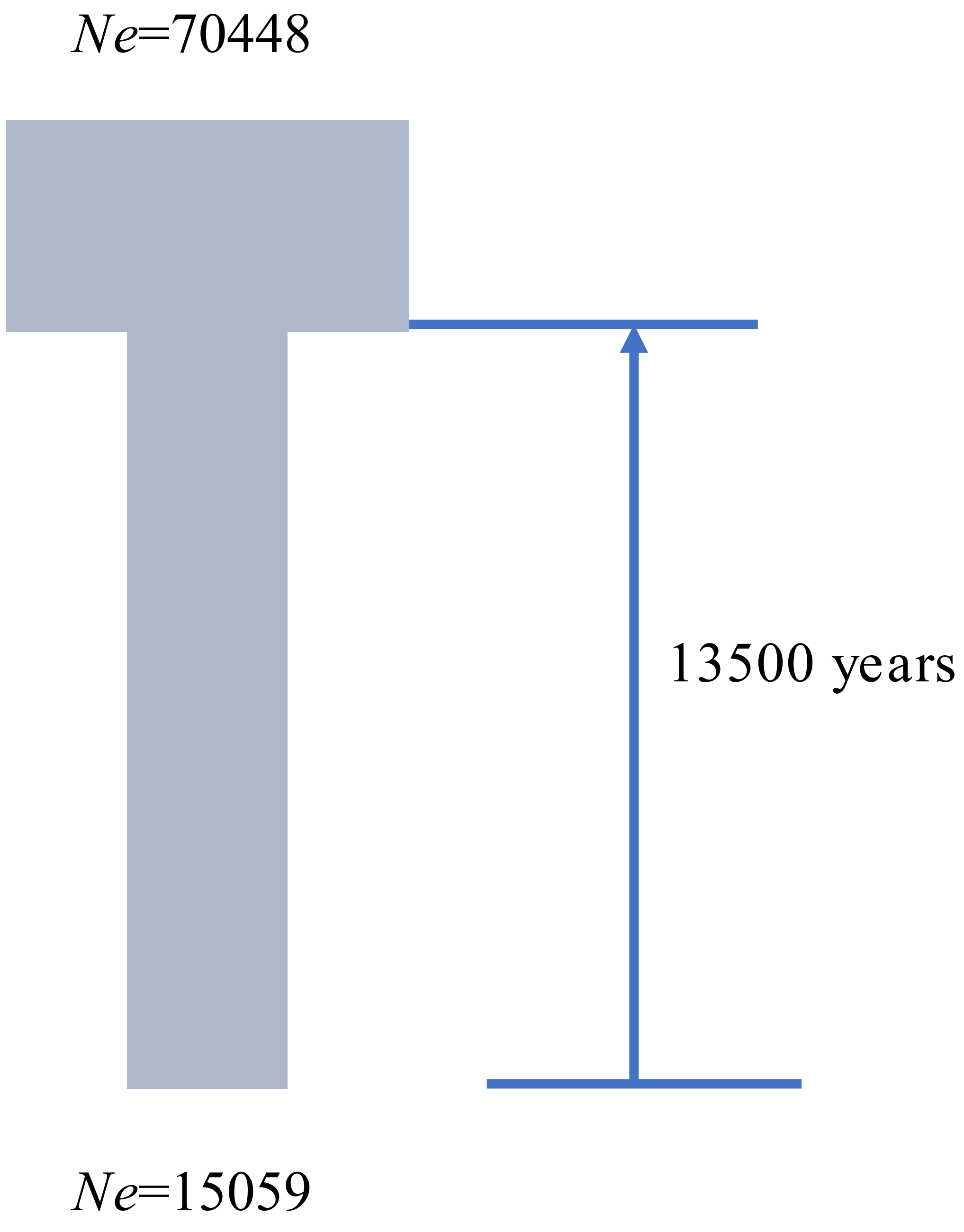

Figure S14. Demography of ME1 grapes.

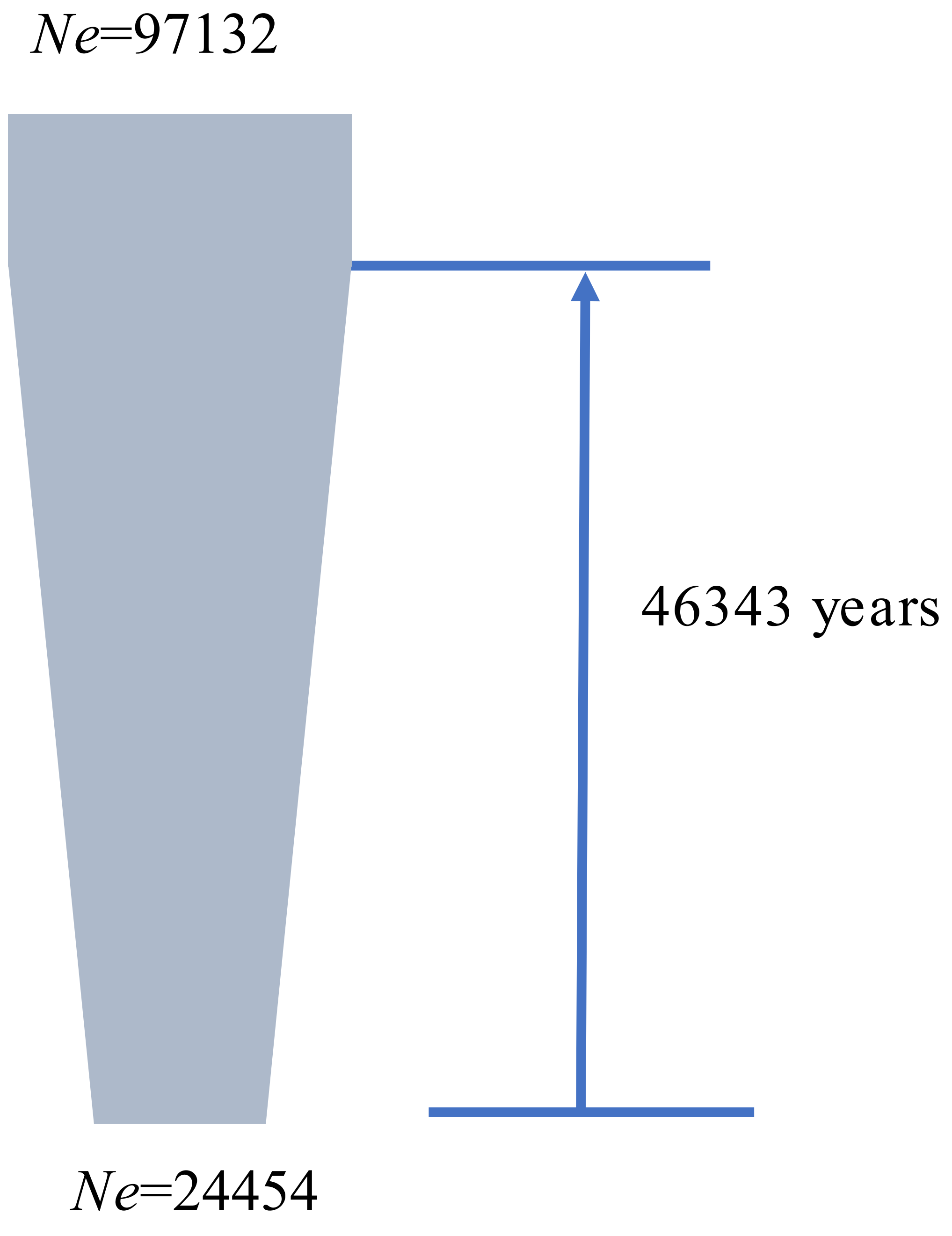

Figure S15. Demography of ME2 grapes.

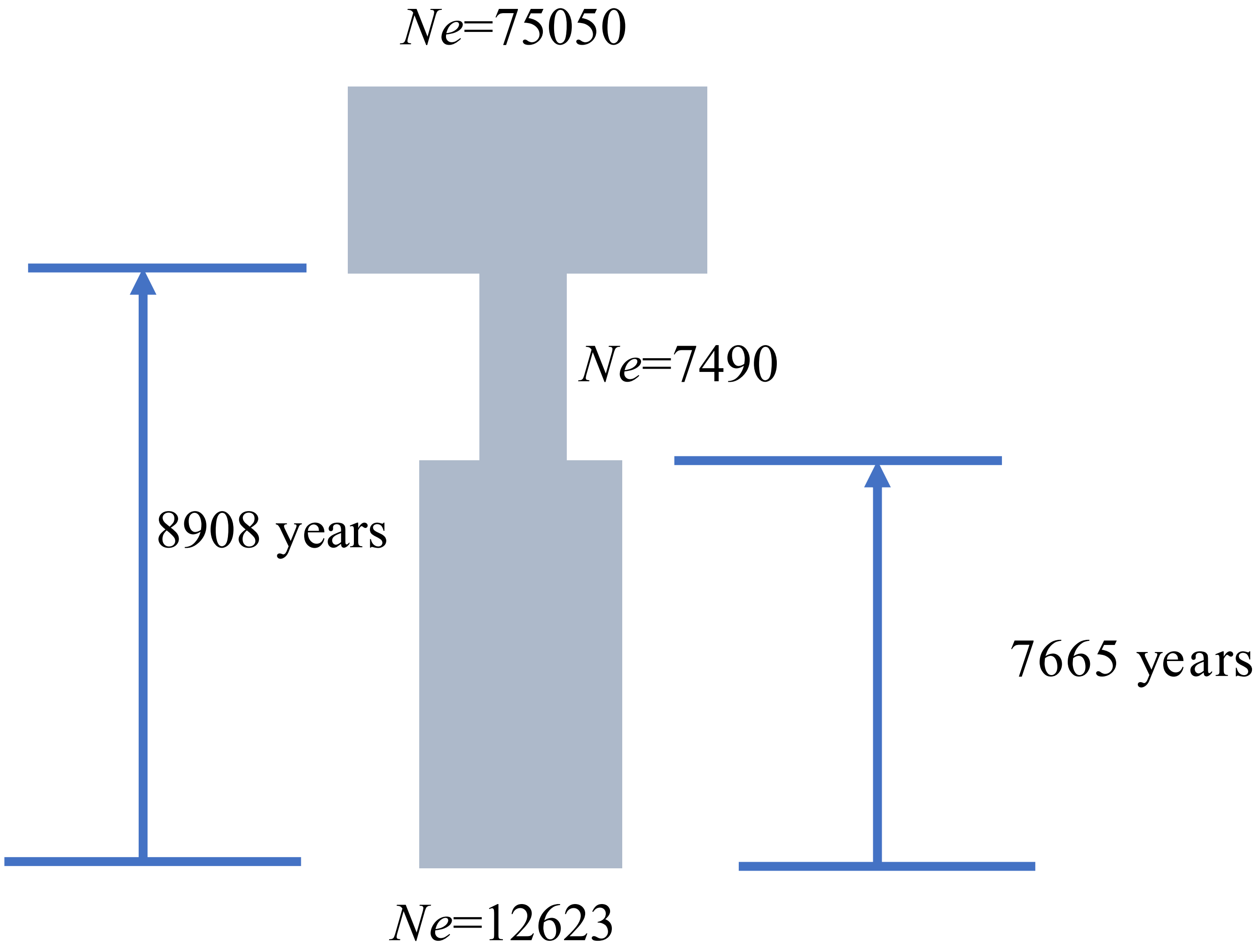

Figure S16. Demography of table grapes.

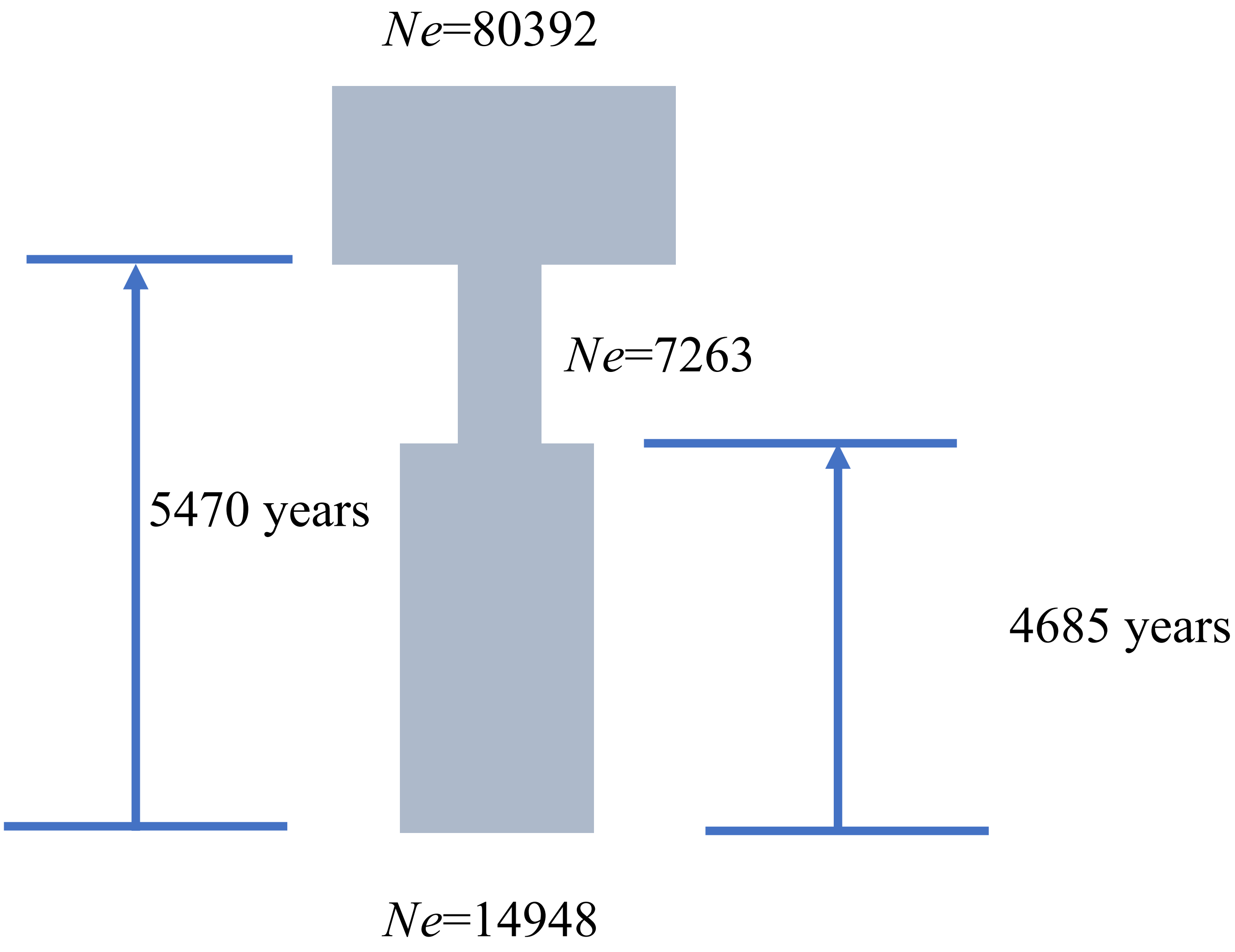

Figure S17. Demography of wine grapes.

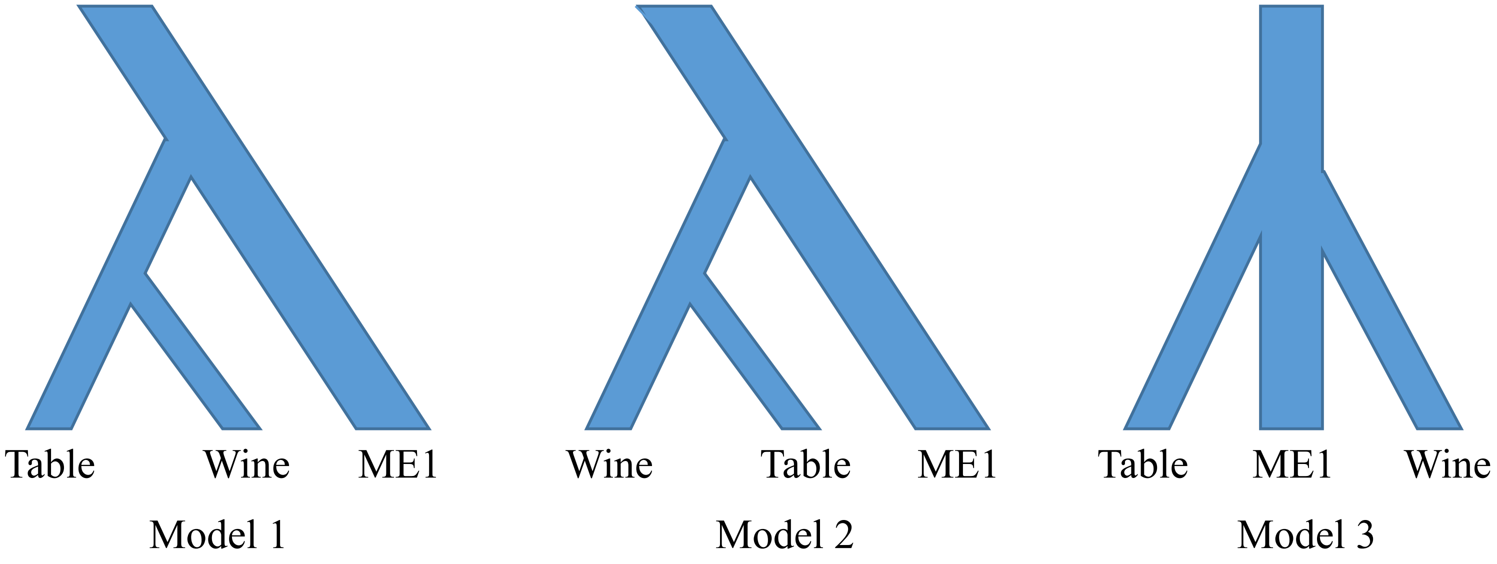

Figure S18. 6 kinds of models for estimation of the process of domestication when gene flow is not considered. Model 1 to 3: ME1 is assumed as the ancestor of domesticated grapes. Model 4 to 6: ME2 is assumed as the ancestor of domesticated grapes.

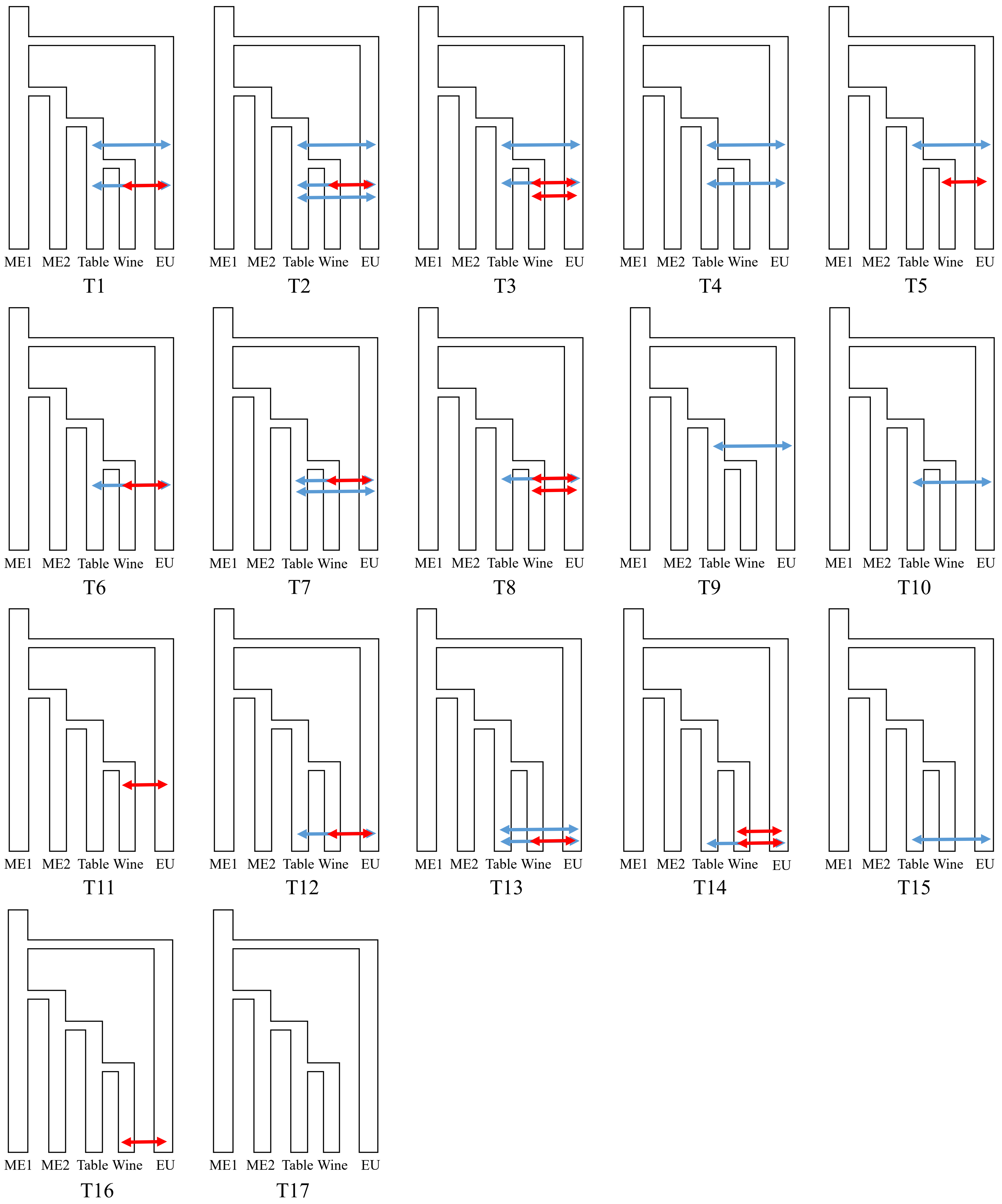

Figure S19. The models for estimation of the process of gene flow among table, wine and EU populations. ME1 population is assumed as the ancestral population. The red lines indicate introgression beween wine and EU populations, and the blue lines indicate the introgression between table and EU populations.

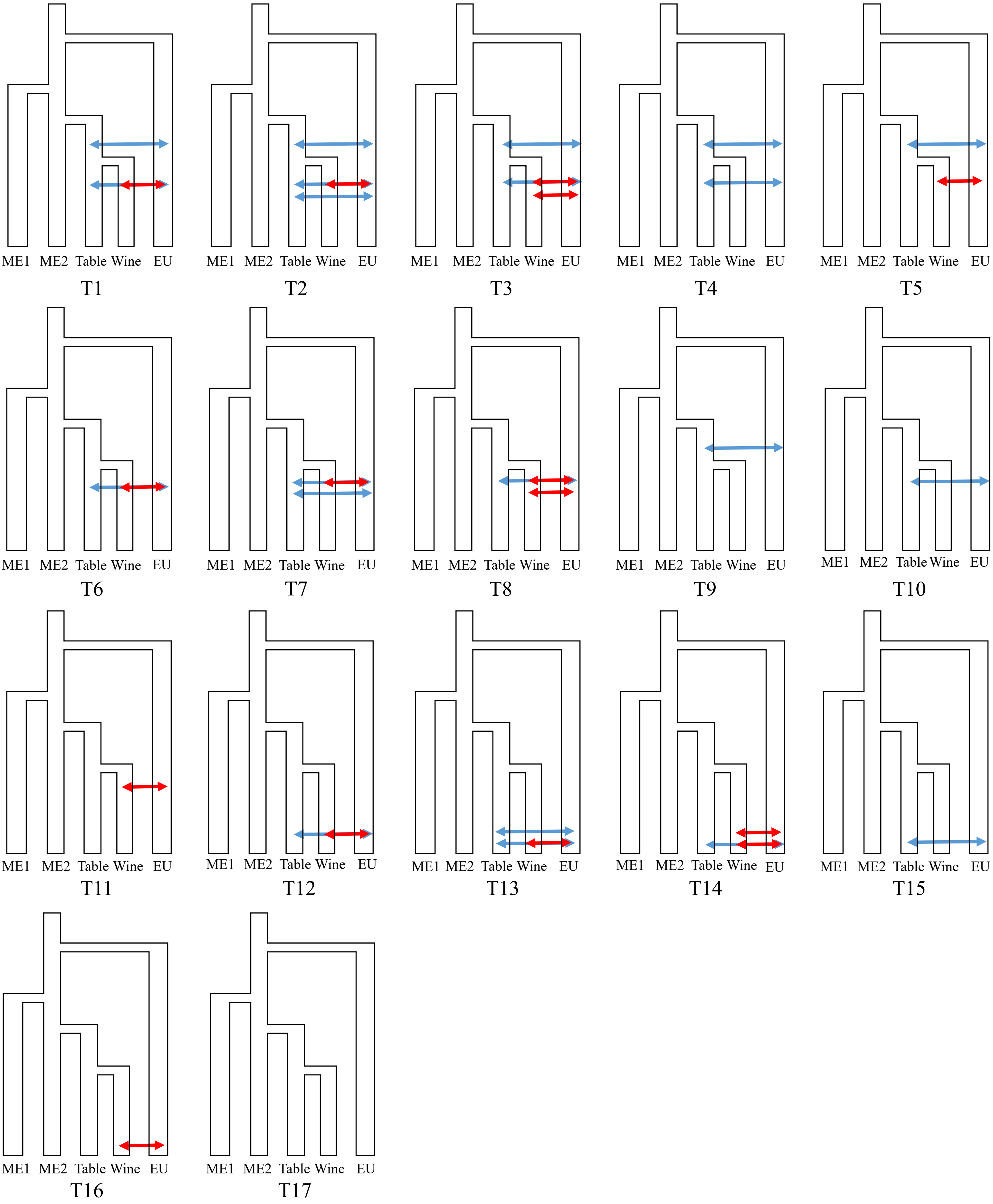

Figure S20. The models for estimation of the process of gene flow among table, wine and EU populations when ME2 is assumed as the ancestral population. The red lines indicate introgression beween wine and EU populations, and the blue lines indicate the introgression between table and EU populations.

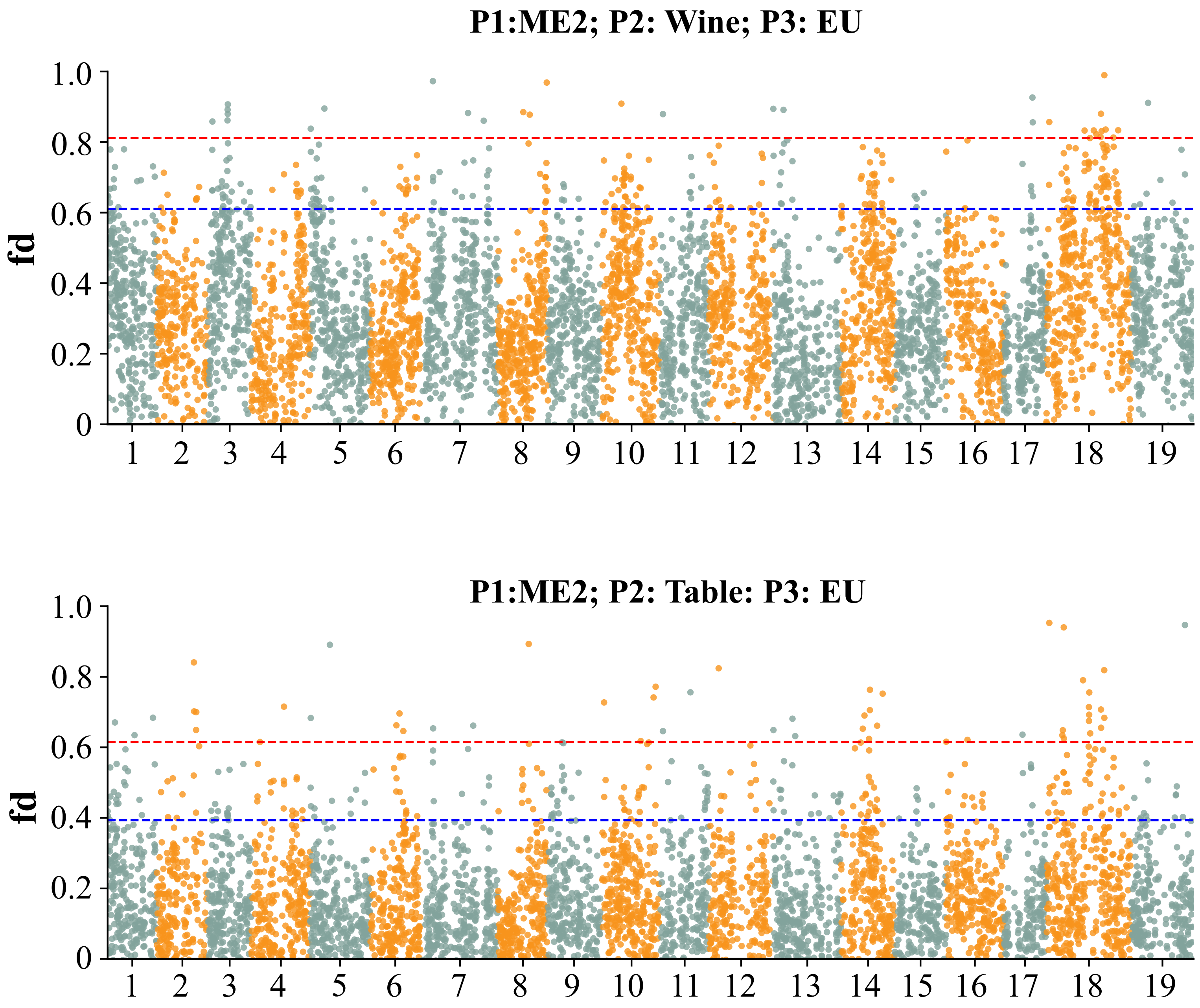

Figure S21. fd test between wine and EU group, table and EU group by 50 kb windows. The dots above the red horizontal line are the highest 1% regions, and the blue horizontal line indicate the threshold of 5% highest regions.

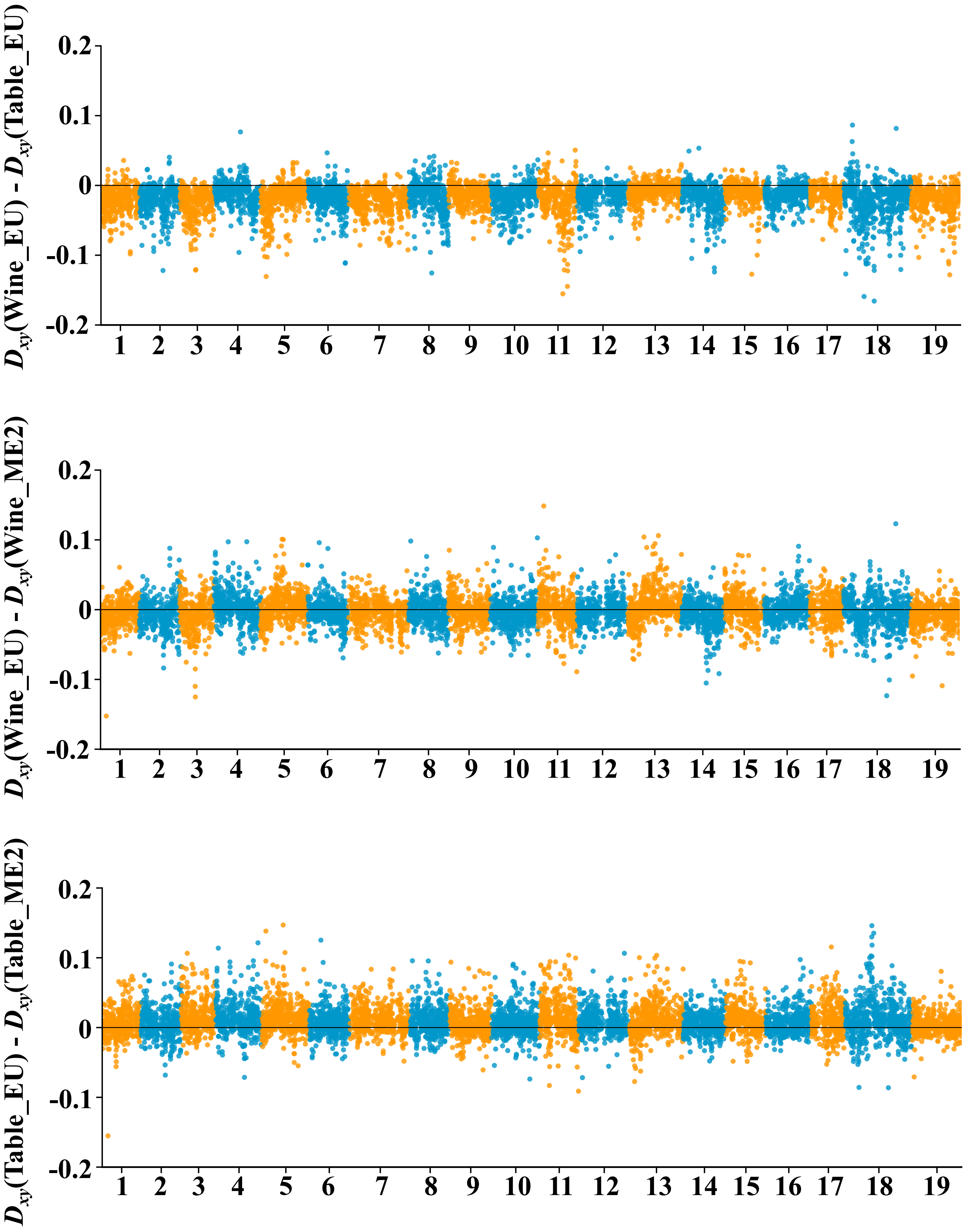

Figure S22. Differential Sequences similarity (D_{xy}) calculated between diffenent group combinations.

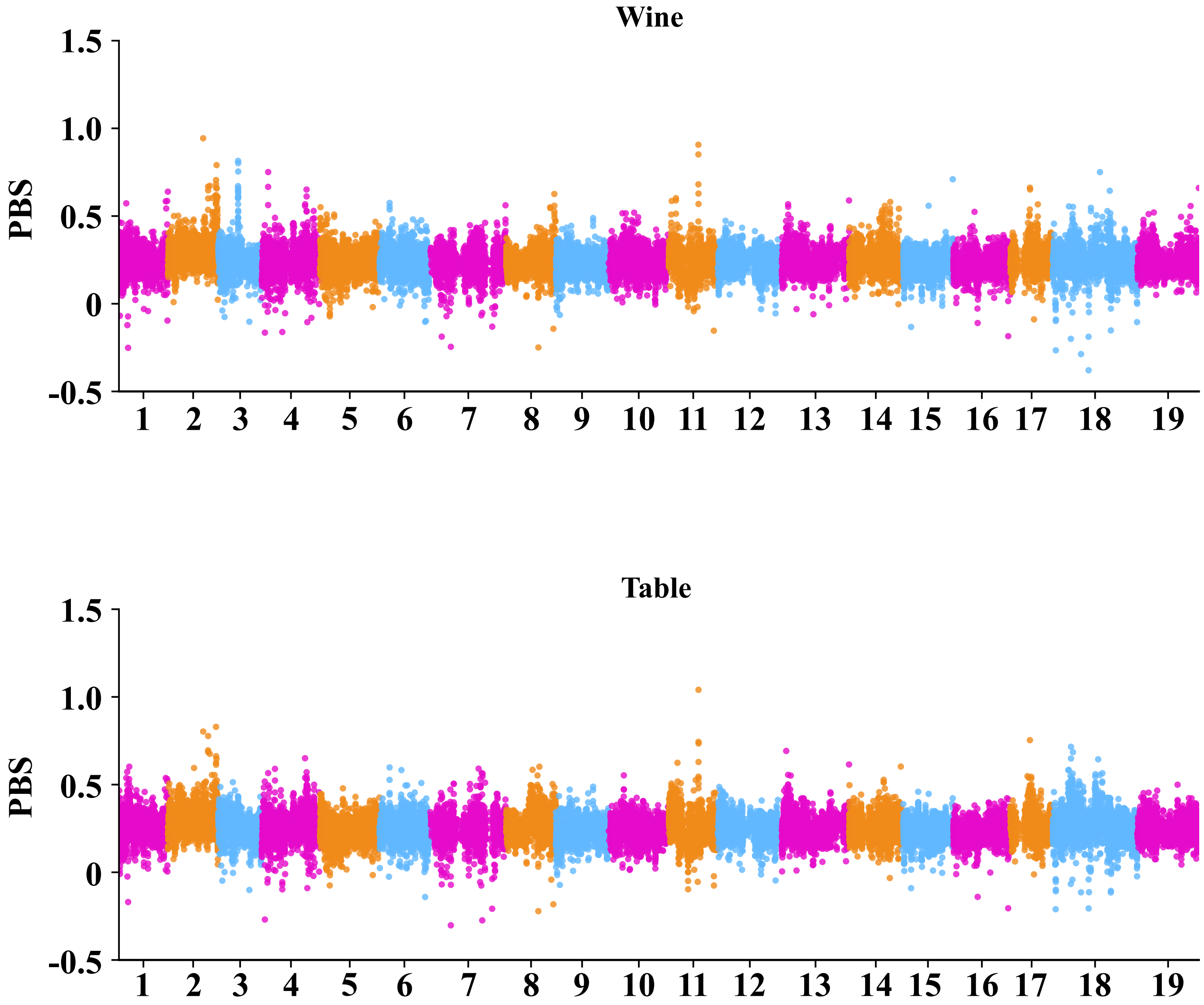

Figure S23. The population branch statistic (PBS) of domesticated grapes. PBS was calculated by comparing wine or table group with ME2 group and outgroup.

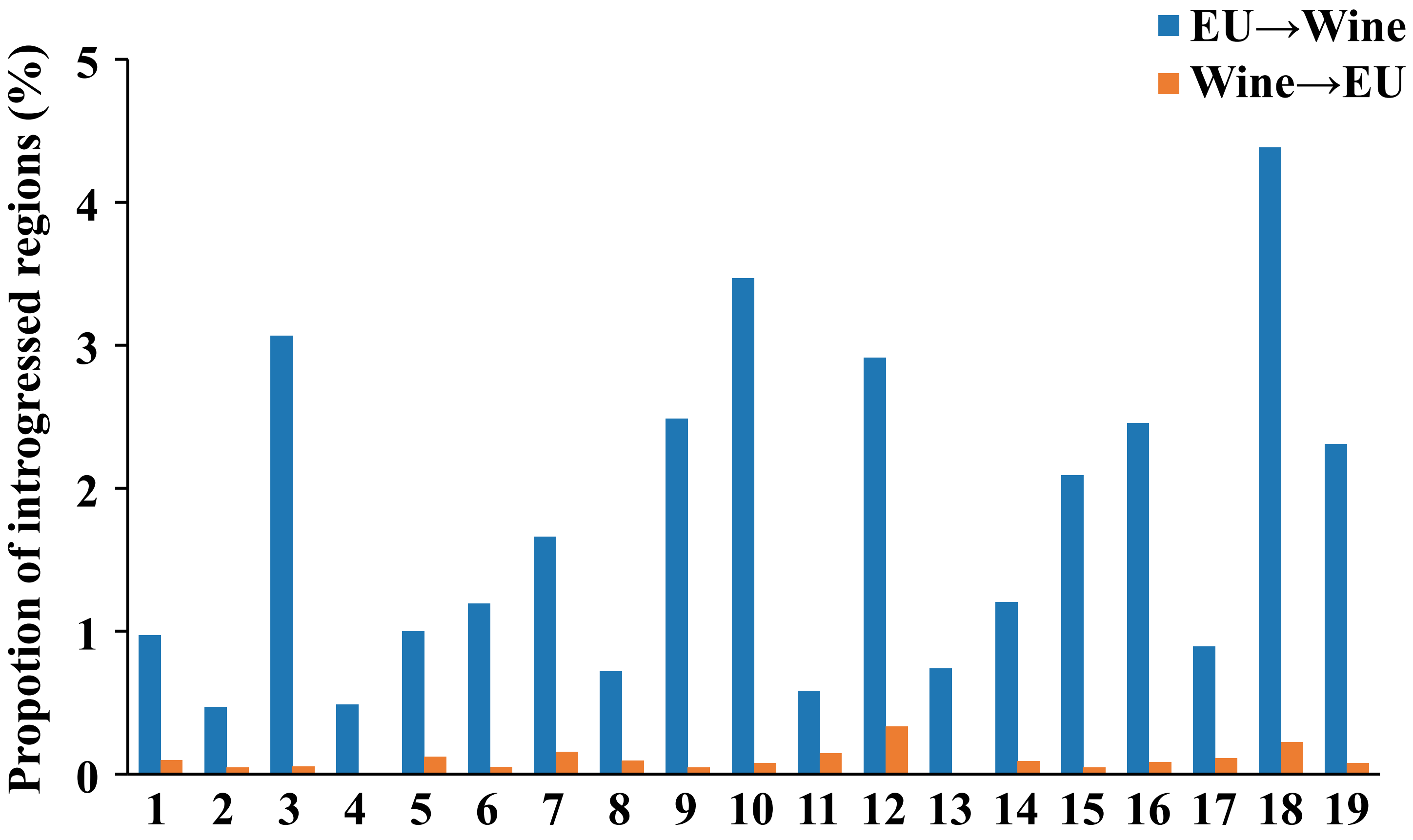

Figure S24. Proportion of predicted introgressed regions on each chromosome predicted by Filet. The blue bars represent the introgression from EU to wine grapes, the red bars represent the introgression from wine to EU grapes.

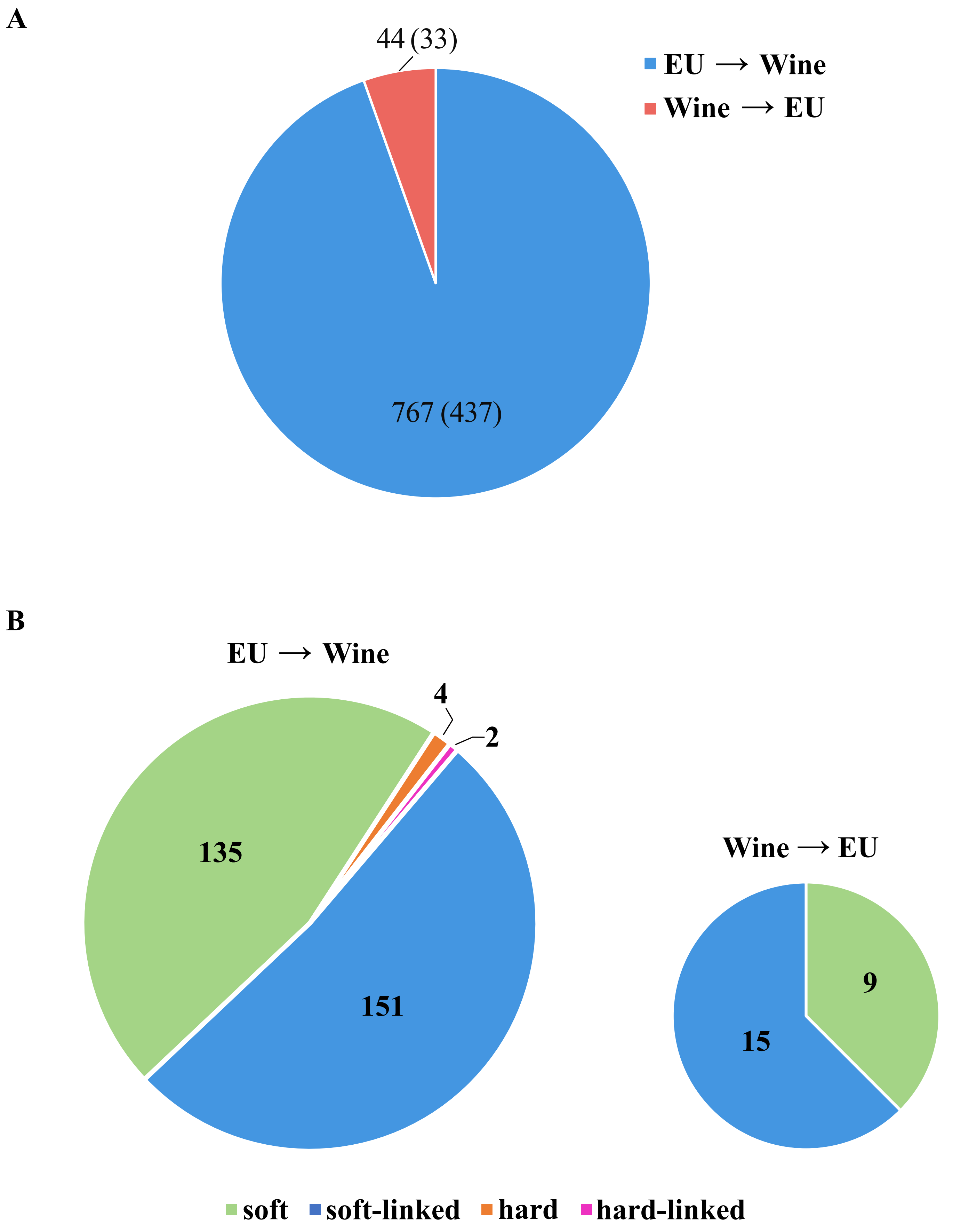

Figure S25. The genes located in the loci predicted as introgressed and sweep selected using machine learning. (A) Introgression from EU to wine (blue background) or from wine to EU (red background). The values outside the brackets are the number of genes that have been predicted. The values in the brackets are the number of genes that have been annotated. (B) The proportion of different kinds of selection types of these genes. The values are the number of genes in each category.

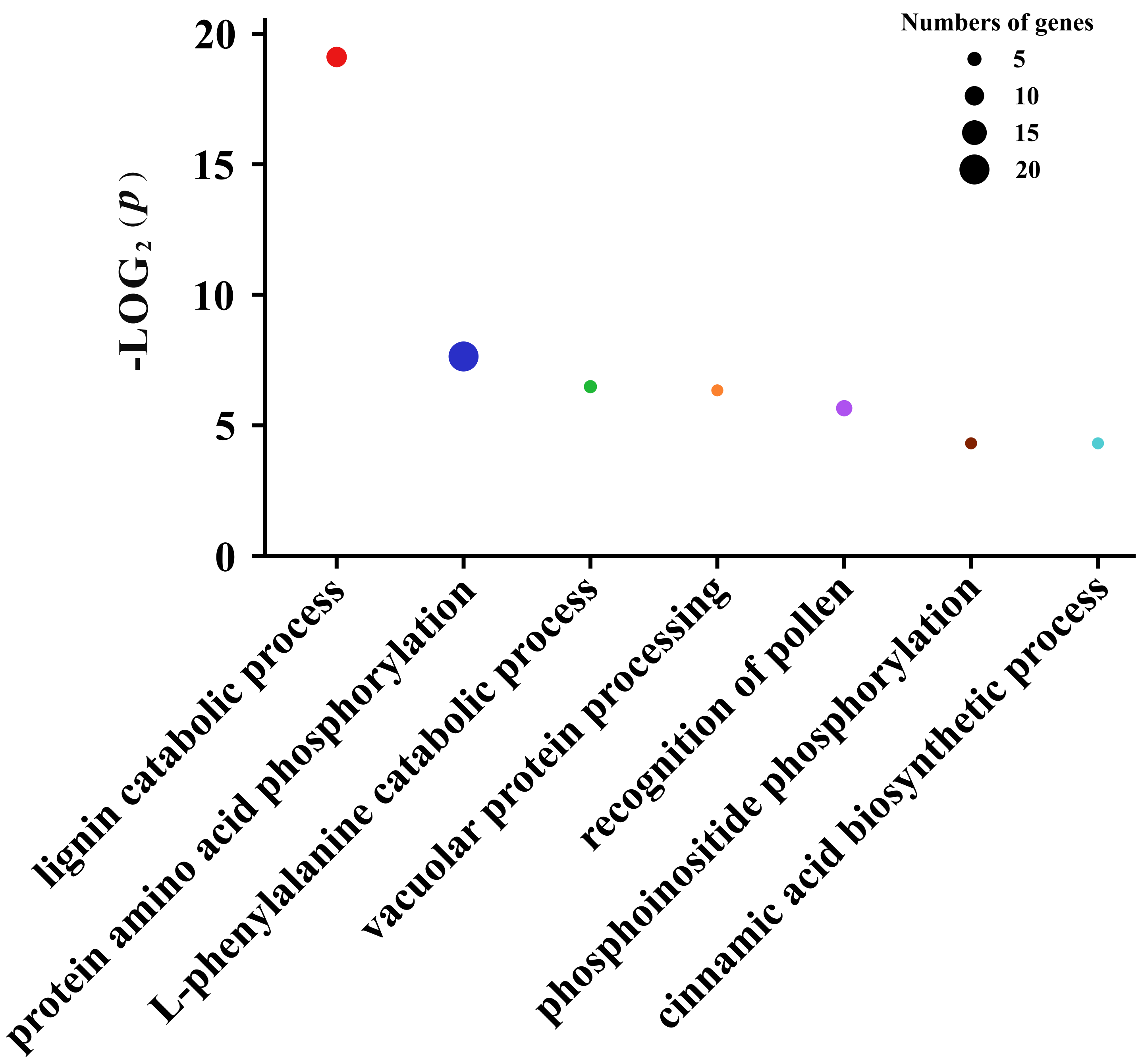

Figure S26. The significantly enriched GO terms in introgressed regions. The size of the circle indicates the number of genes in each process.

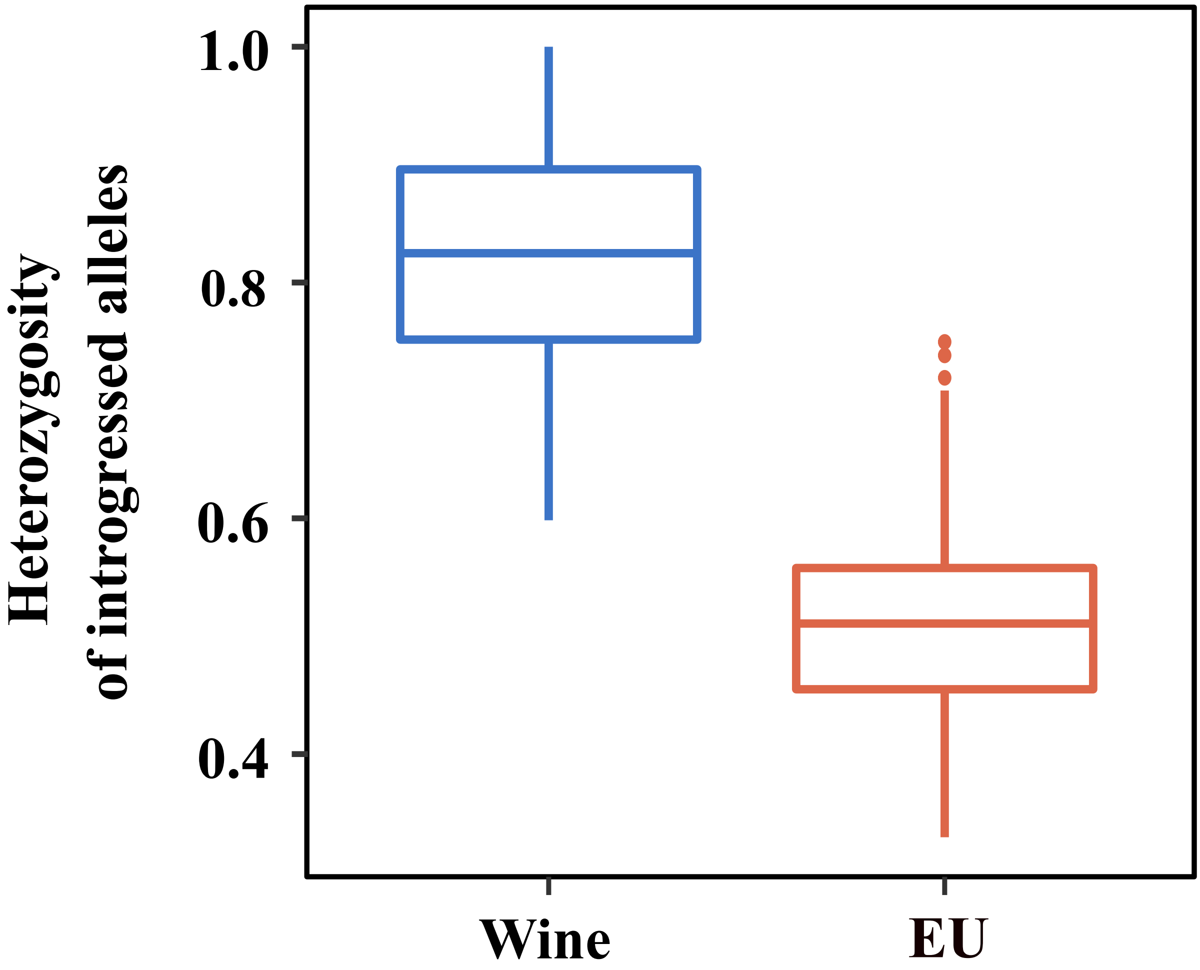

Figure S27. The heterozygosity of alleles that predicated as introgressed in wine group in each individual in wine and EU groups**.**

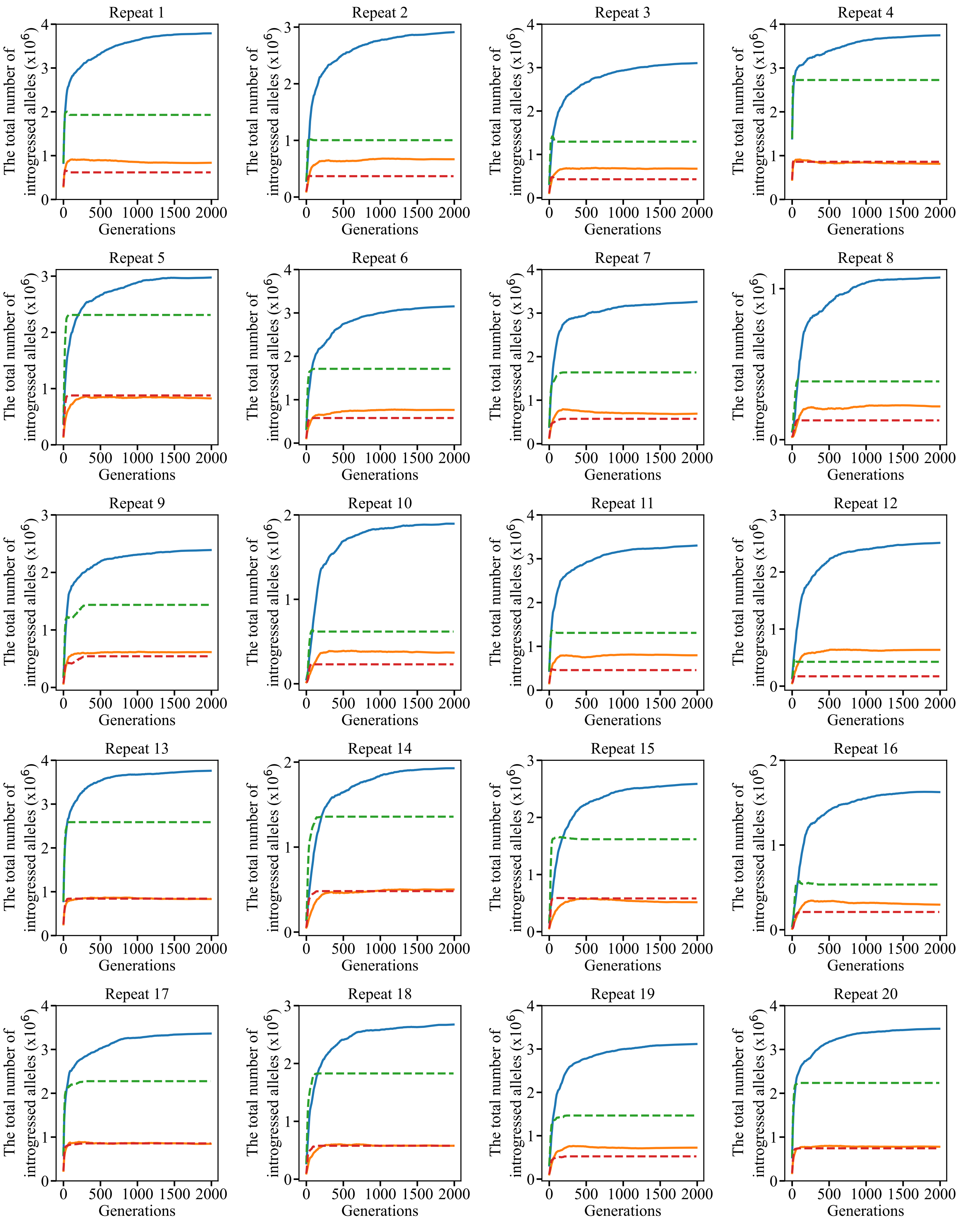

Figure S28. Estimation of the change of the total number of introgressed alleles in whole introgressed population after introgression using forwrd similation by slim. 20 repeats have been done and showed. The green and red bars indicate beneficial and deleterious alleles separately. The solid lines indicate the simulation in outcrossing population, and the dash lines indicate the simulation in clonal population. Blue and green lines indicate benificial alleles, and orange and red lines indicate deleterious alleles.

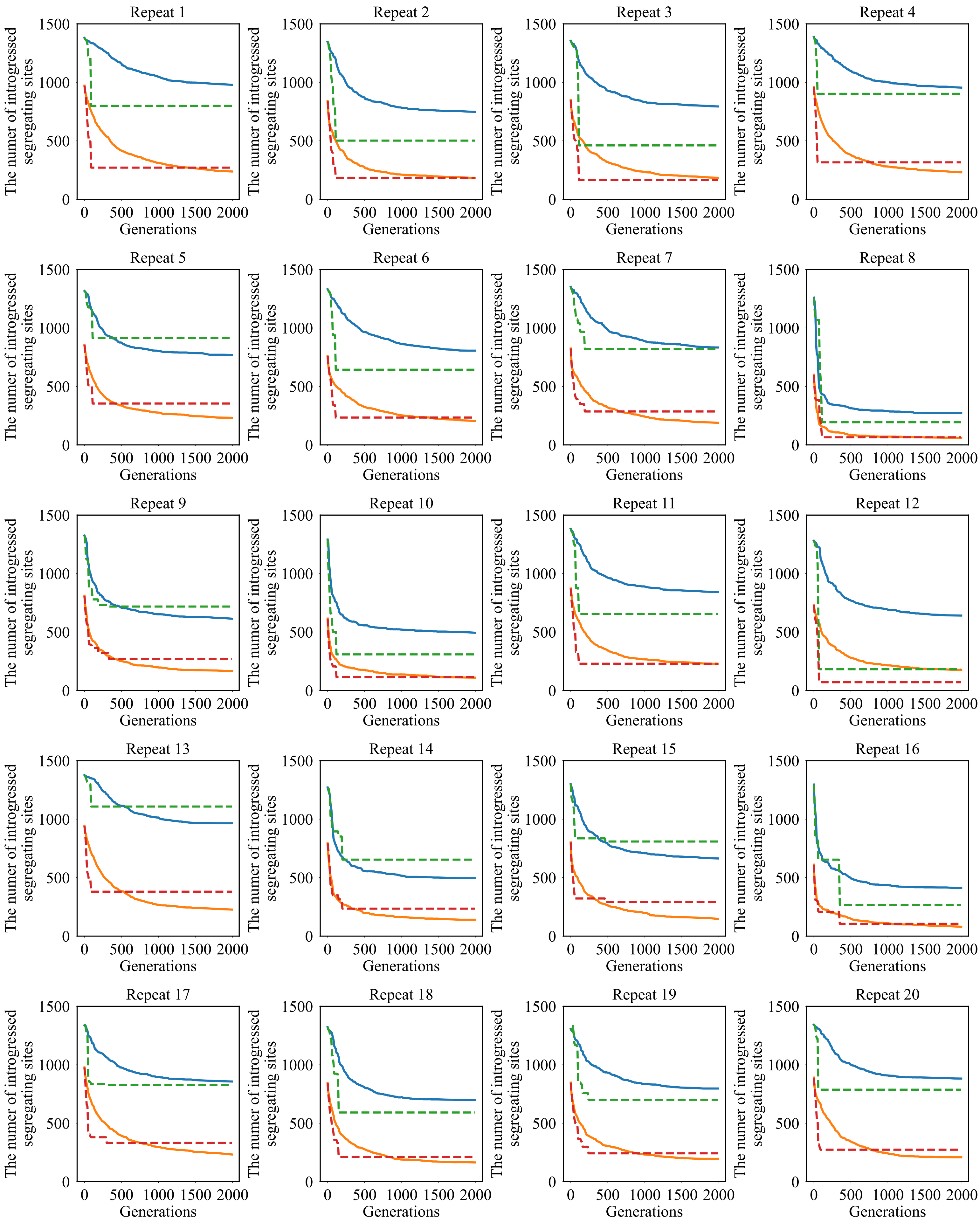

Figure S29. Estimation of the change of the number of introgressed segregating alleles in whole introgressed population after introgression using forwrd similation by slim. 20 repeats have been done and showed. The green and red bars indicate beneficial and deleterious alleles separately. The solid lines indicate the simulation in outcrossing population, and the dash lines indicate the simulation in clonal population. Blue and green lines indicate benificial alleles, and orange and red lines indicate deleterious alleles.

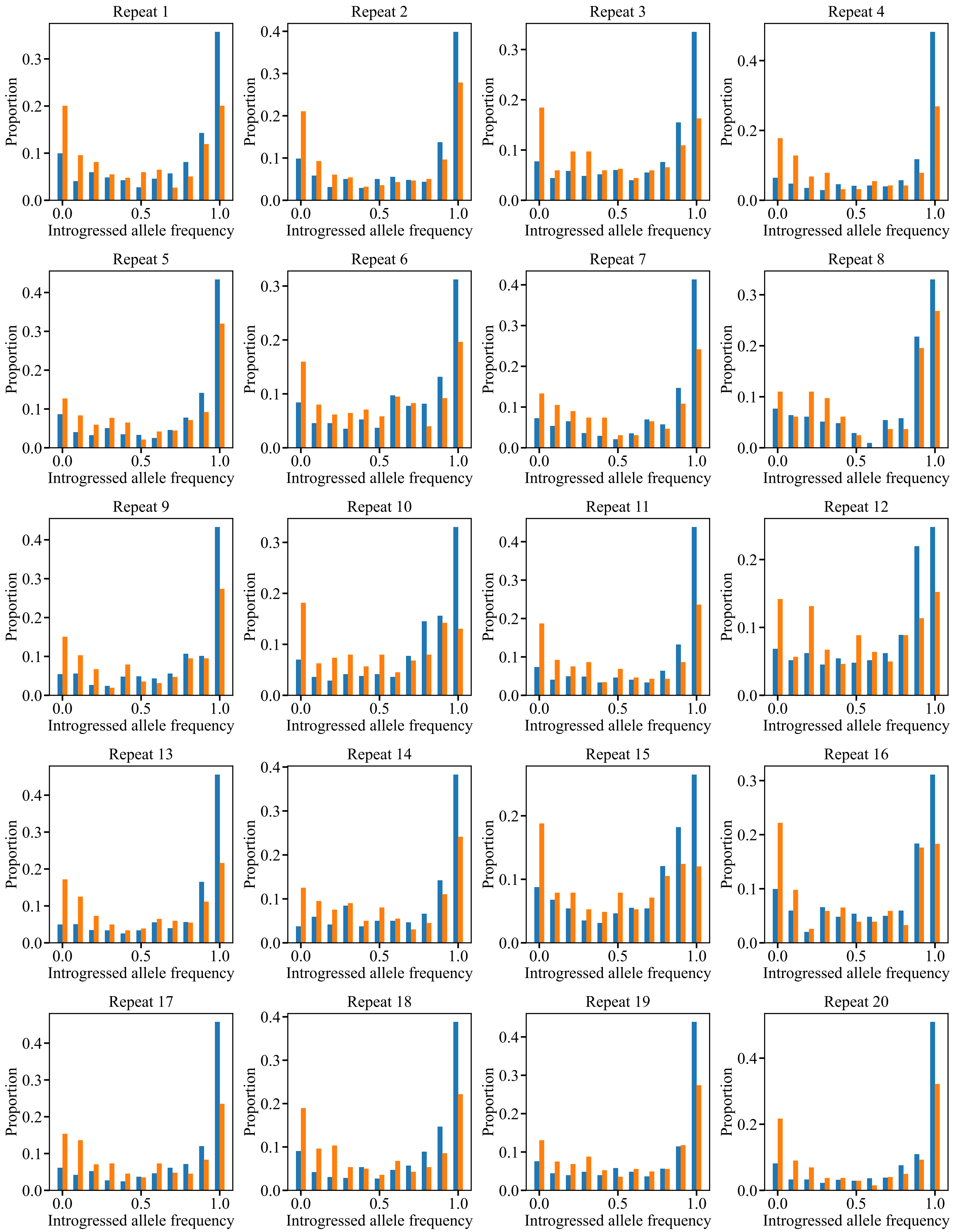

Figure S30. The estimation of the SFS of introgressed beneficial and deleterious alleles at the 500th generation after introgression in outcrosing population using forwrd similation by slim. 20 repeats have been done and showed. The blue and orange bars indicate beneficial and deleterious alleles separately.

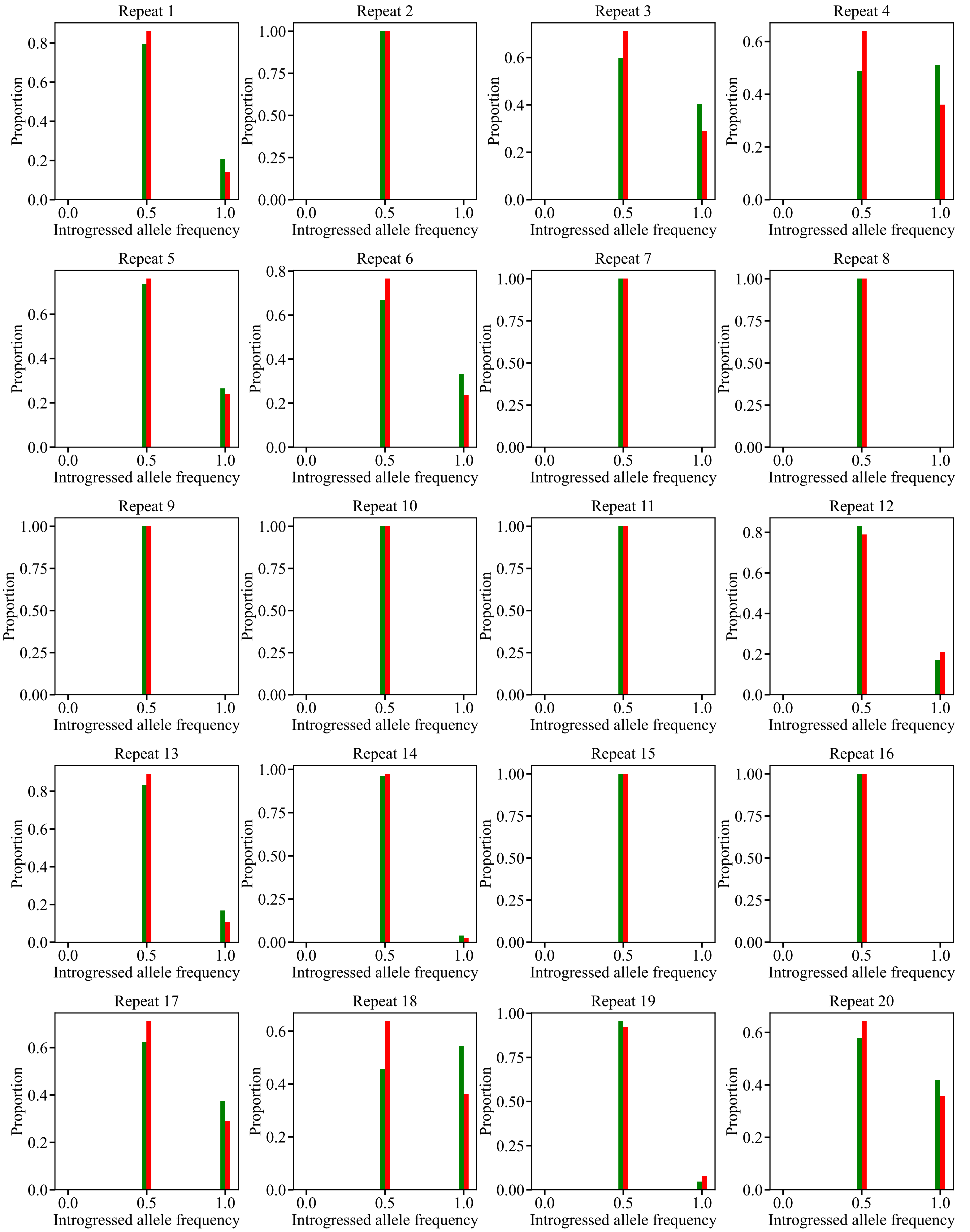

Figure S31. The estimation of the SFS of introgressed beneficial and deleterious alleles at the 500th generation after introgression in clonal population using forwrd similation by slim. 20 repeats have been done and showed. The green and red bars indicate beneficial and deleterious alleles separately.

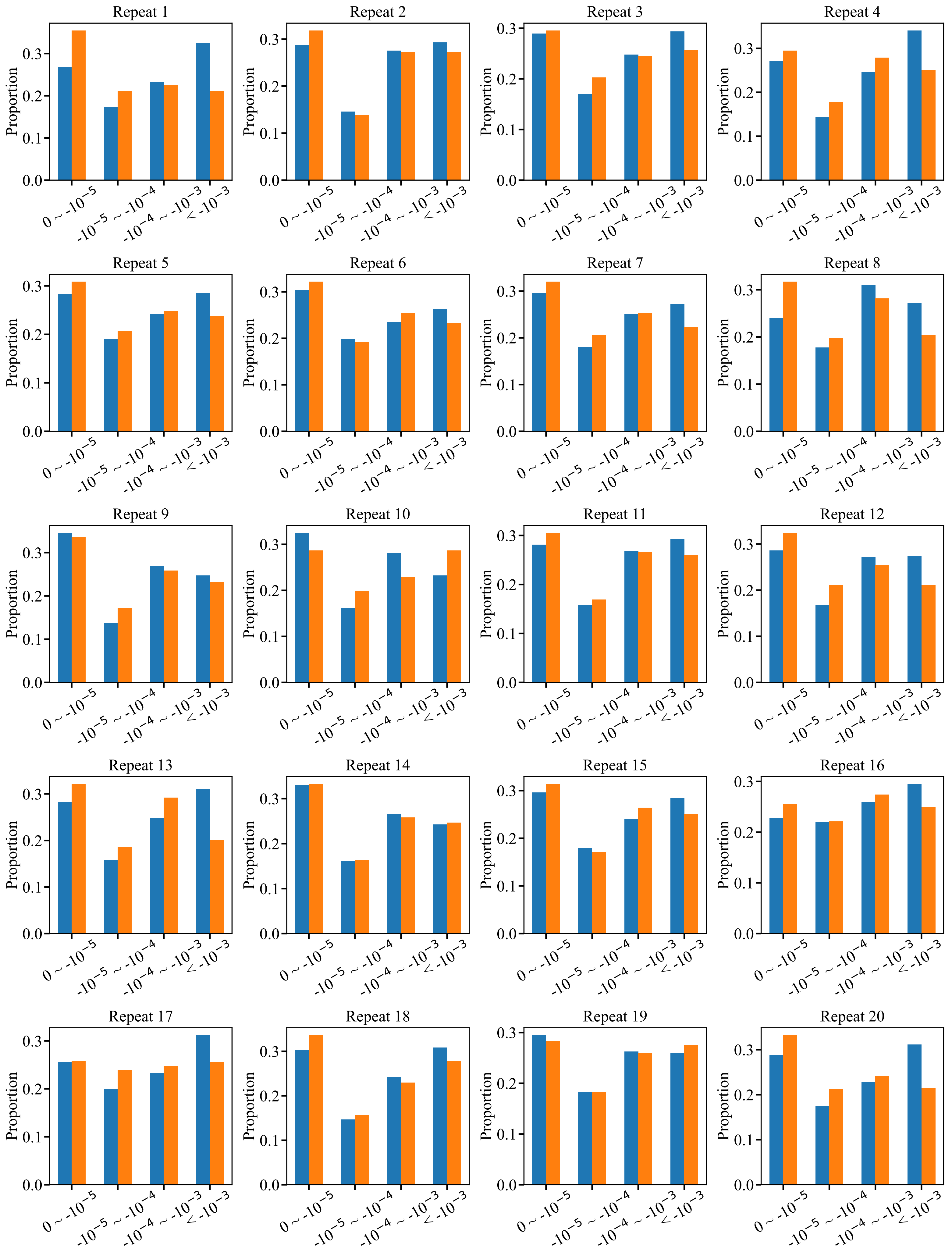

Figure S32. The DFE distribution of introgressed alleles in outcrossing and clonal populations at the 100th generation after introgression. The blue bars indicate outcrossing population and the orange bars indicate clonal population.

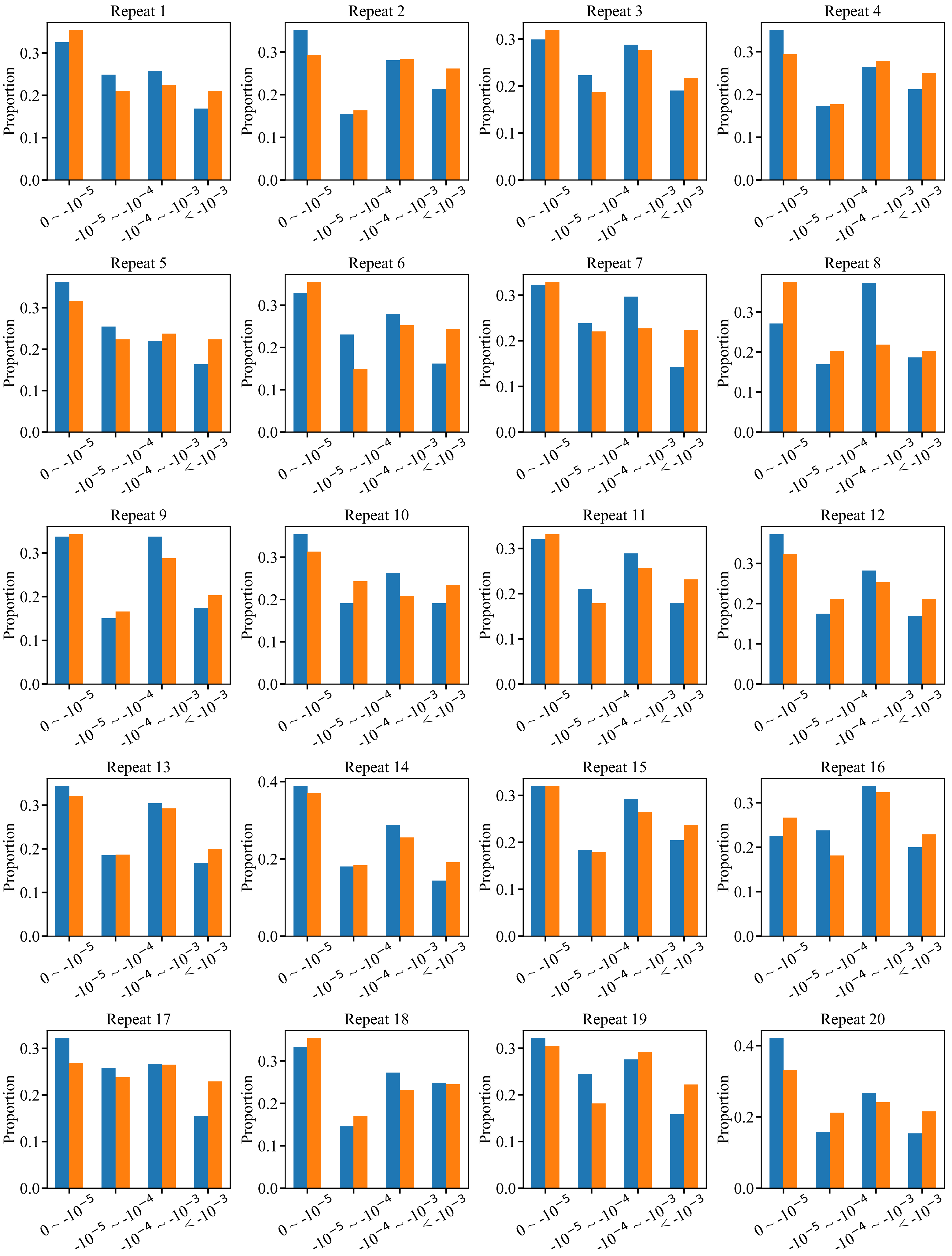

Figure S33. The DFE distribution of introgressed alleles in outcrossing and clonal populations at the 2000th generation after introgression. The blue bars indicate outcrossing population and the orange bars indicate clonal population.

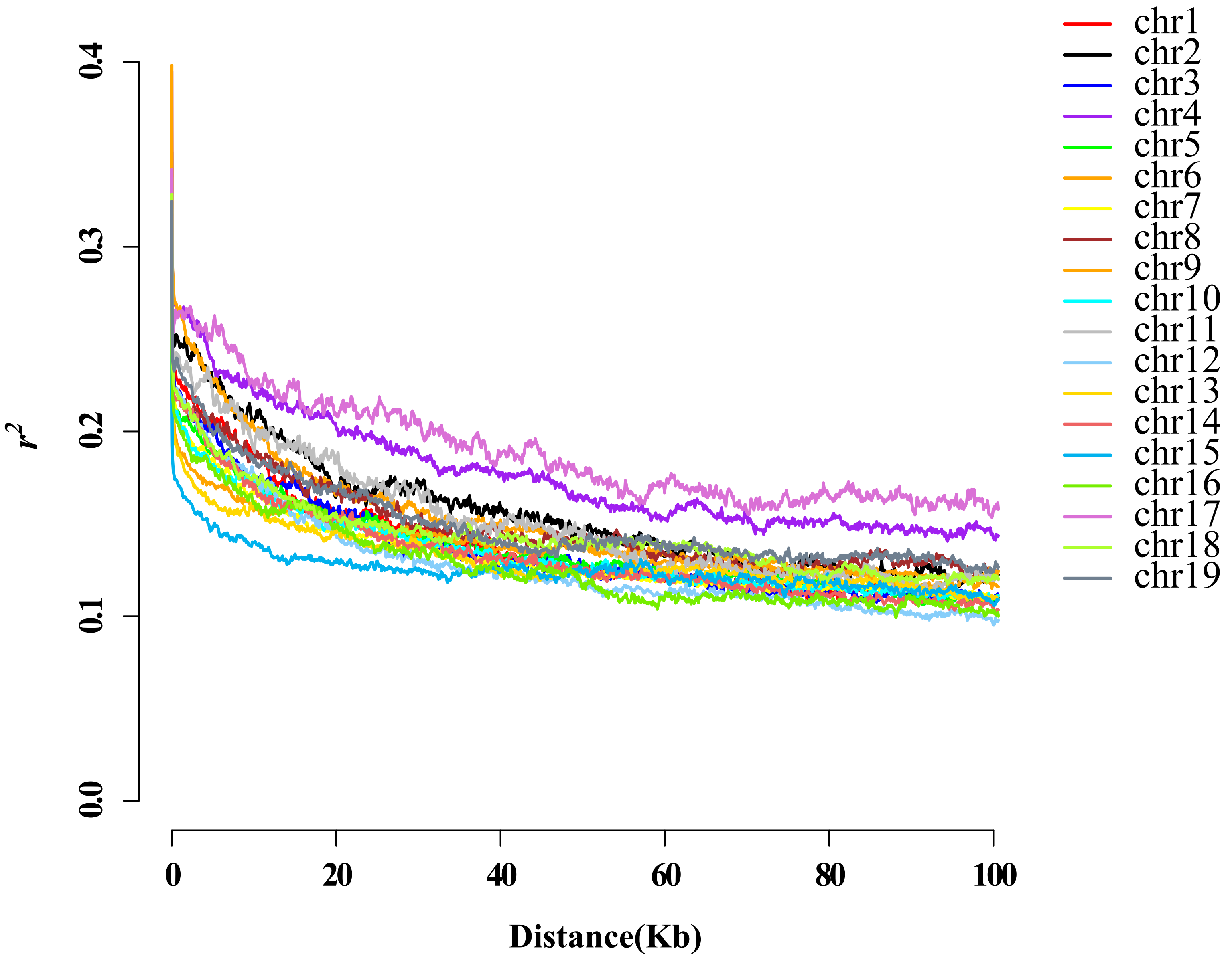

Figure S34. LD decay of each chromosome in wine population.

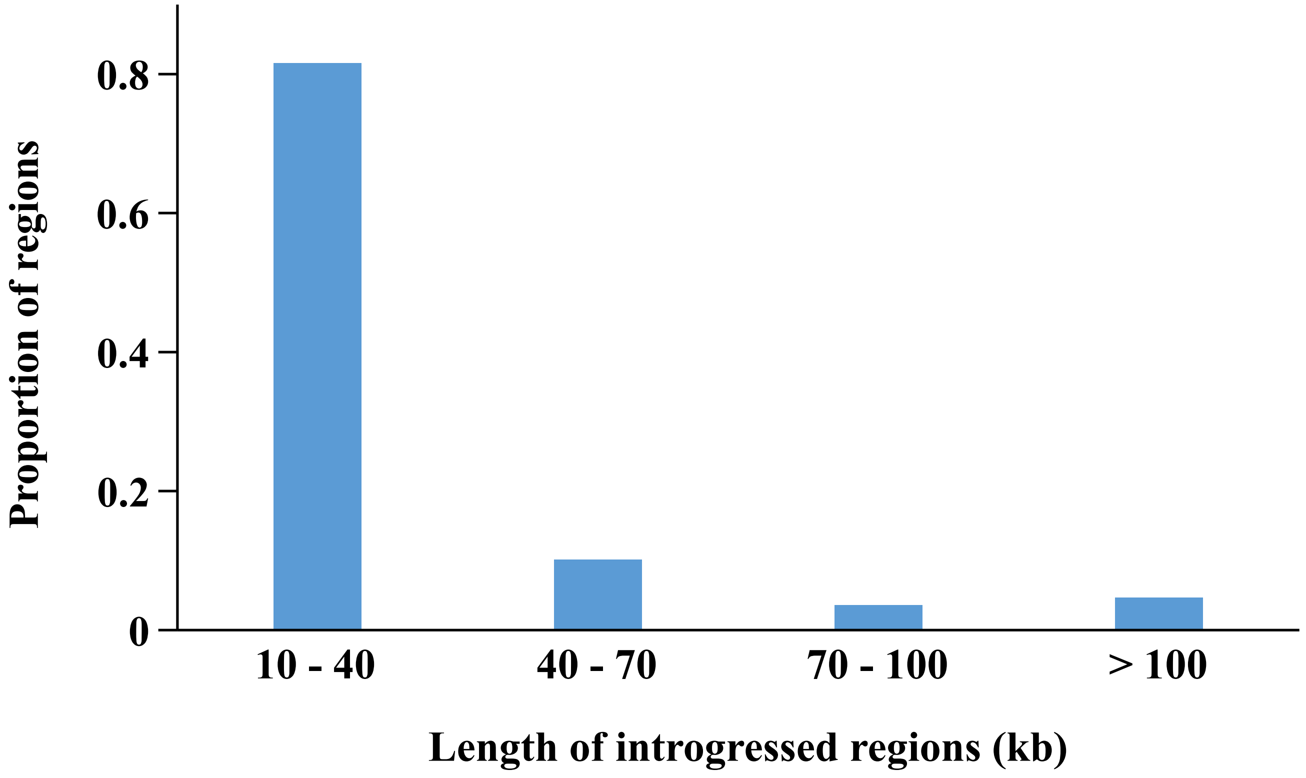

Figure S35. The distribution of the length of introgressed regions.The predicted adjacent 10kb introgressed regions were merged with the tolerance of 50 kb intervals.

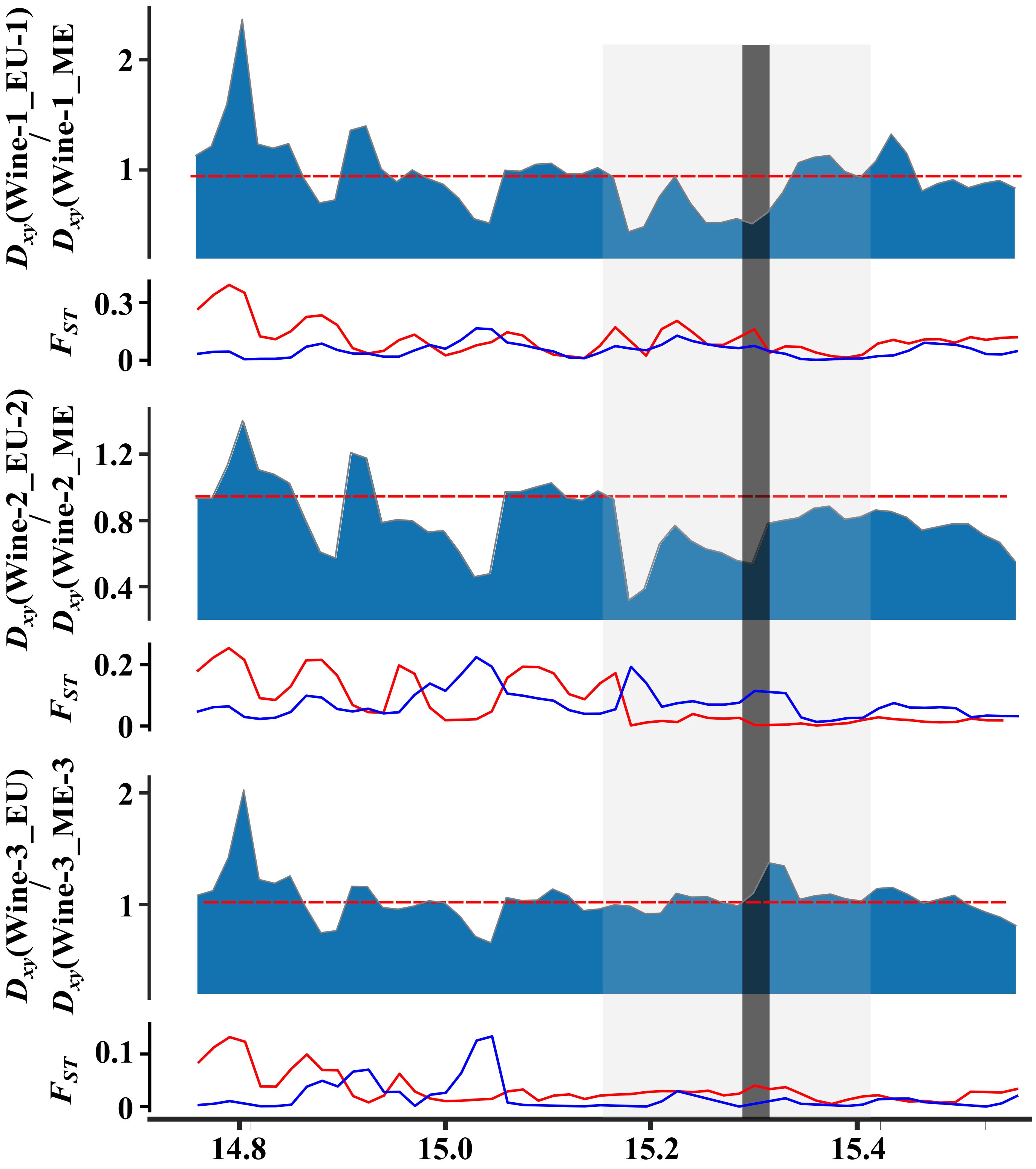

Figure S36. The D_{xy} and F_{ST} around the region containing the *Fer*-like gene that was predicted introgressed from EU and under selected. The line charts with blue background represent the D_{xy} between wine grapes and EU grapes divided by the D_{xy} between wine grapes and ME grapes in each subgroup. The horizontal red lines indicate the genome mean. The red broken lines indicate the F_{ST} between wine grapes and EU grapes and the blue broken lines indicate the F_{ST} between wine grapes and ME grapes. The black bar across all charts represents the region we focused.

Table S1. 345 accessions for analysis.

ID	Species	Cultivar	Group	Note
ERR4011039	*Vitis vinifera* ssp. *sylvestris*	wild grape	EU	
ERR4011040	*Vitis vinifera* ssp. *sylvestris*	wild grape	EU	
ERR4028632	*Vitis vinifera* ssp. *sylvestris*	wild grape	EU	
SRR5891605	*Vitis vinifera* ssp. *sylvestris*	wild grape	EU	
SRR5891606	*Vitis vinifera* ssp. *sylvestris*	wild grape	EU	
SRR5891608	*Vitis vinifera* ssp. *sylvestris*	wild grape	EU	
SRR5891609	*Vitis vinifera* ssp. *sylvestris*	wild grape	EU	
SRR5891610	*Vitis vinifera* ssp. *sylvestris*	wild grape	EU	
SRR5891611	*Vitis vinifera* ssp. *sylvestris*	wild grape	EU	
SRR5891612	*Vitis vinifera* ssp. *sylvestris*	wild grape	EU	
SRR5891613	*Vitis vinifera* ssp. *sylvestris*	wild grape	EU	
SRR5891614	*Vitis vinifera* ssp. *sylvestris*	wild grape	EU	
SRR5891676	*Vitis vinifera* ssp. *sylvestris*	wild grape	EU	
SRR5891678	*Vitis vinifera* ssp. *sylvestris*	wild grape	EU	
SRR5891679	*Vitis vinifera* ssp. *sylvestris*	wild grape	EU	
SRR5891702	*Vitis vinifera* ssp. *sylvestris*	wild grape	EU	
SRR5891703	*Vitis vinifera* ssp. *sylvestris*	wild grape	EU	
SRR5891704	*Vitis vinifera* ssp. *sylvestris*	wild grape	EU	
SRR5891705	*Vitis vinifera* ssp. *sylvestris*	wild grape	EU	
SRR5891706	*Vitis vinifera* ssp. *sylvestris*	wild grape	EU	
SRR5891707	*Vitis vinifera* ssp. *sylvestris*	wild grape	EU	
SRR5891708	*Vitis vinifera* ssp. *sylvestris*	wild grape	EU	
SRR5891709	*Vitis vinifera* ssp. *sylvestris*	wild grape	EU	
SRR5891710	*Vitis vinifera* ssp. *sylvestris*	wild grape	EU	
SRR5891711	*Vitis vinifera* ssp. *sylvestris*	wild grape	EU	
SRR5891712	*Vitis vinifera* ssp. *sylvestris*	wild grape	EU	
SRR5891713	*Vitis vinifera* ssp. *sylvestris*	wild grape	EU	
SRR5891714	*Vitis vinifera* ssp. *sylvestris*	wild grape	EU	
SRR5891715	*Vitis vinifera* ssp. *sylvestris*	wild grape	EU	
SRR5891716	*Vitis vinifera* ssp. *sylvestris*	wild grape	EU	
SRR5891717	*Vitis vinifera* ssp. *sylvestris*	wild grape	EU	
SRR5891718	*Vitis vinifera* ssp. *sylvestris*	wild grape	EU	
SRR5891719	*Vitis vinifera* ssp. *sylvestris*	wild grape	EU	
SRR5891720	*Vitis vinifera* ssp. *sylvestris*	wild grape	EU	
SRR5891721	*Vitis vinifera* ssp. *sylvestris*	wild grape	EU	
SRR5891759	*Vitis vinifera* ssp. *sylvestris*	wild grape	EU	
SRR5891760	*Vitis vinifera* ssp. *sylvestris*	wild grape	EU	
SRR5891761	*Vitis vinifera* ssp. *sylvestris*	wild grape	EU	
SRR5891762	*Vitis vinifera* ssp. *sylvestris*	wild grape	EU	
SRR5891763	*Vitis vinifera* ssp. *sylvestris*	wild grape	EU	
SRR5891764	*Vitis vinifera* ssp. *sylvestris*	wild grape	EU	

SRR5891765	*Vitis vinifera* ssp. *sylvestris*	wild grape	EU	
SRR5891766	*Vitis vinifera* ssp. *sylvestris*	wild grape	EU	
SRR5891768	*Vitis vinifera* ssp. *sylvestris*	wild grape	EU	
SRR5891874	*Vitis vinifera* ssp. *sylvestris*	wild grape	EU	
SRR5891875	*Vitis vinifera* ssp. *sylvestris*	wild grape	EU	
SRR5891876	*Vitis vinifera* ssp. *sylvestris*	wild grape	EU	
SRR5891877	*Vitis vinifera* ssp. *sylvestris*	wild grape	EU	
SRR5891878	*Vitis vinifera* ssp. *sylvestris*	wild grape	EU	
SRR5891879	*Vitis vinifera* ssp. *sylvestris*	wild grape	EU	
SRR5891880	*Vitis vinifera* ssp. *sylvestris*	wild grape	EU	
SRR5891881	*Vitis vinifera* ssp. *sylvestris*	wild grape	EU	
SRR5891882	*Vitis vinifera* ssp. *sylvestris*	wild grape	EU	
SRR5891883	*Vitis vinifera* ssp. *sylvestris*	wild grape	EU	
SRR5891934	*Vitis vinifera* ssp. *sylvestris*	wild grape	EU	
SRR5891935	*Vitis vinifera* ssp. *sylvestris*	wild grape	EU	
SRR5891937	*Vitis vinifera* ssp. *sylvestris*	wild grape	EU	
SRR5891938	*Vitis vinifera* ssp. *sylvestris*	wild grape	EU	
SRR5891939	*Vitis vinifera* ssp. *sylvestris*	wild grape	EU	
SRR5891940	*Vitis vinifera* ssp. *sylvestris*	wild grape	EU	
SRR5891941	*Vitis vinifera* ssp. *sylvestris*	wild grape	EU	
SRR5891942	*Vitis vinifera* ssp. *sylvestris*	wild grape	EU	
SRR5891943	*Vitis vinifera* ssp. *sylvestris*	wild grape	EU	
SRR8835138	*Vitis vinifera* ssp. *sylvestris*	wild grape	EU	
SRR8835141	*Vitis vinifera* ssp. *sylvestris*	wild grape	EU	
SRR8835162	*Vitis vinifera* ssp. *sylvestris*	wild grape	EU	
SRR8835164	*Vitis vinifera* ssp. *sylvestris*	wild grape	EU	
SRR9591081	*Vitis vinifera* ssp. *sylvestris*	wild grape	EU	
SRR9591082	*Vitis vinifera* ssp. *sylvestris*	wild grape	EU	
P50	*Vitis vinifera* ssp. *sylvestris*	wild grape	EU	Sequenced in this study
P51	*Vitis vinifera* ssp. *sylvestris*	wild grape	EU	Sequenced in this study
P53	*Vitis vinifera* ssp. *sylvestris*	wild grape	EU	Sequenced in this study
SRR5627784	*Vitis vinifera* ssp. *sylvestris*	wild grape	ME1	
SRR5627786	*Vitis vinifera* ssp. *sylvestris*	wild grape	ME1	
SRR5627787	*Vitis vinifera* ssp. *sylvestris*	wild grape	ME1	
SRR5627789	*Vitis vinifera* ssp. *sylvestris*	wild grape	ME1	
SRR5627790	*Vitis vinifera* ssp. *sylvestris*	wild grape	ME1	
P54	*Vitis vinifera* ssp. *sylvestris*	wild grape	ME1	Sequenced in this study
P55	*Vitis vinifera* ssp. *sylvestris*	wild grape	ME1	Sequenced in this study
P56	*Vitis vinifera* ssp. *sylvestris*	wild grape	ME1	Sequenced in this study
P61	*Vitis vinifera* ssp. *sylvestris*	wild grape	ME1	Sequenced in this study
P62	*Vitis vinifera* ssp. *sylvestris*	wild grape	ME1	Sequenced in this study
P63	*Vitis vinifera* ssp. *sylvestris*	wild grape	ME1	Sequenced in this study
P64	*Vitis vinifera* ssp. *sylvestris*	wild grape	ME1	Sequenced in this study

P65	*Vitis vinifera* ssp. *sylvestris*	wild grape	ME1	Sequenced in this study
P66	*Vitis vinifera* ssp. *sylvestris*	wild grape	ME1	Sequenced in this study
P67	*Vitis vinifera* ssp. *sylvestris*	wild grape	ME1	Sequenced in this study
P69	*Vitis vinifera* ssp. *sylvestris*	wild grape	ME1	Sequenced in this study
P73	*Vitis vinifera* ssp. *sylvestris*	wild grape	ME1	Sequenced in this study
P74	*Vitis vinifera* ssp. *sylvestris*	wild grape	ME1	Sequenced in this study
P77	*Vitis vinifera* ssp. *sylvestris*	wild grape	ME1	Sequenced in this study
P78	*Vitis vinifera* ssp. *sylvestris*	wild grape	ME1	Sequenced in this study
P79	*Vitis vinifera* ssp. *sylvestris*	wild grape	ME1	Sequenced in this study
P80	*Vitis vinifera* ssp. *sylvestris*	wild grape	ME1	Sequenced in this study
P81	*Vitis vinifera* ssp. *sylvestris*	wild grape	ME1	Sequenced in this study
P86	*Vitis vinifera* ssp. *sylvestris*	wild grape	ME1	Sequenced in this study
P87	*Vitis vinifera* ssp. *sylvestris*	wild grape	ME1	Sequenced in this study
P88	*Vitis vinifera* ssp. *sylvestris*	wild grape	ME1	Sequenced in this study
P89	*Vitis vinifera* ssp. *sylvestris*	wild grape	ME1	Sequenced in this study
P90	*Vitis vinifera* ssp. *sylvestris*	wild grape	ME1	Sequenced in this study
P91	*Vitis vinifera* ssp. *sylvestris*	wild grape	ME1	Sequenced in this study
SRR12265757	*Vitis vinifera* ssp. *sylvestris*	wild grape	ME2	
SRR12265763	*Vitis vinifera* ssp. *sylvestris*	wild grape	ME2	
SRR12265766	*Vitis vinifera* ssp. *sylvestris*	wild grape	ME2	
SRR12265776	*Vitis vinifera* ssp. *sylvestris*	wild grape	ME2	
SRR12265779	*Vitis vinifera* ssp. *sylvestris*	wild grape	ME2	
SRR12265781	*Vitis vinifera* ssp. *sylvestris*	wild grape	ME2	
SRR12265783	*Vitis vinifera* ssp. *sylvestris*	wild grape	ME2	
SRR094945	*Vitis rotundifolia*	wild grape	outgroup	
SRR6729330	*Vitis rotundifolia*	wild grape	outgroup	
SRR6729331	*Vitis rotundifolia*	wild grape	outgroup	
4R112-L4	*Vitis californica*	wild grape	outgroup	Sequenced in this study
4R112-L5	*Vitis californica*	wild grape	outgroup	Sequenced in this study
4R112-L6	*Vitis californica*	wild grape	outgroup	Sequenced in this study
SRR10439055	*Vitis vinifera* ssp. *vinifera*	Muscat Hamburg	table	
SRR12265743	*Vitis vinifera* ssp. *vinifera*	Beer	table	
SRR12265744	*Vitis vinifera* ssp. *vinifera*	Zuba-A	table	
SRR12265745	*Vitis vinifera* ssp. *vinifera*	Zeni-A	table	
SRR12265746	*Vitis vinifera* ssp. *vinifera*	Zeituni	table	
SRR12265747	*Vitis vinifera* ssp. *vinifera*	Yakim	table	
SRR12265750	*Vitis vinifera* ssp. *vinifera*	Tanur	table	
SRR12265752	*Vitis vinifera* ssp. *vinifera*	Tamar-H1	table	
SRR12265753	*Vitis vinifera* ssp. *vinifera*	Suriman	table	
SRR12265754	*Vitis vinifera* ssp. *vinifera*	Suka	table	
SRR12265755	*Vitis vinifera* ssp. *vinifera*	Sikusi	table	
SRR12265756	*Vitis vinifera* ssp. *vinifera*	Shami	table	
SRR12265758	*Vitis vinifera* ssp. *vinifera*	Safsufa-W	table	

SRR12265759	*Vitis vinifera* ssp. *vinifera*	Safsufa-R	table	
SRR12265760	*Vitis vinifera* ssp. *vinifera*	Baluti	table	
SRR12265761	*Vitis vinifera* ssp. *vinifera*	Rumi	table	
SRR12265764	*Vitis vinifera* ssp. *vinifera*	Orcha	table	
SRR12265765	*Vitis vinifera* ssp. *vinifera*	Nizanim-P	table	
SRR12265767	*Vitis vinifera* ssp. *vinifera*	Matar	table	
SRR12265768	*Vitis vinifera* ssp. *vinifera*	Margaliot-1	table	
SRR12265772	*Vitis vinifera* ssp. *vinifera*	Marawani-GH	table	
SRR12265773	*Vitis vinifera* ssp. *vinifera*	Marawani	table	
SRR12265777	*Vitis vinifera* ssp. *vinifera*	Madawar	table	
SRR12265778	*Vitis vinifera* ssp. *vinifera*	Kursi	table	
SRR12265780	*Vitis vinifera* ssp. *vinifera*	Karkashani	table	
SRR12265782	*Vitis vinifera* ssp. *vinifera*	Ayarim1	table	
SRR12265784	*Vitis vinifera* ssp. *vinifera*	Jurdich	table	
SRR12265785	*Vitis vinifera* ssp. *vinifera*	Jandaly	table	
SRR12265786	*Vitis vinifera* ssp. *vinifera*	Homra	table	
SRR12265787	*Vitis vinifera* ssp. *vinifera*	Hilwani	table	
SRR12265788	*Vitis vinifera* ssp. *vinifera*	Hadari	table	
SRR12265789	*Vitis vinifera* ssp. *vinifera*	Gilboa	table	
SRR12265790	*Vitis vinifera* ssp. *vinifera*	El-Al	table	
SRR12265791	*Vitis vinifera* ssp. *vinifera*	Darwishi	table	
SRR12265793	*Vitis vinifera* ssp. *vinifera*	Asba-el-arus	table	
SRR12265794	*Vitis vinifera* ssp. *vinifera*	Amud	table	
SRR354201	*Vitis vinifera* ssp. *vinifera*	Red_Globe	table	
SRR5627781	*Vitis vinifera* ssp. *vinifera*	Muscat of Alexandria	table	
SRR5627782	*Vitis vinifera* ssp. *vinifera*	Thompson 2A	table	
SRR5712111	*Vitis vinifera* ssp. *vinifera*	Kishmish Vatkana b	table	
SRR5891603	*Vitis vinifera* ssp. *vinifera*	Misiheyi	table	
SRR5891604	*Vitis vinifera* ssp. *vinifera*	Rose Ciotat	table	
SRR5891615	*Vitis vinifera* ssp. *vinifera*	Ruby Okuyama	table	
SRR5891625	*Vitis vinifera* ssp. *vinifera*	Flame Seedless	table	
SRR5891628	*Vitis vinifera* ssp. *vinifera*	Centennial Seedless	table	
SRR5891629	*Vitis vinifera* ssp. *vinifera*	Christmas Rose	table	
SRR5891634	*Vitis vinifera* ssp. *vinifera*	Exotic	table	
SRR5891636	*Vitis vinifera* ssp. *vinifera*	Cannero	table	
SRR5891640	*Vitis vinifera* ssp. *vinifera*	Benibarao	table	
SRR5891641	*Vitis vinifera* ssp. *vinifera*	Black ballade (4N)	table	
SRR5891642	*Vitis vinifera* ssp. *vinifera*	Black Baladi	table	
SRR5891644	*Vitis vinifera* ssp. *vinifera*	Black Hamburg	table	
SRR5891655	*Vitis vinifera* ssp. *vinifera*	Pin10-3	table	
SRR5891681	*Vitis vinifera* ssp. *vinifera*	Khoussaine Kalim Barmak	table	
SRR5891682	*Vitis vinifera* ssp. *vinifera*	Zana	table	
SRR5891683	*Vitis vinifera* ssp. *vinifera*	Flame Tokay	table	

SRR5891686	*Vitis vinifera* ssp. *vinifera*	JingYu	table	
SRR5891687	*Vitis vinifera* ssp. *vinifera*	Jingyan	table	
SRR5891688	*Vitis vinifera* ssp. *vinifera*	Myxyaohu	table	
SRR5891689	*Vitis vinifera* ssp. *vinifera*	Jingzaojing	table	
SRR5891690	*Vitis vinifera* ssp. *vinifera*	Manai	table	
SRR5891691	*Vitis vinifera* ssp. *vinifera*	Lizixiang	table	
SRR5891692	*Vitis vinifera* ssp. *vinifera*	Xiangfei	table	
SRR5891693	*Vitis vinifera* ssp. *vinifera*	Pinger Putao	table	
SRR5891694	*Vitis vinifera* ssp. *vinifera*	Zaomanao	table	
SRR5891696	*Vitis vinifera* ssp. *vinifera*	Red Alissa	table	
SRR5891722	*Vitis vinifera* ssp. *vinifera*	Baiburuike	table	
SRR5891723	*Vitis vinifera* ssp. *vinifera*	Bailaohuyan	table	
SRR5891726	*Vitis vinifera* ssp. *vinifera*	Misket Dunavski	table	
SRR5891729	*Vitis vinifera* ssp. *vinifera*	Aishenmeigui	table	
SRR5891742	*Vitis vinifera* ssp. *vinifera*	Neo Muscat	table	
SRR5891743	*Vitis vinifera* ssp. *vinifera*	Minikaiji	table	
SRR5891744	*Vitis vinifera* ssp. *vinifera*	Manicure Finger	table	
SRR5891745	*Vitis vinifera* ssp. *vinifera*	Headdress	table	
SRR5891746	*Vitis vinifera* ssp. *vinifera*	Red Cocoon	table	
SRR5891749	*Vitis vinifera* ssp. *vinifera*	Unicorn	table	
SRR5891756	*Vitis vinifera* ssp. *vinifera*	Rosario Rosso	table	
SRR5891769	*Vitis vinifera* ssp. *vinifera*	Chasselas Rose	table	
SRR5891771	*Vitis vinifera* ssp. *vinifera*	Zexiang	table	
SRR5891782	*Vitis vinifera* ssp. *vinifera*	Blush Seedless	table	
SRR5891784	*Vitis vinifera* ssp. *vinifera*	Black Rose	table	
SRR5891785	*Vitis vinifera* ssp. *vinifera*	Black Monukka	table	
SRR5891786	*Vitis vinifera* ssp. *vinifera*	Beauty Seedless	table	
SRR5891791	*Vitis vinifera* ssp. *vinifera*	Fenhongtaifei	table	
SRR5891792	*Vitis vinifera* ssp. *vinifera*	Gapandmak	table	
SRR5891796	*Vitis vinifera* ssp. *vinifera*	Delameigui	table	
SRR5891803	*Vitis vinifera* ssp. *vinifera*	Hanpin21	table	
SRR5891821	*Vitis vinifera* ssp. *vinifera*	Heikalasi	table	
SRR5891823	*Vitis vinifera* ssp. *vinifera*	Guibao	table	
SRR5891824	*Vitis vinifera* ssp. *vinifera*	Fenghuang NO.51	table	
SRR5891826	*Vitis vinifera* ssp. *vinifera*	Hetianhong	table	
SRR5891828	*Vitis vinifera* ssp. *vinifera*	Chasselas Rouge	table	
SRR5891829	*Vitis vinifera* ssp. *vinifera*	Victoria	table	
SRR5891830	*Vitis vinifera* ssp. *vinifera*	Muscatny	table	
SRR5891831	*Vitis vinifera* ssp. *vinifera*	Otilia	table	
SRR5891832	*Vitis vinifera* ssp. *vinifera*	Tamina	table	
SRR5891834	*Vitis vinifera* ssp. *vinifera*	Augusta	table	
SRR5891835	*Vitis vinifera* ssp. *vinifera*	Zhengjia	table	
SRR5891836	*Vitis vinifera* ssp. *vinifera*	Zhengzhouzaohong	table	

SRR5891848	*Vitis vinifera* ssp. *vinifera*	Jingzijing	table	
SRR5891850	*Vitis vinifera* ssp. *vinifera*	Hiro Hamburg	table	
SRR5891853	*Vitis vinifera* ssp. *vinifera*	High Bailey	table	
SRR5891856	*Vitis vinifera* ssp. *vinifera*	Pirato	table	
SRR5891885	*Vitis vinifera* ssp. *vinifera*	Ribier Noir	table	
SRR5891895	*Vitis vinifera* ssp. *vinifera*	Pobeda	table	
SRR5891897	*Vitis vinifera* ssp. *vinifera*	Chasselas Blanc	table	
SRR5891898	*Vitis vinifera* ssp. *vinifera*	Uva Sacra	table	
SRR5891899	*Vitis vinifera* ssp. *vinifera*	Volga Don	table	
SRR5891905	*Vitis vinifera* ssp. *vinifera*	Meisibisan	table	
SRR5891933	*Vitis vinifera* ssp. *vinifera*	Neomat	table	
SRR5891954	*Vitis vinifera* ssp. *vinifera*	Zaoyu	table	
SRR5891955	*Vitis vinifera* ssp. *vinifera*	Yunikonkaiji	table	
SRR5891956	*Vitis vinifera* ssp. *vinifera*	Wuhebaijixin Seedless	table	
SRR5891964	*Vitis vinifera* ssp. *vinifera*	Ballade	table	
SRR5891965	*Vitis vinifera* ssp. *vinifera*	Banana	table	
SRR5891971	*Vitis vinifera* ssp. *vinifera*	Alissa	table	
SRR5891975	*Vitis vinifera* ssp. *vinifera*	Baby finger	table	
SRR5891976	*Vitis vinifera* ssp. *vinifera*	Beni Pizzutello	table	
SRR5891978	*Vitis vinifera* ssp. *vinifera*	Pizzutello	table	
SRR5891982	*Vitis vinifera* ssp. *vinifera*	Benibato	table	
SRR5891990	*Vitis vinifera* ssp. *vinifera*	Muscat Blanc a Petits Grains	table	
SRR5891991	*Vitis vinifera* ssp. *vinifera*	Flame Muscat	table	
SRR5891993	*Vitis vinifera* ssp. *vinifera*	Queen of Vineyard	table	
SRR5892006	*Vitis vinifera* ssp. *vinifera*	Shohei Red	table	
SRR5892009	*Vitis vinifera* ssp. *vinifera*	Rosaki	table	
SRR5892026	*Vitis vinifera* ssp. *vinifera*	Jingcui	table	
SRR5892027	*Vitis vinifera* ssp. *vinifera*	Jingdajing	table	
SRR5892028	*Vitis vinifera* ssp. *vinifera*	Hongjixin	table	
SRR5892030	*Vitis vinifera* ssp. *vinifera*	Jingmi	table	
SRR5892032	*Vitis vinifera* ssp. *vinifera*	Jingfeng	table	
SRR5892036	*Vitis vinifera* ssp. *vinifera*	Jingxiu	table	
SRR5892038	*Vitis vinifera* ssp. *vinifera*	Tianshanputao	table	
SRR5892054	*Vitis vinifera* ssp. *vinifera*	Topaz	table	
SRR5892055	*Vitis vinifera* ssp. *vinifera*	Yunibarasebun	table	
SRR5892056	*Vitis vinifera* ssp. *vinifera*	Regina	table	
SRR5892057	*Vitis vinifera* ssp. *vinifera*	Bolgar	table	
SRR7662547	*Vitis vinifera* ssp. *vinifera*	Hongju	table	
SRR7662549	*Vitis vinifera* ssp. *vinifera*	Crimson Seedless	table	
SRR7662550	*Vitis vinifera* ssp. *vinifera*	Dawn Seedless	table	
SRR7662551	*Vitis vinifera* ssp. *vinifera*	Fantasy Seedless	table	
SRR7662552	*Vitis vinifera* ssp. *vinifera*	Autumn Royal	table	
SRR7662553	*Vitis vinifera* ssp. *vinifera*	Autumn Seedless	table	

SRR7662558	*Vitis vinifera* ssp. *vinifera*	Italia	table	
SRR7662559	*Vitis vinifera* ssp. *vinifera*	Kishmish Chernyi	table	
SRR7662561	*Vitis vinifera* ssp. *vinifera*	Perlon	table	
SRR7662562	*Vitis vinifera* ssp. *vinifera*	Princess	table	
SRR7662564	*Vitis vinifera* ssp. *vinifera*	Hur Seedless	table	
SRR7662565	*Vitis vinifera* ssp. *vinifera*	Ruby Seedless	table	
SRR8618111	*Vitis vinifera* ssp. *vinifera*	Rizamat	table	
SRR8835158	*Vitis vinifera* ssp. *vinifera*	Medouar	table	
SRR8835159	*Vitis vinifera* ssp. *vinifera*	Orlovi nogti	table	
SRR8835161	*Vitis vinifera* ssp. *vinifera*	Maska	table	
SRR931841	*Vitis vinifera* ssp. *vinifera*	Sultanina	table	
P52	*Vitis vinifera* ssp. *vinifera*	unknow	table	Sequenced in this study
ERR514999	*Vitis vinifera* ssp. *vinifera*	Gewurztraminer	wine	
SAMN04589871	*Vitis vinifera* ssp. *vinifera*	Cabernet Sauvignon FPS 08	wine	
SAMN04995072	*Vitis vinifera* ssp. *vinifera*	Pinot gris	wine	
SAMN04995073	*Vitis vinifera* ssp. *vinifera*	Pinot blanc	wine	
SAMN04995074	*Vitis vinifera* ssp. *vinifera*	Pinot Meunier	wine	
SAMN05463241	*Vitis vinifera* ssp. *vinifera*	Pinot Noir	wine	
SRR5506711	*Vitis vinifera* ssp. *vinifera*	Sangiovese	wine	
SRR11078314	*Vitis vinifera* ssp. *vinifera*	Semillon	wine	
SRR12265742	*Vitis vinifera* ssp. *vinifera*	Bituni-T	wine	
SRR12265748	*Vitis vinifera* ssp. *vinifera*	Yael	wine	
SRR12265762	*Vitis vinifera* ssp. *vinifera*	Ramtania	wine	
SRR12265770	*Vitis vinifera* ssp. *vinifera*	Marawi	wine	
SRR12265792	*Vitis vinifera* ssp. *vinifera*	Dabuki-M	wine	
SRR12328988	*Vitis vinifera* ssp. *vinifera*	Petite-Sirah	wine	
SRR12328989	*Vitis vinifera* ssp. *vinifera*	Carignan	wine	
SRR12328990	*Vitis vinifera* ssp. *vinifera*	French-colombard	wine	
SRR12328991	*Vitis vinifera* ssp. *vinifera*	Shiraz	wine	
SRR2015301	*Vitis vinifera* ssp. *vinifera*	Riesling	wine	
SRR2895164	*Vitis vinifera* ssp. *vinifera*	Tempranillo Tinto	wine	
SRR2895165	*Vitis vinifera* ssp. *vinifera*	Tempranillo Blanco	wine	
SRR3496931	*Vitis vinifera* ssp. *vinifera*	Nitzanim_Bustan	wine	
SRR3508973	*Vitis vinifera* ssp. *vinifera*	Nitzan3	wine	
SRR5626056	*Vitis vinifera* ssp. *vinifera*	Nebbiolo_CVT71	wine	
SRR5627798	*Vitis vinifera* ssp. *vinifera*	Gamay Noir3	wine	
SRR5627800	*Vitis vinifera* ssp. *vinifera*	Aramon	wine	
SRR5627802	*Vitis vinifera* ssp. *vinifera*	Traminer 1	wine	
SRR5803836	*Vitis vinifera* ssp. *vinifera*	Cannonau	wine	
SRR5803837	*Vitis vinifera* ssp. *vinifera*	Bovale Sardo	wine	
SRR5803838	*Vitis vinifera* ssp. *vinifera*	Vermentino	wine	
SRR5803839	*Vitis vinifera* ssp. *vinifera*	Carignano	wine	
SRR5891601	*Vitis vinifera* ssp. *vinifera*	Riesling Lion	wine	

SRR5891616	*Vitis vinifera* ssp. *vinifera*	Kirovabadsky	wine	
SRR5891619	*Vitis vinifera* ssp. *vinifera*	Bayan Shirei	wine	
SRR5891620	*Vitis vinifera* ssp. *vinifera*	Silvaner Gruen	wine	
SRR5891635	*Vitis vinifera* ssp. *vinifera*	Cubupbkabbiu	wine	
SRR5891643	*Vitis vinifera* ssp. *vinifera*	Bujiesuli	wine	
SRR5891695	*Vitis vinifera* ssp. *vinifera*	Yan 73	wine	
SRR5891773	*Vitis vinifera* ssp. *vinifera*	Aligote	wine	
SRR5891775	*Vitis vinifera* ssp. *vinifera*	Cabernet Gernischet	wine	
SRR5891783	*Vitis vinifera* ssp. *vinifera*	Blue French	wine	
SRR5891859	*Vitis vinifera* ssp. *vinifera*	Rkatsiteli	wine	
SRR5891860	*Vitis vinifera* ssp. *vinifera*	Rkatsiteli vardisperi	wine	
SRR5891862	*Vitis vinifera* ssp. *vinifera*	Mzwane	wine	
SRR5891863	*Vitis vinifera* ssp. *vinifera*	Syrah	wine	
SRR5891884	*Vitis vinifera* ssp. *vinifera*	Riesling Italian	wine	
SRR5891958	*Vitis vinifera* ssp. *vinifera*	Yaniu	wine	
SRR5891961	*Vitis vinifera* ssp. *vinifera*	Ugni Blanc	wine	
SRR5891983	*Vitis vinifera* ssp. *vinifera*	Benibarado	wine	
SRR5891992	*Vitis vinifera* ssp. *vinifera*	Barbera nera	wine	
SRR5892058	*Vitis vinifera* ssp. *vinifera*	Maiskii Chernyi	wine	
SRR5892059	*Vitis vinifera* ssp. *vinifera*	Kabassia	wine	
SRR5892070	*Vitis vinifera* ssp. *vinifera*	Yuelianhua	wine	
SRR7188231	*Vitis vinifera* ssp. *vinifera*	Gouais blanc	wine	
SRR8506855	*Vitis vinifera* ssp. *vinifera*	Carmenere	wine	
SRR863618	*Vitis vinifera* ssp. *vinifera*	tannat	wine	
SRR8835140	*Vitis vinifera* ssp. *vinifera*	Tsolikoouri	wine	
SRR8835147	*Vitis vinifera* ssp. *vinifera*	Jaen	wine	
SRR8835148	*Vitis vinifera* ssp. *vinifera*	Heben	wine	
SRR8835150	*Vitis vinifera* ssp. *vinifera*	Espadeiro tinto	wine	
SRR8835152	*Vitis vinifera* ssp. *vinifera*	Colorino	wine	
SRR8835156	*Vitis vinifera* ssp. *vinifera*	Savagnin	wine	
SRR8835160	*Vitis vinifera* ssp. *vinifera*	Malvasia di Sardegna	wine	
SRR8835163	*Vitis vinifera* ssp. *vinifera*	Listan Prieto	wine	
SRR8835169	*Vitis vinifera* ssp. *vinifera*	Chouchillon	wine	
SRR8835172	*Vitis vinifera* ssp. *vinifera*	Araklinos	wine	
SRR8835173	*Vitis vinifera* ssp. *vinifera*	Castellana Blanca	wine	
SRR8835174	*Vitis vinifera* ssp. *vinifera*	Carignan noir	wine	
SRR5627801	*Vitis vinifera* ssp. *vinifera*	Zinfandel	wine	
Aladasturi	*Vitis vinifera* ssp. *vinifera*	Aladasturi	wine	Sequenced in this study
CabernetFranc	*Vitis vinifera* ssp. *vinifera*	Cabernet Franc	wine	Sequenced in this study
Chardonnay	*Vitis vinifera* ssp. *vinifera*	Chardonnay	wine	Sequenced in this study
Krakhuna	*Vitis vinifera* ssp. *vinifera*	Krakhuna	wine	Sequenced in this study
Meskhuri_mtsvane	*Vitis vinifera* ssp. *vinifera*	N4_Meskhuri_mtsvane	wine	Sequenced in this study
P20	*Vitis vinifera* ssp. *vinifera*	Merlot 37	wine	Sequenced in this study

P49	*Vitis vinifera* ssp. *vinifera*	Sauvignon blanc 30	wine	Sequenced in this study
Saperavi_NoIndex	*Vitis vinifera* ssp. *vinifera*	Saperavi_NoIndex	wine	Sequenced in this study
Tsitska	*Vitis vinifera* ssp. *vinifera*	Tsitska	wine	Sequenced in this study

Table S2. ABS(Δlhood) and AIC values of different models of each population independently. The columns with red words are the best-fitted models for each population.

	MODEL	CONSTANT	EXPANSION	CONTRACTION	BOTTLECK_S	BOTTLENECK
**EU**	**ABS(Δlhood)**	7456732.447	7234413.917	7421950.694	7212564.186	7200189.997
	**AIC**	68205901.11	67182090.44	68045729.22	67081468.71	67024487.47
**ME1**	**ABS(Δlhood)**	6940359.55	6784846.964	6770245.43	6769868.648	6770071.202
	**AIC**	65064797.73	64348639.81	64281397.26	64279662.11	64280598.91
**ME2**	**ABS(Δlhood)**	8046713.117	7994961.848	7992369.006	7992463.588	7992494.535
	**AIC**	55139231.52	54900912.12	54888971.64	54889407.21	54889553.72
**TABLE**	**ABS(Δlhood)**	6490533.83	6198549.458	6146116.176	6144224.959	6144199.165
	**AIC**	81574927.42	80230293.7	79988829.51	79980120.13	79980005.35
**WINE**	**ABS(Δlhood)**	6869340.919	6655840.411	6655534.405	6637858.025	6637791.044
	**AIC**	77180647.56	76197445.39	76196036.18	76114633.44	76114328.98

Table S3. ABS (Δlhood) and AIC values of different models for estimation of the process of domestication when gene flow was not considered. The row with red words is the best-fitted model.

MODEL	ABS(Δlhood)	AIC
**Model_1**	1069494.16	99655782.06
**Model_2**	1025803.44	99454578.82
**Model_3**	1132936.24	99947943.62
**Model_4**	763183.71	78377756.95
**Model_5**	763809.60	78380639.26
**Model_6**	851572.49	78784802.31

Table S4. ABS (Δlhood) and AIC values of different models for estimation of the process of gene flow among the table, wine and EU populations. The row with red words is the best-fitted model.

	MODEL	ABS(Δlhood)	AIC		MODEL	ABS(Δlhood)	AIC
ME1 population was assumed as the ancestral population	T1	2750515	280755024	ME2 population was assumed as the ancestral population	T1	2828129	281112451
	T2	2755265	280776903		T2	2829915	281120678
	T3	2753987	280771019		T3	2827604	281110036
	T4	2779321	280887677		T4	2867003	281291470
	T5	2756660	280783319		T5	2833582	281137560
	T6	2746595	280736971		T6	2823161	281089571
	T7	2753192	280767352		T7	2827709	281110517
	T8	2751363	280758928		T8	2825013	281098099
	T9	2891353	281403602		T9	2963536	281736015
	T10	2801137	280988141		T10	2880103	281351791
	T11	2762098	280808359		T11	2835584	281146777
	T12	2717711	280603953		T12	2798763	280977213
	T13	2718007	280605321		T13	2794518	280957667
	T14	2720089	280614905		T14	2804349	281002937
	T15	2763735	280815897		T15	2850952	281217548
	T16	2742439	280717825		T16	2830240	281122167
	T17	3235519	282988536		T17	3342765	283482422

Table S5. Gene flow between domesticated grapes and EU wild grapes evaluated by fastsimcoal.

From	To	Migration rates
EU	table	3.8×10^{-5}
table	EU	2.9×10^{-6}
EU	wine	1.7×10^{-4}
wine	EU	1.1×10^{-4}

* Continuous gene flow between EU and domesticated grapes started 1.8×10^{3} years ago until the present.

Table S6. The statistic of fdM analysis with different population combinations

P1	P2	P3	fdM>0	fdM<O
ME2	wine	EU	6049	840
ME2	table	EU	4786	2128
table	wine	EU	6743	651

* fdM>0: accommodate admixture between P2 and P3
fdM<0: accommodate admixture between P1 and P3
The number was the windows counted (50 kb per window)

Table S7. Average recombination rates of different regions and values relative to the genome-wide estimated rate

Regions	cM/kb	Relative to genome-wide
Genome-wide	0.0066	-
5% highest fd	0.0066	1.00
1% highest fd	0.0064	0.97
Hard selection	0.0097	1.47
Soft selection	0.0083	1.25

Tables S8. The rank of statistical parameters by weight for introgression prediction in Filet

Rank	Feature	Weight
**1**	thetaH2	0.168337
**2**	pi2	0.829879
**3**	H2	0.823987
**4**	private1	0.535958
**5**	dxy_mean	0.533798
**6**	dxy_min	0.519373
**7**	ss2	0.434459
**8**	HapCount2	0.42505
**9**	ZnS2	0.423541
**10**	snn	0.389743
**11**	ZnS1	0.387115
**12**	hetVar2	0.361244
**13**	F_{ST}	0.345485
**14**	tajd1	0.343798
**15**	ss1	0.314636
**16**	thetaH1	0.287917
**17**	H1	0.250295
**18**	pi1	0.237761
**19**	tajd2	0.231185
**20**	private2	0.231075
**21**	HapCount1	0.208633
**22**	hetVar1	0.201694

Table S9. Three kinds of genes located both in introgression regions and selected or linked selected regions.

ID	name	Pfam_name	GO
H0013F_015.ver1.0.g085360	F1	NB-ARC domain	GO:0005524,GO:0043531,GO:0006952
P0092F.ver1.0.g479620	F2	NB-ARC domain	GO:0005524,GO:0043531,GO:0006952
P0092F.ver1.0.g479550	F3	NB-ARC domain	GO:0005524,GO:0043531,GO:0006952
P0184F.ver1.0.g554800	F4	Leucine rich repeat	GO:0005886,GO:0050832,GO:0016021
P0054F.ver1.0.g432090	F5	Aspartic acid proteinase inhibitor	GO:0004869,GO:0005576,GO:0006952
P0051F.ver1.0.g426160	F6	SNF2-related domain	GO:0016787,GO:0070615,GO:0005634,GO:0006306,GO:0090577,GO:0005524,GO:0004386,GO:0071360,GO:0005721,GO:0060966,GO:1900370,GO:0050832,GO:0035563,GO:0060964,GO:0080188,GO:1900111,GO:0032776,GO:0000419,GO:0003677,GO:0070829,GO:0060968,GO:1900368
P0051F.ver1.0.g426170	F7	Leucine Rich Repeat	GO:0031349,GO:0106311,GO:0004674,GO:0005886,GO:0006952,GO:0005789,GO:0005524,GO:0016021,GO:1900426,GO:0106310,GO:0046777,GO:0005634
P0031F.ver1.0.g394460	F8	N-terminal domain of argonaute	GO:0006417,GO:0005829,GO:0003729,GO:0035197,GO:0042742,GO:0051607,GO:0019048,GO:0031047,GO:0005634
P0107F.ver1.0.g497850	F9	Protein tyrosine and serine/threonine kinase	GO:0106311,GO:0004674,GO:0005886,GO:0006952,GO:0045087,GO:0005524,GO:0004672,GO:0042742,GO:0002221,GO:0106310,GO:0046777
P0079F.ver1.0.g460600	F10	Leucine rich repeat N-terminal domain	GO:0042802,GO:0005102,GO:0033612,GO:0004675,GO:0106311,GO:0004714,GO:0005886,GO:0006952,GO:0005524,GO:0106310,GO:0010008,GO:0016021
P0015F.ver1.0.g343650	F11	Leucine rich repeat	GO:0005634,GO:0005737,GO:0050135,GO:0043068,GO:0007165,GO:0050832,GO:0043531,GO:0042803,GO:0061809
P0142F.ver1.0.g529280	F12	Rx N-terminal domain	GO:0005524,GO:0043531,GO:0006952
P0018F.ver1.0.g349190	F13	NAD(P)H-binding	GO:0005840,GO:0009409,GO:0005996,GO:0010287,GO:0045893,GO:0010297,GO:0006364,GO:0009506,GO:0010319,GO:0045727,GO:0019843,GO:0009507,GO:0003824,GO:0010468,GO:0048046,GO:0000427,GO:0009570,GO:0005773,GO:0005829,GO:0003729,GO:0007623,GO:0005777,GO:0009611,GO:0009941,GO:0042631,GO:0005576,GO:0003723,GO:0000272,GO:0042742,GO:0003677,GO:0009658,GO:0032544
P0035F.ver1.0.g400900	F14	NA	GO:0048443,GO:0010152,GO:0080022,GO:0071249,GO:0019005,GO:0006952,GO:0010011,GO:0000822,GO:0016567,GO:0048527,GO:0009734,GO:0031146,GO:0005634

P0035F.ver1.0.g400360	F15	Protein tyrosine and serine/threonine kinase	GO:0010483,GO:0009788,GO:0046777,GO:0009738,GO:0016021,GO:0010118,GO:0009873,GO:0009506,GO:0106311,GO:0005524,GO:0048364,GO:0030308,GO:0009723,GO:0106310,GO:0009742,GO:0050832,GO:0009741,GO:0009791,GO:0043680,GO:0005886,GO:0004672,GO:0032922
P0188F.ver1.0.g556500	F16	short chain dehydrogenase	GO:0005737,GO:0016020,GO:0006952,GO:0047501
P0095F.ver1.0.g483710	F17	Leucine Rich Repeat	GO:0005886,GO:0050832,GO:0016021
H0125F_007.ver1.0.g219690	F18	Leucine rich repeat	GO:0005886,GO:0050832,GO:0016021
P0009F.ver1.0.g320120	F19	Leucine Rich Repeat	GO:0031349,GO:0106311,GO:0004674,GO:0005886,GO:0006952,GO:0005789,GO:0005524,GO:0016021,GO:1900426,GO:0106310,GO:0046777,GO:0005634
P0009F.ver1.0.g319510	F20	Protein-tyrosine phosphatase	GO:0005737,GO:0033550,GO:0005886,GO:0006952,GO:0004725,GO:0035556,GO:0005634,GO:0031348,GO:0019900
P0225F.ver1.0.g573150	F21	Leucine Rich Repeat	GO:0005634,GO:0005737,GO:0050135,GO:0043068,GO:0007165,GO:0050832,GO:0043531,GO:0042803,GO:0061809
P0481F.ver1.0.g613060	F22	NB-ARC domain	GO:0005634,GO:0005737,GO:0050135,GO:0043068,GO:0007165,GO:0050832,GO:0043531,GO:0042803,GO:0061809
P0175F.ver1.0.g551160	F23	C-JID domain	GO:0005737,GO:0050135,GO:0009626,GO:0005524,GO:0043531,GO:0007165,GO:0061809
P0073F.ver1.0.g455540	F24	NB-ARC domain	GO:0005737,GO:0050135,GO:0003953,GO:0007165,GO:0061809,GO:0050832,GO:0019677,GO:0043531,GO:0043068,GO:0005634
P0073F.ver1.0.g455530	F25	C-JID domain	GO:0050135,GO:0009626,GO:0005524,GO:0051607,GO:0009615,GO:0043531,GO:0007165,GO:0061809
P0071F.ver1.0.g451970	F26	NB-ARC domain	GO:0005634,GO:0005737,GO:0050135,GO:0043068,GO:0007165,GO:0050832,GO:0043531,GO:0042803,GO:0061809
P0071F.ver1.0.g452090	F27	C-JID domain	GO:0005737,GO:0050135,GO:0009626,GO:0005524,GO:0043531,GO:0007165,GO:0061809
P0007F.ver1.0.g311640	A1	non-haem dioxygenase in morphine synthesis N-terminal	GO:0005829,GO:0009694,GO:0009696,GO:0071456,GO:0080032,GO:0080030,GO:0016788,GO:0080031
P0139F.ver1.0.g526650	A2	Myb-like DNA-binding domain	GO:0000987,GO:0045723,GO:0003700,GO:0000976,GO:0010345,GO:0005634
H0113F_005.ver1.0.g211860	A3	NA	GO:0009698,GO:0016592,GO:2000762
P0107F.ver1.0.g497980	A4	Peroxidase	GO:0005829,GO:1901430,GO:0006979,GO:0005618,GO:0005794,GO:0020037,GO:0010089,GO:0042744,GO:0004601,GO:0009809,GO:0009505,GO:0048046,GO:0046872
P0115F.ver1.0.g504600	A5	EPSP synthase	GO:0010597,GO:0009073,GO:0009635,GO:0009507,GO:0007623,GO:0008652,GO:0003866,GO:0009423

P0115F.ver1.0.g504610	A6	EPSP synthase	GO:0009073,GO:0009507,GO:0008652,GO:0003866,GO:0009423
P0148F.ver1.0.g533120	A7	Aromatic amino acid lyase	GO:0009800,GO:0006559,GO:0005737,GO:0045548
P0148F.ver1.0.g533100	A8	Aromatic amino acid lyase	GO:0009800,GO:0006559,GO:0005737,GO:0045548
P0148F.ver1.0.g533090	A9	Aromatic amino acid lyase	GO:0009800,GO:0006559,GO:0005737,GO:0045548
P0192F.ver1.0.g558980	A10	Multicopper oxidase	GO:0016491,GO:0052716,GO:0005507,GO:0009813,GO:0046274,GO:0010023,GO:0009809,GO:0046688,GO:0048046
P0328F.ver1.0.g595970	A11	Multicopper oxidase	GO:0016491,GO:0052716,GO:0005507,GO:0046274,GO:0048046
P0328F.ver1.0.g595980	A12	Multicopper oxidase	GO:0016491,GO:0052716,GO:0005507,GO:0046274,GO:0048046
H0034F_028.ver1.0.g136440	A13	Multicopper oxidase	GO:0016491,GO:0052716,GO:0005507,GO:0046274,GO:0048046
P0225F.ver1.0.g572920	A14	Multicopper oxidase	GO:0016491,GO:0052716,GO:0005507,GO:0046274,GO:0048046
P0225F.ver1.0.g572860	A15	Multicopper oxidase	GO:0016491,GO:0052716,GO:0005507,GO:0046274,GO:0048046
P0481F.ver1.0.g613040	A16	Multicopper oxidase	GO:0016491,GO:0052716,GO:0005507,GO:0046274,GO:0048046
P0248F.ver1.0.g578660	A17	Multicopper oxidase	GO:0016491,GO:0052716,GO:0005507,GO:0046274,GO:0048046
P0248F.ver1.0.g578420	A18	Multicopper oxidase	GO:0016491,GO:0052716,GO:0005507,GO:0009813,GO:0046274,GO:0010023,GO:0009809,GO:0046688,GO:0048046
H0175F_004.ver1.0.g237880	A19	Multicopper oxidase	GO:0016491,GO:0052716,GO:0005507,GO:0009813,GO:0046274,GO:0010023,GO:0009809,GO:0046688,GO:0048046
P0175F.ver1.0.g551170	A20	Multicopper oxidase	GO:0016491,GO:0052716,GO:0005507,GO:0046274,GO:0048046
P0071F.ver1.0.g452100	A21	NA	GO:0004838,GO:0006520,GO:0005829,GO:0030170,GO:0006572,GO:0006559,GO:0009536,GO:0009058
H0006F_033.ver1.0.g061150	S1	FAT domain	GO:0009860,GO:0045332,GO:1905038,GO:0005886,GO:0012505,GO:0140326,GO:0005524,GO:0000287,GO:0016021
P0028F.ver1.0.g383070	S2	Protein kinase domain	GO:0106311,GO:0004674,GO:0005886,GO:0048544,GO:0006468,GO:0005524,GO:0005516,GO:0030246,GO:0106310,GO:0016021
P0031F.ver1.0.g393160	S3	D-mannose binding lectin	GO:0106311,GO:0004674,GO:0005886,GO:0048544,GO:0005524,GO:0005516,GO:0030246,GO:0106310,GO:0016021
P0118F.ver1.0.g509090	S4	Leucine rich repeat	GO:0090406,GO:0009506,GO:0010183,GO:0106311,GO:0005886,GO:0005524,GO:0009755,GO:0106310,GO:0016021

P0118F.ver1.0.g509100	S5	Leucine rich repeat N-terminal domain	GO:0090406,GO:0009506,GO:0010183,GO:0106311,GO:0005886,GO:0005524,GO:0009755,GO:0106310,GO:0016021
P0035F.ver1.0.g400360	S6	Protein tyrosine and serine/threonine kinase	GO:0010483,GO:0009788,GO:0046777,GO:0009738,GO:0016021,GO:0010118,GO:0009873,GO:0009506,GO:0106311,GO:0005524,GO:0048364,GO:0030308,GO:0009723,GO:0106310,GO:0009742,GO:0050832,GO:0009741,GO:0009791,GO:0043680,GO:0005886,GO:0004672,GO:0032922
P0024F.ver1.0.g367980	S7	D-mannose binding lectin	GO:0005773,GO:0004675,GO:0009506,GO:0106311,GO:0004674,GO:0005886,GO:0031625,GO:0048544,GO:0006468,GO:0005524,GO:0030246,GO:0106310,GO:0016021
P0024F.ver1.0.g367970	S8	D-mannose binding lectin	GO:0005773,GO:0004675,GO:0009506,GO:0106311,GO:0004674,GO:0005886,GO:0031625,GO:0048544,GO:0006468,GO:0005524,GO:0030246,GO:0106310,GO:0016021
P0024F.ver1.0.g367960	S9	Protein kinase domain	GO:0009506,GO:0106311,GO:0004674,GO:0005886,GO:0031625,GO:0048544,GO:0006468,GO:0005524,GO:0005516,GO:0030246,GO:0106310,GO:0016021
P0024F.ver1.0.g367950	S10	S-locus glycoprotein domain	GO:0106311,GO:0004674,GO:0005886,GO:0048544,GO:0006468,GO:0005524,GO:0005516,GO:0030246,GO:0106310
H0047F_007.ver1.0.g155080	S11	S-locus glycoprotein domain	GO:0106311,GO:0004674,GO:0005886,GO:0048544,GO:0006468,GO:0005524,GO:0005516,GO:0030246,GO:0106310

Table S10. The merged introgressed regions.

Chromosome	Start	End	Span
1	1230000	1280000	50000
1	1700000	1750000	50000
1	2040000	2070000	30000
3	4510000	4540000	30000
3	5960000	5990000	30000
3	6640000	6670000	30000
3	8940000	8970000	30000
3	12480000	12520000	40000
3	12760000	12810000	50000
3	13530000	13630000	100000
3	15410000	15450000	40000
3	15520000	15550000	30000
3	15680000	15830000	150000
3	15910000	15940000	30000
3	17300000	17320000	20000
4	18780000	18830000	50000
4	23830000	23860000	30000
5	1640000	1660000	20000
5	1800000	1870000	70000
5	6410000	6470000	60000
5	17300000	17320000	20000
6	530000	550000	20000
6	10780000	10800000	20000
6	15350000	15370000	20000
6	15430000	15490000	60000
6	15690000	15750000	60000
7	2120000	2210000	90000
7	15960000	15990000	30000
7	16370000	16430000	60000
7	16710000	16850000	140000
7	17890000	18000000	110000
7	20040000	20060000	20000
7	24300000	24320000	20000
9	870000	930000	60000
9	7880000	7900000	20000
9	7970000	7990000	20000
9	8170000	8250000	80000
9	8830000	8880000	50000
9	8940000	9070000	130000
9	9160000	9210000	50000
9	10050000	10100000	50000

9	11960000	11990000	30000
9	12070000	12130000	60000
9	12980000	13080000	100000
9	16300000	16350000	50000
10	2820000	2890000	70000
10	3330000	3350000	20000
10	4300000	4440000	140000
10	4500000	4600000	100000
10	10020000	10050000	30000
10	11100000	11130000	30000
10	13400000	13460000	60000
10	14170000	14420000	250000
10	14480000	14550000	70000
10	18180000	18250000	70000
10	18330000	18410000	80000
10	18470000	18520000	50000
10	19050000	19090000	40000
10	22010000	22060000	50000
10	23170000	23230000	60000
11	1380000	1400000	20000
11	8930000	8950000	20000
12	2510000	2670000	160000
12	2770000	2840000	70000
12	7990000	8050000	60000
12	8570000	8620000	50000
12	16600000	16650000	50000
12	16870000	16900000	30000
12	17050000	17070000	20000
12	18270000	18320000	50000
12	18480000	18510000	30000
12	18730000	18780000	50000
12	18850000	18920000	70000
12	19770000	19810000	40000
12	19940000	20030000	90000
12	21380000	21420000	40000
12	21840000	21860000	20000
12	22620000	22680000	60000
12	22840000	22880000	40000
12	23450000	23540000	90000
13	16340000	16360000	20000
13	23150000	23230000	80000
13	26170000	26200000	30000
13	27250000	27310000	60000

14	10000	50000	40000
14	1010000	1060000	50000
14	3010000	3080000	70000
14	14650000	14670000	20000
14	15280000	15400000	120000
14	17810000	17860000	50000
15	4490000	4520000	30000
15	4640000	4680000	40000
15	5240000	5310000	70000
15	5600000	5650000	50000
15	12160000	12180000	20000
15	13360000	13430000	70000
15	14230000	14270000	40000
16	360000	410000	50000
16	540000	700000	160000
16	790000	830000	40000
16	7620000	7680000	60000
16	8490000	8560000	70000
16	11580000	11630000	50000
16	18900000	18960000	60000
16	21550000	21570000	20000
16	22220000	22340000	120000
16	22740000	22760000	20000
18	16680000	16700000	20000
18	17810000	17850000	40000
18	19120000	19160000	40000
18	19510000	19630000	120000
18	19810000	19980000	170000
18	20160000	20220000	60000
18	20370000	20430000	60000
18	22360000	22390000	30000
18	23900000	23960000	60000
18	24340000	24440000	100000
18	25030000	25180000	150000
18	25300000	25480000	180000
18	25680000	25760000	80000
18	26140000	26240000	100000
18	26540000	26680000	140000
18	26940000	26980000	40000
18	27960000	28140000	180000
18	28240000	28450000	210000
18	28550000	28620000	70000
18	29110000	29130000	20000

18	29890000	29910000	20000
18	30300000	30360000	60000
18	31390000	31410000	20000
18	31890000	32030000	140000
18	32100000	32320000	220000
18	33320000	33340000	20000
18	34890000	34910000	20000
19	4070000	4170000	100000
19	4500000	4550000	50000
19	4810000	4900000	90000
19	6460000	6510000	50000
19	7460000	7510000	50000
19	8490000	8520000	30000
19	13060000	13080000	20000
19	14140000	14180000	40000
19	14510000	14540000	30000
19	14600000	14640000	40000
19	16660000	16680000	20000
19	17160000	17180000	20000

Movie S1 (separate file). The SFS change of introgressed alleles after hybridization in outcrossing population. The bar located directly above the movie indicates the generations after hybridization.

Movie S2 **(separate file).** The SFS change of introgressed alleles after hybridization in clonal population. The bar located directly above the movie indicates the generations after hybridization.

Movie S3 **(separate file).** The DFE change of introgressed alleles after hybridization. The bar located directly above the movie indicates the generations after hybridization.

Dataset S1 (separate file). Prediction of selective sweep regions on the genome of wine population by Shic.

Dataset S2 (separate file). Prediction of selective sweep regions on the genome of table population by Shic.

Dataset S3 (separate file). Prediction of introgressed regions between EU and wine population by Filet. Class 1 means introgression from EU to wine population, and class 2 indicates the opposite.

Dataset S4 (separate file). The genes located in introgressed regions and the selective types of these regions in wine population. Class 1 means introgression from EU to wine population, and class 2 indicates the opposite.

SI References

1. H. Xiao *et al.*, genome wide sequencing of *Vitis* sp. NCBI BioProject. https://dataview.ncbi.nlm.nih.gov/object/PRJNA910315. Deposited 9 December 2022.
2. H. Xiao *et al.*, genome wide sequencing of *Vitis* sp. NGDC BioProject. https://ngdc.cncb.ac.cn/gsub. Deposited 28 April 2023.
3. Steven J. Phillips, Miroslav Dudík, Robert E. Schapire. Maxent software for modeling species niches and distributions (Version 3.4.1). Available from url: http://biodiversityinformatics.amnh.org/open_source/maxent/.
4. Vitis vinifera L. in GBIF Secretariat (2021). GBIF Backbone Taxonomy. Checklist dataset https://doi.org/10.15468/39omei accessed via GBIF.org on 2021-12-17.
5. Bolger AM, Lohse M, Usadel B (2014) Trimmomatic: a flexible trimmer for Illumina sequence data. *Bioinformatics (Oxford, England)* 30:2114–2120.
6. Zhou Y *et al. (*2019) The population genetics of structural variants in grapevine domestication. *Nature plants* 5:965–979.
7. Vasimuddin Md *et al.* (2019) Efficient Architecture-Aware Acceleration of BWA-MEM for Multicore Systems. *IEEE Parallel and Distributed Processing Symposium* (IPDPS).
8. McKenna A *et al. (*2010) The Genome Analysis Toolkit: A MapReduce framework for analyzing next-generation DNA sequencing data. *Genome Res.* 20:1297–1303.
9. Danecek P *et al. (*2011) The variant call format and VCFtools. *Bioinformatics (Oxford, England)* 27:2156–2158.
10. Browning BL, Tian X, Zhou Y, Browning SR (2021) Fast two-stage phasing of large-scale sequence data. *American journal of human genetics* 108:1880–1890.

11. Minh BQ *et al.* (2020) IQ-TREE 2: New Models and Efficient Methods for Phylogenetic Inference in the Genomic Era. *Molecular biology and evolution* 37:1530–1534.

12. Huson DH, Bryant D (2006) Application of phylogenetic networks in evolutionary studies. *Molecular biology and evolution* 23:254–267.

13. Alexander DH, Novembre J, Lange K (2009) Fast model-based estimation of ancestry in unrelated individuals. *Genome research* 19:1655–1664.

14. Chang CC *et al. (*2015) Second-generation PLINK: rising to the challenge of larger and richer datasets. *GigaScience* 4:7.

15. Haller BC, Messer PW (2019) SLiM 3: Forward Genetic Simulations Beyond the Wright-Fisher Model. *Molecular biology and evolution* 36:632–637.

16. H. Xiao *et al.*, analysis-code-for-introgression-in-grapes. GitHub. https://github.com/zhouyflab/Grapevine_Adaptive_Maladaptive_Introgression. Deposited 11 December 2022.

17. Terhorst J, Kamm JA, Song YS (2017) Robust and scalable inference of population history from hundreds of unphased whole genomes. *Nature genetics* 49:303–309.

18. Ossowski S *et al. (*2010) The rate and molecular spectrum of spontaneous mutations in Arabidopsis thaliana. *Science (New York, N.Y.)* 327:92–94.

19. Excoffier L, Dupanloup I, Huerta-Sánchez E, Sousa VC, Foll M (2013) Robust demographic inference from genomic and SNP data. *PLoS genetics* 9:e1003905.

20. Excofffier L *et al. (*2021) fastsimcoal2: demographic inference under complex evolutionary scenarios. *Bioinformatics (Oxford, England)*.

21. Vaser R, Adusumalli S, Leng SN, Sikic M, Ng PC (2016) SIFT missense predictions for genomes. *Nature protocols* 11:1–9.

22. The UniProt Consortium (2021) UniProt: the universal protein knowledgebase in 2021, *Nucleic Acids Research*, 49(D1): D480–D489.

23. Zhang C, Dong S-S, Xu J-Y, He W-M, Yang T-L (2019) PopLDdecay: a fast and effective tool for linkage disequilibrium decay analysis based on variant call format files. *Bioinformatics (Oxford, England)* 35:1786–1788.

24. Hämälä T, Savolainen O (2019) Genomic patterns of local adaptation under gene flow in Arabidopsis lyrata. *Molecular biology and evolution*.

25. Schrider DR, Kern AD (2016) S/HIC: Robust Identification of Soft and Hard Sweeps Using Machine Learning. *PLoS genetics* 12:e1005928.

26. Kern AD, Schrider DR (2018) diploS/HIC: An Updated Approach to Classifying Selective Sweeps. *G3 (Bethesda, Md.)* 8:1959–1970.

27. Malinsky M, Matschiner M, Svardal H (2021) Dsuite - Fast D-statistics and related admixture evidence from VCF files. *Molecular ecology resources* 21:584–595.

28. Schrider DR, Ayroles J, Matute DR, Kern AD (2018) Supervised machine learning reveals introgressed loci in the genomes of Drosophila simulans and D. sechellia. *PLoS genetics* 14:e1007341.

29. Hudson RR (2002) Generating samples under a Wright-Fisher neutral model of genetic variation. *Bioinformatics (Oxford, England)* 18:337–338.

30. Daniel Garrigan, Anthony J Geneva (2014) msmove. Figshare. http://dx.doi.org/10.6084/m9.figshare.1060474

31. Morales-Cruz A *et al. (*2021) Extensive introgression among North American wild grapes (*Vitis*) fuels biotic and abiotic adaptation. *Genome Biol* 22:254

32. Zou C *et al. (*2020) Haplotyping the Vitis collinear core genome with rhAmpSeq improves marker transferability in a diverse genus. *Nature communications* 11:413.

33. Buchfink B, Reuter K, Drost H-G (2021) Sensitive protein alignments at tree-of-life scale using DIAMOND. *Nature methods* 18:366–368.

34. Gene Ontology Consortium (2021) The Gene Ontology resource: enriching a GOld mine. *Nucleic Acids Res*. 49(D1):D325-D334.

35. Ashburner M *et al. (*2000) Gene ontology: tool for the unification of biology. The Gene Ontology Consortium. *Nature genetics* 25:25–29.

36. Shannon P *et al.* (2003) Cytoscape: a software environment for integrated models of biomolecular interaction networks. *Genome Res.* 13:2498–2504.
